# Multiple allosteric sites allow for synergistic enhancement of GPCR signaling

**DOI:** 10.1126/sciadv.aeh3401

**Published:** 2026-10-21

**Authors:** Kai Wang, Snezana T. Dimova, John N. Hanson, Abhishek Thakur, Youwei Xu, Yini Liu, Amy E. Moritz, Alexander Goldberg, Bing Xie, Jayachandra Rayadurgam, Feijun Wang, Ashley N. Nilson, Kathryn D. Luderman, R. Benjamin Free, Wen Hu, Jian Zhang, H. Eric Xu, Kevin J. Frankowski, Yue Wang, Lei Shi, David R. Sibley, Youwen Zhuang

**Affiliations:** ^1^ State Key Laboratory of Drug Research, State Key Laboratory of Chemical Biology, Shanghai Institute of Materia Medica, Chinese Academy of Sciences, Shanghai 201203, China.; ^2^ University of Chinese Academy of Sciences, Beijing 100049, China.; ^3^ Molecular Neuropharmacology Section, National Institute of Neurological Disorders and Stroke, National Institutes of Health, Bethesda, MD, USA.; ^4^ Computational Chemistry and Molecular Biophysics Section, National Institute of Drug Abuse, National Institutes of Health, Baltimore, MD, USA.; ^5^ Department of Pharmaceutical and Artificial-Intelligence Sciences, School of Medicine, Shanghai Jiao Tong University, Shanghai 200025, China.; ^6^ UNC Eshelman School of Pharmacy, Chapel Hill, NC, USA.; ^7^ Artificial Intelligence Clinical Research Center for drug discovery, Shanghai Key Laboratory of Flexible Medical Robotics, Tongren Hospital, School of Medicine, Shanghai Jiao Tong University, Shanghai 200025, China.; ^8^ Key Laboratory of Cell Differentiation and Apoptosis of Chinese Ministry of Education, School of Medicine, Shanghai Jiao Tong University, Shanghai 200025, China.

## Abstract

Allosteric modulation of G protein–coupled receptors (GPCRs) offers promising therapeutic opportunities. Although multiple allosteric binding sites have been identified in GPCRs, whether and how these spatially distinct sites cooperate to regulate receptor activation remain poorly understood. Here, we elucidate the molecular mechanism of cooperative multisite allostery in the dopamine D1 receptor (D1R). We developed two novel D1R positive allosteric modulators (PAMs), UNC9815 and UNC10062, and used cryo–electron microscopy to structurally identify two previously uncharacterized D1R allosteric sites occupied by UNC9815/UNC10062 and another D1R PAM, BMS-A1. Both sites localize to the transmembrane helix (TM) 1 to 7 interface as vertically adjacent allosteric pockets organized in a spatially complementary manner. Our structures demonstrate the simultaneous binding of the chemically distinct PAMs (LY3154207, BMS-A1, and UNC9815/UNC10062) in three nonoverlapping pockets. Concurrent engagement of all three allosteric sites produces pronounced functional cooperativity, increasing dopamine potency by >1000-fold, thereby demonstrating that coordinated multisite modulation can achieve substantial pharmacological amplification beyond the limits imposed by single PAM administration. Molecular dynamics (MD) simulations revealed that multisite PAM engagement restricts intracellular loop 3 dynamics and stabilizes the TM7-NPxxY activation motif, effects not achieved by binding of LY3154207 alone, thereby biasing the receptor toward a more stabilized active conformation, and may also improve agonist potency by stabilizing extracellular loop 2. Together, these findings establish the structural framework for cooperative multisite allostery in a GPCR, revealing how distributed allosteric sites act in concert to amplify receptor activation and providing a conceptual foundation for developing innovative GPCR allosteric therapeutics.

## INTRODUCTION

G protein–coupled receptors (GPCRs) constitute the largest class of membrane receptor drug targets and mediate diverse physiological processes (*1*, *2*). Among them, dopamine receptors (DRs) play essential roles in dopaminergic neurotransmission and signaling (*3*, *4*). These receptors are classified into two subfamilies: D1-like receptors include the D_1_ dopamine receptor (D1R) and D5R that couple to G_s/olf_ proteins to stimulate adenylyl cyclase and increase cyclic adenosine 3′,5′-monophosphate (cAMP) production, whereas D2-like receptors (D2R, D3R, and D4R) activate G_i/o_ proteins to inhibit cAMP production (*3*, *4*). The D1R represents a critical therapeutic target due to its abundance in brain regions controlling movement, motivation, and cognition (*3*–*5*). The role of the D1R in neuropsychiatric disorders—including Parkinson’s disease (PD), schizophrenia, and Alzheimer’s disease (AD)—has established it as a major target for drug discovery (*3*, *4*, *6*, *7*).

Traditional drug development for GPCRs has predominantly focused on the orthosteric binding pocket (OBP), where endogenous agonists bind (*6*, *8*). However, this approach has encountered substantial hurdles in the development of D1R-targeted drugs (*6*, *8*). In conditions such as PD, where dopaminergic neuron degeneration leads to reduced striatal dopamine (DA) levels, orthosteric agonists of the D1R have been explored as therapeutic alternatives to the DA precursor l-DOPA (*3*, *9*). Unfortunately, their usage has been hindered by poor oral bioavailability, limited blood-brain barrier penetration, and insufficient subtype selectivity. These liabilities lead to adverse side effects including hypotension and tachyphylaxis, resulting in limited clinical applications centered around non–central nervous system (CNS) indications (*6*, *10*–*12*). To address these challenges, allosteric modulation has emerged as an innovative strategy to overcome these limitations in GPCR drug discovery (*13*–*15*). Unlike orthosteric ligands, allosteric modulators bind to distinct, less conserved receptor sites, modulating responses to endogenous ligands without competing for the OBP (*6*, *14*, *16*, *17*). Compared to orthosteric agonists, positive allosteric modulators (PAMs) of the D1R offer improved receptor subtype selectivity, saturable effects that mitigate the risk of overstimulation, and preservation of the spatiotemporal dynamics of DA signaling (*6*, *18*–*21*). These characteristics make D1R PAMs a promising therapeutic strategy for conditions such as PD or schizophrenia, where D1R stimulation may improve cognitive impairment (*17*, *20*, *22*). Notable progress has been made in finding D1R PAMs through high-throughput screening (HTS) (*6*, *23*–*25*), and several groups have identified D1R PAMs such as BMS-A1, LY3154207, and ASP4345, some of which have advanced to preclinical or clinical evaluation (*6*, *26*–*31*). Our previous HTS work identified additional promising candidates, including MLS1082 and MLS6585 (*24*). MLS1082 was found to target the same intracellular loop 2 (ICL2) binding site as LY3154207, whereas MLS6585 appeared to interact with an unknown site on the D1R (*23*, *32*).

Despite these advances, important challenges remain in achieving precise and therapeutically effective modulation of D1R signaling. The clinical evaluation of D1R PAMs has highlighted the complexity of translating receptor potentiation into therapeutic benefit. For instance, LY3154207 (Mevidalen), the most clinically advanced D1R PAM, demonstrated dose-dependent cardiovascular effects that prevented full exploration of its therapeutic potential and failed to demonstrate efficacy in cognitive enhancement in patients with Lewy body dementia (*29*), although D1R activation is known to be procognitive (*20*). The mechanisms underlying these limitations remain unresolved and may involve suboptimal D1R potentiation, peripheral D1R activation, off-target effects, systemic dopaminergic modulation, exposure-related intolerance, disease-specific factors, or a combination thereof. More broadly, D1R PAM candidates face translational challenges, including species-specific activities, suboptimal pharmacokinetics, and difficulties in achieving sufficient therapeutic efficacy without dose-limiting side effects (*6*, *29*, *33*). These observations indicate the need to better understand the molecular principles governing D1R allosteric regulation and to explore strategies that enable more precise control of receptor activation.

Previous pharmacological studies have suggested that chemically distinct D1R PAMs—including MLS6585, BMS-A1, and LY3154207—may engage distinct allosteric sites and exhibit cooperative effects when coadministered (*33*). These findings raise the intriguing possibility that D1R function is governed by an interconnected allosteric landscape, in which coordinated engagement of multiple allosteric sites may provide an additional level of control over D1R activation beyond single-site modulation. However, the molecular basis underlying this allosteric cooperativity remains poorly understood, with only the binding site of LY3154207 definitively characterized to date (*34*). To address these critical gaps and establish a structural and mechanistic framework for cooperative allosteric modulation of the D1R, we first optimized MLS6585 through medicinal chemistry, yielding two analogs, UNC10062 and UNC9815, with enhanced potency and efficacy (*35*). By combining pharmacological characterization with cryo–electron microscopy (cryo-EM) structural analyses, we identified two previously unidentified allosteric sites on the D1R that selectively interact with either BMS-A1 or the UNC compounds. Molecular dynamics (MD) simulations further uncovered mechanisms of synergistic allosteric regulation when multiple (two or three) PAMs occupy the receptor. Our findings reveal a previously unrecognized mode of cooperative GPCR allostery and provide a structural foundation for rational exploration of multisite receptor modulation.

## RESULTS

### Mutual cooperativity of three distinct D1R PAM scaffolds

We previously described the D1R-G_s_ complex structures bound to the orthosteric agonists DA or SKF81297 together with the D1R PAM LY3154207 (*34*). We had also attempted to obtain a D1R structure using these orthosteric agonists and the D1R PAM MLS6585, but the MLS6585 binding density was not detectable, potentially due to its low binding affinity (*24*, *34*). In a separate line of investigation (to be described elsewhere), we performed the chemical optimization of MLS6585 to increase its potency and efficacy for potentiating DA affinity and signaling at the D1R, yielding optimized analogs UNC9815 and UNC10062 (Fig. 1A and fig. S1). We then used UNC9815 and UNC10062 to investigate the cooperative effects with LY3154207 and BMS-A1 using D1R-mediated β-arrestin recruitment assays.

**Fig. 1.**
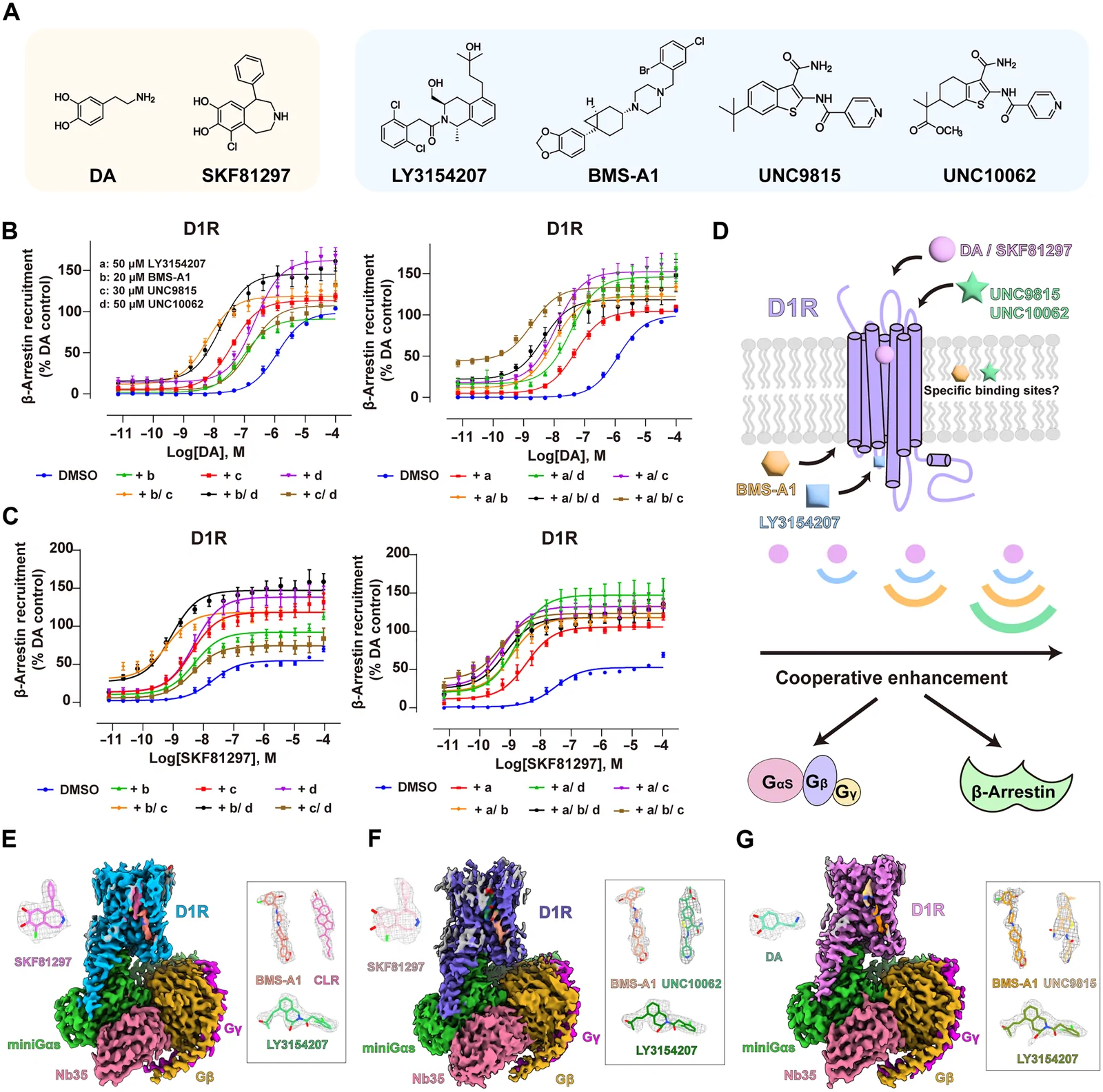
Cooperative pharmacology of D1R PAMs and cryo-EM structures of three D1R signaling complexes. (**A**) Chemical structures of the D1R orthosteric agonists DA and SKF81297 (pale yellow), and the PAMs LY3154207, BMS-A1, UNC9815, and UNC10062 (pale blue) (**B** and **C**) Cooperative enhancement of D1R activation by LY3154207, BMS-A1, and UNC compounds (UNC9815 or UNC10062) in the presence of DA (B) or SKF81297 (C), measured by β-arrestin recruitment using the DiscoverX assay. Responses are normalized to the maximal DA response in each experiment and shown as means ± SEM from at least six independent experiments, each performed in triplicate. Curve-fitting parameters and statistical analyses are provided in table S2. (**D**) Conceptual schematic illustrating cooperative enhancement of D1R signaling by orthosteric agonists and multiple PAMs. While the binding sites for DA/SKF81297 and LY3154207 were established, the binding pockets for BMS-A1 and UNC9815/UNC10062 remained undefined at this stage, motivating the structural studies shown in (E) to (G) to determine whether these PAMs occupy distinct allosteric sites that collectively enhance G_s_- and β-arrestin–mediated signaling. (**E** to **G**) Cryo-EM density maps of three D1R-miniG_s_ signaling complexes bound to orthosteric agonists and distinct combinations of PAMs: SKF81297–LY3154207–BMS-A1 (E), SKF81297–LY3154207–BMS-A1–UNC10062 (F), and DA–LY3154207–BMS-A1–UNC9815 (G). Ligand densities are shown at contour levels of 0.43 in (E), 0.26 in (F), and 0.30 in (G); the BMS-A1 density in (G) is additionally displayed at 0.07 to visualize weaker map features.

Initially, we performed curve-shift assays to establish a maximally effective dose of each PAM for increasing the potency of DA for stimulating β-arrestin recruitment to the D1R. As shown in fig. S2 and table S1, each PAM dose dependently increased the functional potency of DA [as reflected by decrease in EC_50_ (median effective concentration)] in a saturable manner, while their effects on DA efficacy (*E*_max_) were more modest, although the UNC compounds appeared to produce larger increases in *E*_max_ than BMS-A1 (fig. S2). Similar differences were also observed with SKF81297, where the UNC compounds produced substantially greater enhancement of maximal β-arrestin recruitment than BMS-A1, suggesting that chemically distinct PAM scaffolds may differentially influence receptor signaling (Fig. 1, B and C, and table S2). PAMs can promote receptor signaling by increasing agonist potency (EC_50_), increasing agonist efficacy (*E*_max_), producing direct allosteric agonism, or through a combination of these effects. Notably, the effects of PAMs on GPCR signaling can be assay-dependent (*14*). Thus, for consistency and comparison across multiple assays in this study, we used the PAM-induced increase in agonist potency (EC_50_) as the primary metric for quantifying PAM activity while also reporting effects on *E*_max_ and allosteric agonism. Using the EC_50_ fold-shift results in fig. S2 and table S1, the D1R PAMs exhibited the following rank order of activity: LY3154207 ∼ UNC9815 > BMS-A1 > UNC10062.

Having established maximally effective concentrations for each D1R PAM, we performed additivity experiments and found that combinations of these PAMs synergistically increased the functional potencies of both DA and SKF81297 in the β-arrestin recruitment assay. When all three chemically distinct classes of PAMs were combined with DA, we observed a ≥1000-fold shift in the DA EC_50_ (Fig. 1, B and C, and table S2), representing a substantial pharmacological amplification far exceeding what could be achieved by any single PAM alone. Using SKF81297, a partial agonist in this assay, the three PAMs were functionally additive with each other; however, the triple PAM combination did not potentiate the SKF81297 EC_50_ beyond that observed with any double PAM combination (Fig. 1, B and C, and table S2). This observation suggests a ceiling effect such that the D1R is maximally responsive to SKF81297 when bound to at least two of the PAMs. We also observed additive effects between the two or three PAM scaffolds when using a TRUPATH BRET assay that directly monitors agonist-induced activation of G_s_ signaling (fig. S3 and table S3), confirming previous observations using a cAMP assay (*33*). Together, these findings reveal pronounced functional cooperativity among multiple D1R allosteric sites, and, importantly, that three distinct allosteric binding sites must exist on the D1R, which might be leveraged to synergistically enhance receptor function (Fig. 1D).

### Binding modes of divergent PAM combinations in D1R

To comprehensively elucidate the molecular interactions and mechanisms of these allosteric modulators—including BMS-A1, UNC9815, and UNC10062—we determined the structures of D1R-G_s_ signaling complexes bound to these PAMs. Using the potent agonist SKF81297 to occupy and stabilize the OBP of D1R (*34*, *36*), we focused first on determining the structure of the D1R in complex with BMS-A1 alone or BMS-A1 and UNC10062 together. As our functional assays demonstrated that SKF81297 is a partial agonist compared to DA in the β-arrestin recruitment assay (Fig. 1, B and C, and table S2), we also used DA to occupy the OBP to investigate the PAM effects on full agonist binding with cryo-EM analysis. Notably, since LY3154207 binds to a distinct site from these PAMs and exerts synergistic PAM effects (*33*), to improve stabilization and conformational homogeneity of the active D1R-G_s_ complex during purification and structure determination, we incorporated LY3154207 in all sample preparations.

The cryo-EM structures of D1R-G_s_ in complex with SKF81297/LY3154207/BMS-A1, SKF81297/LY3154207/BMS-A1/UNC10062, and DA/LY3154207/BMS-A1/UNC9815 were determined at global resolutions of 2.65, 2.59, and 2.44 Å, respectively (fig. S4 and table S4). The high-resolution maps allowed us to unambiguously model most regions of the D1R-G_s_ complex, the orthosteric agonists SKF81297 or DA, and PAMs (Fig. 1, E to G). These structures demonstrated a previously unidentified simultaneous binding of allosteric modulators in three nonoverlapping pockets on a GPCR, providing the structural basis for multisite allosteric cooperativity. As previously reported (*34*, *37*–*39*), LY3154207 bound above ICL2 within the D1R. In the map of D1R-SKF81297/LY3154207/BMS-A1, we observed the robust density of BMS-A1 at the intracellular side of the receptor, with BMS-A1 sandwiched between TM1 and TM7 in a vertical conformation above Helix 8 (H8) (Figs. 1E and 2A), a positive allosteric site analogous to those identified in the D2R (*40*), A_1_ adenosine receptor (A_1_AR) (*41*), A_3_AR (*42*), and free fatty acid receptor 2 (FFAR2) (*43*). While BMS-A1 density was confirmed in the other two maps, D1R-SKF81297/LY3154207/BMS-A1/UNC10062 and D1R-DA/LY3154207/BMS-A1/UNC9815, we also observed distinct densities above BMS-A1 at the extracellular portion of the interface between TM1 and TM7, attributable to the UNC compounds in vertical orientations in the corresponding maps (Figs. 1, F and G and 2A). These two previously uncharacterized allosteric sites—the upper and lower pockets at the TM1 to TM7 interface—are organized in a spatially complementary manner as vertically adjacent binding pockets. UNC9815 and UNC10062 share high structural similarity and bind to the same pocket with comparable orientations. The stacked arrangement of two PAMs sandwiched between TM1 and TM7 represents, to our knowledge, a previously unknown allosteric binding configuration in other GPCRs. In the map of D1R-SKF81297/LY3154207/BMS-A1, we observed a clear density of cholesterol at a site similar to that occupied by the UNC compounds (Fig. 1E and fig. S5A). Lipids are also observed at the extracellular TM1 to TM7 interface in multiple independent D1R structures (*34*, *36*–*38*, *44*), suggesting that the upper pocket represents a preexisting lipid-accessible cavity (fig. S5A). This intriguing observation suggests that the UNC compounds and cholesterol may play similar roles in allosterically modulating D1R function.

**Fig. 2.**
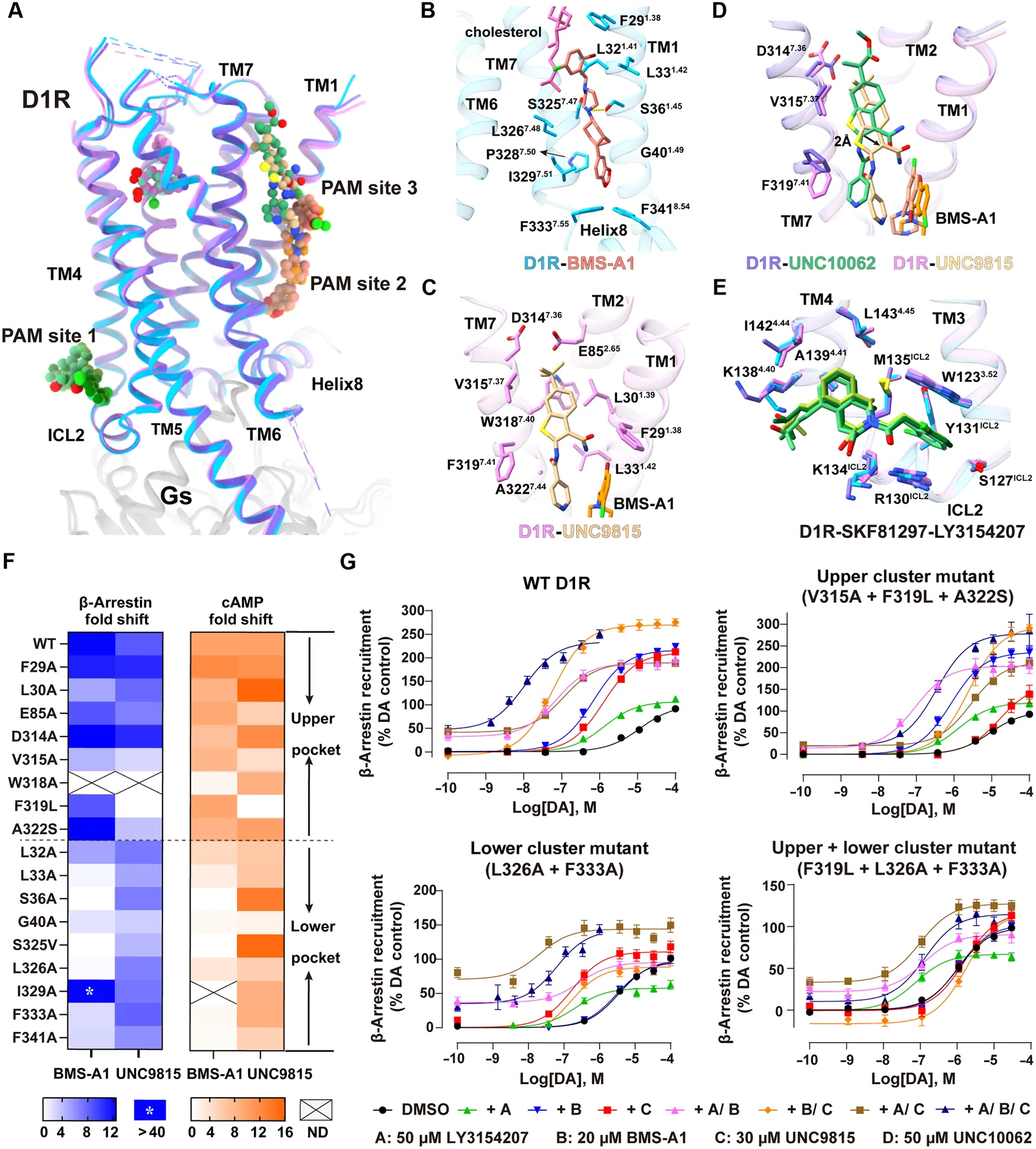
Structural and functional definition of three distinct allosteric sites in D1R. (**A**) Structural alignment of three D1R–G_s_ complexes reveals three nonoverlapping PAM-binding sites. LY3154207 binds at the intracellular ICL2-facing site (PAM site 1), BMS-A1 occupies an extrahelical pocket at the lower TM1 to TM7 interface (PAM site 2), and UNC10062/UNC9815 bind in an upper pocket directly above BMS-A1 (PAM site 3). (**B**) Binding pose and interaction network of BMS-A1 at PAM site 2 in the SKF81297–LY3154207–BMS-A1-bound D1R-G_s_ complex. (**C**) Binding pose and key interactions of UNC9815 at PAM site 3 in the DA–LY3154207–BMS-A1–UNC9815-bound D1R-G_s_ complex. (**D**) Superposition of UNC10062 and UNC9815 showing distinct binding geometries at PAM site 3; UNC9815 extends ∼2 Å deeper toward the intracellular side than UNC10062, measured from the carbamoyl carbon position. (**E**) Structural alignment of LY3154207 in the three complexes showing a conserved binding mode at PAM site 1. (**F**) Heatmap summarizing the effects of indicated D1R mutations on BMS-A1- and UNC9815-induced potentiation in β-arrestin and cAMP BRET assays, expressed as EC_50_ fold shifts relative to WT D1R. Concentration-response curves are shown in figs. S6 to S9, and fitted parameters are provided in tables S5 to S8. ND, EC_50_ not determinable. *, fold shift > 40. (**G**) Functional cooperativity of PAMs with mutations in the upper and lower TM1 to TM7 allosteric pockets. DA-stimulated β-arrestin recruitment was measured by CAAX BRET for WT and mutant D1Rs in the absence or presence of the indicated PAMs. Responses are normalized to the maximal control DA response. Representative curves from ≥6 independent experiments performed in duplicate are shown; mean fitted parameters and statistics are provided in table S9.

### Recognition of PAMs in divergent allosteric sites of D1R

The TM1 to TM7 and both UNC compounds are predominantly characterized by hydrophobic, aromatic, and van der Waals interactions (Fig. 2, B and C, and fig. S5B). In our structures, BMS-A1 occupies a large vertically extended pocket spanning from the middle to the intracellular end of TM7 and above H8 (referred to as the “lower pocket”). The lower pocket is mainly formed by residues from TM1, TM7, and H8, including F29^1.38^, L32^1.41^, L33^1.42^, S36^1.45^, G40^1.49^, S325^7.47^, L326^7.48^, I329^7.51^, F333^7.55^, and F341^8.54^ (Fig. 2B). Specifically, the benzodioxole moiety of BMS-A1 points to H8 and interacts with F333^7.55^ and F341^8.54^, while the 2-bromo-5-chlorobenzyl group stacks with F29^1.38^, L32^1.41^, and L33^1.42^ (Fig. 2B). In addition to hydrophobic interactions, the piperazine ring of BMS-A1 forms a polar hydrogen-bond interaction with S36^1.45^ (Fig. 2B).

The binding poses of both LY3154207 and BMS-A1 remain largely preserved following the addition of the UNC compounds (Fig. 2, D and E). However, the 2-bromo-5-chlorobenzyl group of BMS-A1 undergoes notable conformational changes due to specific interactions with these UNC compounds (Fig. 2D and fig. S5C). While both UNC compounds occupy the same allosteric site located in the extracellular cleft between TM1 and TM7 (referred to as the “upper pocket”), positioned directly above the BMS-A1 binding pocket, they exhibit distinct binding modes (Fig. 2, C and D, and fig. S5B). Specifically, UNC9815 penetrates ∼2 Å deeper toward the intracellular side compared to UNC10062 when measured at the α-carbon atoms of their respective carbamoyl groups (Fig. 2D). This difference in positioning may be attributed to the more rigid planar benzothiophene core structure of UNC9815 compared to the relatively flexible half-boat conformation of the tetrahydrobenzothiophene moiety of UNC10062 (Figs. 1A and 2C, and fig. S5B). Unlike the BMS-A1/UNC10062-bound D1R, little density of the 2-bromo-5-chlorobenzyl group of BMS-A1 could be observed in the BMS-A1/UNC9815–bound structures due to the conformational flexibility of this group (Fig. 1, F and G). The hydrophobic pocket to which both UNC10062 and UNC9815 vertically bind is mainly constituted by F29^1.38^, L30^1.39^, L33^1.42^, E85^2.65^, D314^7.36^, V315^7.37^, W318^7.40^, F319^7.41^, and A322^7.44^ (Fig. 2C and fig. S5B).

To confirm the above structural findings, we performed extensive mutagenesis experiments targeting the PAM contact residues within the D1R TM1-TM7 upper and lower allosteric pockets. Initially, we generated single-residue mutants and evaluated their effects on BMS-A1 and UNC9815 PAM activities using DA as the orthosteric agonist. We chose to evaluate UNC9815 as the representative UNC D1R PAM because it is more efficacious than UNC10062 in potentiating the potency of DA (fig. S2 and table S1). In addition, we evaluated the D1R point mutants using both β-arrestin recruitment and cAMP accumulation assays (Fig. 2F, figs. S6 to S10, and tables S5 to S8). Consistent with the structural findings, the mutation of most of the surrounding residues of the upper pocket, particularly V315A, F319L, and A322S, attenuated the DA EC_50_ fold-shift observed with UNC9815 (Fig. 2F; figs. S6, S8, and S10; tables S5 and S7). In contrast, the mutation of just one lower binding pocket residue, G40A, affected UNC9815 activity, which was observed in both functional assays (Fig. 2F and fig. S10). The conserved G40^1.49^ residue had been found to introduce flexibility and bending into TM1 (*45*), leading to a distinct orientation of its extracellular portion compared with receptors containing P39^1.48^. Thus, the G40A mutation at the intracellular side of TM1 is expected to propagate structural effects to the extracellular end of the helix.

The mutation of most of the lower pocket residues—including L32A, L33A, S36A, G40A, S325V, L326A, I329A, F333A, and F341A—significantly affected the PAM activity of BMS-A1 in one or both D1R functional assays (Fig. 2F; figs. S7, S9, and S10; tables S6 and S8). In most cases, these mutations attenuated the BMS-A1 PAM activity; however, in the β-arrestin recruitment assay, the I329A mutation produced a significant gain of BMS-A1 PAM efficacy (Fig. 2F, fig. S10, and table S6). Only one upper pocket residue, W318A, attenuated BMS-A1 activity and only in the cAMP assay (figs. S9 and S10). Together, these experiments strongly support the poses of UNC9815 and BMS-A1 in the upper and lower pockets, respectively, as observed in the cryo-EM experiments.

To further validate the PAM binding poses revealed by the cryo-EM structures, we created D1R cluster mutants of either the upper or lower pockets or both. For these mutants, we chose residues that, when mutated, had minimal effects on DA potency yet had significant effects on PAM activity, as observed in the single mutant experiments. Consistent with the point mutation studies, we used UNC9815 as the representative UNC compound in β-arrestin recruitment assay. As shown in Fig. 2G and table S9, when using the wild-type (WT) D1R, dual and triple combinations of UNC9815, BMS-A1, and LY3154207 exhibited additive effects on potentiating the potency of DA, resulting in a 1500-fold leftward shift in the DA EC_50_ with all three PAMs combined. In the D1R upper cluster mutant, consisting of V315A-F319L-A322S, BMS-A1, and LY3154207 produced similar results as seen with the WT receptor (including in combination). However, UNC9815 unexpectedly behaved as a weak negative allosteric modulator (NAM) when tested alone or in combination with BMS-A1 or LY3154207 (Fig. 2G and table S9). In all cases, UNC9815 significantly shifted DA’s EC_50_ to lower potency compared to that seen in the corresponding controls. These results suggest that UNC9815 still interacts with the receptor with the cluster mutations in the upper pocket; however, these mutated residues have altered the allosteric efficacy of this modulator converting it from a PAM to a NAM. In contrast, in the lower cluster D1R mutant, consisting of L326A-F333A, the PAM activity of UNC9815 was retained and that of LY3154207; however, BMS-A1 was rendered functionally inactive for altering DA potency either alone or in combination with UNC9815 or LY3154207 (Fig. 2G and table S9). Similarly, BMS-A1 was inactive in the D1R upper/lower cluster mutant (D1R- F319L-L326A-F333A), whereas the PAM activity of LY3154207 was retained. In contrast, UNC9815 exhibited weak, but significant, NAM activity when tested with DA alone but was functionally inactive when tested in combination with BMS-A1 and/or LY3154207 (Fig. 2G and table S9). Together, these results confirm the receptor interaction sites of all three PAM scaffolds and serve to illustrate the allosteric flexibility of the D1R.

### Stable occupation of BMS-A1 and UNC compounds in the D1R

To assess the stability and conformational flexibility of BMS-A1, UNC9815, and UNC10062 binding modes observed in the cryo-EM structures of D1R, we performed MD simulations on all three experimentally determined complexes (table S10). Unlike the cryo-EM study, which was conducted in detergent micelles, our simulations embedded D1R in an explicit 1-palmitoyl-2-oleoyl-*sn*-glycero-3-phosphocholine (POPC) bilayer, providing a complementary view of receptor-ligand dynamics in a more physiologically relevant lipid environment. BMS-A1 contains a piperazine ring that forms a polar hydrogen bond with S36^1.45^ in the cryo-EM structure and may carry a positive charge depending on its local environment, suggesting that protonation state could influence binding. However, predicting the p*K*_a_ (where *K*_a_ is the acid dissociation constant) of ligands in a membrane environment remains challenging, as most algorithms rely on empirical models trained on aqueous systems and lack accuracy in lipid environments. Using Epik (Schrödinger), we predicted the protonated form of BMS-A1 to be energetically favored over the neutral form, albeit with a modest state penalty of 1.2 kcal/mol (within the typical error range of such calculations). To evaluate the impact of the protonation state on binding stability, we performed MD simulations of D1R with both the charged and neutral forms of BMS-A1 (table S10).

The analysis of the MD simulations, performed in the presence of LY3154207 unless otherwise noted, revealed that, contrary to the initial Epik prediction, the neutral form of BMS-A1 maintained a vertically extended pose within the lower pocket (fig. S11). In contrast, the charged form exhibited reduced stability, frequently adopting flipped conformations or dissociating from the pocket. These results suggest that the neutral state of BMS-A1 forms a more stable interaction at the lower pocket in the membrane-embedded receptor. For the bound UNC compounds, we observed subtle deviations from their cryo-EM binding poses, likely due to the distinct simulation environment; however, they largely retained their overall orientations at the upper site throughout most simulations (fig. S11). To assess the stability of the equilibrated binding modes of BMS-A1 and the UNC compounds during simulations, we calculated pairwise ligand root mean square deviation (RMSD). Both compounds consistently exhibited low RMSD values, indicating stable binding within their respective pockets between TM1 and TM7 (fig. S11). UNC9815, lacking the polar methylpropanoate group, displayed a tendency to shift between two positions within the predominantly hydrophobic upper pocket. This movement did not affect the stable binding of BMS-A1 in the lower pocket (fig. S11). These results suggest that the membrane environment could preserve the PAM poses observed in the cryo-EM structures.

The comparison of the binding poses of BMS-A1 and UNC compounds in the absence of each other showed that both PAMs maintained stable interactions within their respective allosteric pockets. BMS-A1 remained stably bound in the lower pocket across all simulations, regardless of UNC compound occupancy in the upper site, as indicated by low ligand RMSD values and consistent interaction profiles. However, some conformational flexibility was observed in the 2-bromo-5-chlorobenzyl moiety of BMS-A1, in the absence of the bound UNC compounds, consistent with the weaker cryo-EM density for this group in the cryo-EM structures and a slight shift in pairwise RMSD (fig. S11). Similarly, the UNC compounds maintained a stable pose in the upper pocket, closely aligned with their experimental conformations, in the absence of BMS-A1. These results indicated that, although cobinding may influence local pocket organization, each PAM was independently capable of adopting a stable binding mode within its respective site.

To determine whether LY3154207 is required for stable occupancy of the TM1 to TM7 allosteric pockets, we performed additional MD simulations of D1R-G_s_ complexes containing BMS-A1, UNC10062, or UNC9815 in the absence of LY3154207. In all three systems, the PAMs remained bound within their respective pockets throughout the simulations. Compared with the LY3154207-containing systems, the ligand pairwise RMSD distributions were broader, indicating increased local conformational fluctuations in the absence of LY3154207 (fig. S12). These results suggest that LY3154207 enhances the overall stability of the receptor-ligand complexes but is not required for BMS-A1 binding to the lower pocket or UNC10062/UNC9815 binding to the upper pocket at the TM1 to TM7 interface.

### Selective determinants of BMS-A1 and UNC compounds to DRs

We have previously determined the active-state structures of all DRs (*44*). The comparison of these structures with the new D1R structures bound to BMS-A1 and UNC compounds allowed us to identify the molecular determinants for receptor subtype selectivity of these PAMs. Within the DR family, the D1R exhibits the highest sequence and structural homology with D5R. Despite considerable conservation in TM1 and TM7 between D1R and D5R (Fig. 3A), a notable substitution is the replacement of S36^1.45^ in D1R by W53^1.45^ in D5R. This substitution eliminates the essential hydroxyl group required for optimal BMS-A1 binding and introduces steric hindrance that impedes BMS-A1 interaction (Fig. 3B), accounting for the selectivity of BMS-A1 to D1R when compared to D5R (*33*). Consistent with these structural insights, we observed that introducing an S36W mutation in the D1R diminished BMS-A1 PAM activity while making the reciprocal W53S mutation in D5R engendered BMS-A1 PAM activity (Fig. 3, C and D, and table S11). The upper pocket around TM1 and TM7 in D5R remains largely conserved relative to D1R, except for F29^1.38^ in D1R being replaced by L46^1.38^ in D5R (Fig. 3, E and F). Consistent with these observations, we previously demonstrated that MLS6585, the parent compound of UNC10062 and UNC9815, potentiates dopamine-stimulated β-arrestin recruitment to the D5R (*24*), aligning with our current findings.

**Fig. 3.**
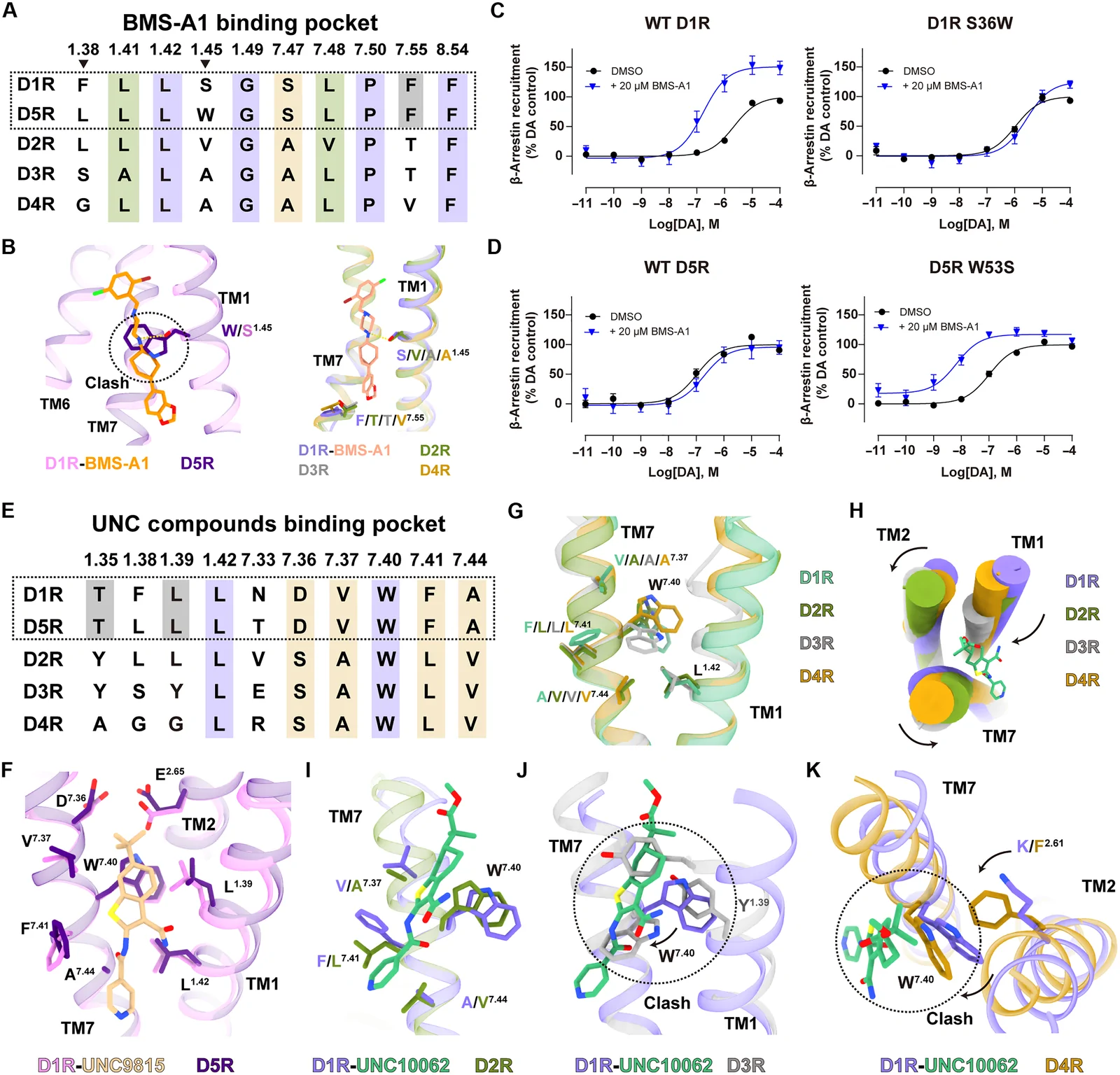
Structural basis for subtype selectivity of BMS-A1 and UNC PAMs. (**A** and **E**) Sequence alignment of DR subtypes highlighting residues that form the BMS-A1 pocket (A) or the UNC-compound pocket (E), with subtype-divergent positions indicated. (**B**) Structural comparison of BMS-A1-bound D1R with rotigotine-bound D5R and D2-like receptors. In D5R, substitution of D1R S36^1.45^ by W53^1.45^ creates a steric clash that disfavors BMS-A1 binding (left). Comparison with D2-like receptors further reveals nonconserved residues at positions 1.45 and 7.55 that contribute to subtype selectivity (right). (**C** and **D**) Reciprocal substitutions at position 1.45 alter BMS-A1 activity. DA concentration–response curves were measured in the absence or presence of 20 μM BMS-A1 using β-arrestin recruitment BRET assays and are expressed as a percentage of the maximal control DA response (mean ± SEM, *n* = 4). (C) D1R WT and S36W. (D) D5R WT and W53S. Fitted parameters and statistical analyses are provided in table S11. (**F**) Superposition of UNC9815-bound D1R with rotigotine-bound D5R showing conservation of the upper PAM pocket within D1-like receptors. (**G**) Structural alignment of UNC-bound D1R with D2-like receptors at PAM site 3 highlighting subtype-specific pocket differences. (**H**) Structural comparison of UNC10062-bound D1R with D2-like receptors reveals helical displacement and altered pocket topology. (**I** to **K**) Comparisons of the UNC10062-bound D1R structure with D2R (I), D3R (J), and D4R (K) reveal altered helical positions and pocket topologies that are incompatible with UNC binding. In D3R and D4R, receptor-specific side-chain rearrangements reposition W7.40 and generate steric clashes with UNC10062. Protein Data Bank structures used for comparison: rotigotine-bound D1R (8IRR), D2R (8IRS), D3R (8IRT), D4R (8IRU), and D5R (8IRV).

In D2-like receptors, the residue composition of both BMS-A1 and UNC compound binding sites differs significantly from D1R, with key amino acid substitutions that create unfavorable binding conditions in D2-like receptors (Fig. 3, A and B, E, and G and H). In addition, the conformation of the conserved W^7.40^ in D3R and D4R is rearranged by surrounding residues with bulky side chains, with its indole ring potentially introducing substantial steric constraints against UNC compounds (Fig. 3, G to K). These structural differences collectively explain the selectivity of these compounds for D1R over D2-like receptors (*24*).

### Positive allosteric mechanisms of BMS-A1 and UNC compounds

Previous studies have demonstrated that LY3154207 occupies an allosteric site directly above the ICL2 in a boat-shaped configuration (*34*, *37*–*39*). It exerts PAM activity through stabilization of the helical conformation of ICL2, which is then propagated to affect the conformation of the TM domain (*39*). BMS-A1 and the UNC compounds, however, bind two other distinct allosteric sites, suggesting divergent mechanisms of allosteric modulation.

We recently determined the structure of D1R bound to the inverse agonist LE300 in a companion study (*46*). To characterize conformational changes associated with receptor activation and positive allosteric modulation, we used the protein interaction analyzer (PIA) (*47*) to examine three functional states of D1R: the inactive state (LE300 bound), the active state (agonist bound), and the super-stabilized active state (agonist-triple-PAM bound). In this framework, the agonist-bound receptor represents the canonical active state relative to the inactive conformation. The additional binding of three D1R PAMs stabilizes a more fully organized active-state ensemble, which we refer to as the super-stabilized active state. The comparison of inactive and active states revealed hallmark activation changes, including F281^6.44^ moving away from the OBP center and outward displacement of TM6i, consistent with our previous observations across aminergic GPCRs (fig. S13) (*48*). In addition, in the active state, the extracellular portion of TM6 (TM6e, residues 6.55 to 6.59) moves toward, while S188^ECL2.52^ in extracellular loop 2 (ECL2) shifts away from other binding site residues (fig. S13). PAM binding did not further displace F281^6.44^ from the OBP center but consistently induced inward movement of E85^2.65^ with either DA or SKF81297, a rearrangement likely stabilizing the super-stabilized active conformation (fig. S14A). Notably, S188^ECL2.52^ exhibited increased separation from binding site residues in the LY3154207/BMS-A1– and LY3154207/BMS-A1/UNC10062–bound states with SKF81297 but not with DA, while only in the triple PAM-bound state did TM6e move further inward (fig. S14). Thus, given that SKF81297 is a partial agonist, these ECL2 and TM6e motions proceed in consistent directions from inactive to increasingly active states.

Comparison between the inactive and SKF81297-bound active state also revealed inward movement of TM7i, which facilitates the outward tilting of TM6i, leading to opening of the G protein–binding cavity on the intracellular side of the TM domain, critical steps in signaling (*49*, *50*). Upon the binding of the BMS-A1 and UNC compounds, we observed a more substantial inward movement of TM7i, accompanied by notable conformational rearrangements of the conserved NPxxY and Y^5.58^-Y^7.53^ motifs (Fig. 4A). The polar interaction between residues T^1.46^ and S^7.47^ appears to play a crucial role in maintaining the inactive state of D1R, as demonstrated in our companion study (*46*), but is disrupted upon activation, driving outward movement of TM1i. BMS-A1 binding further amplifies this disruption, inducing greater outward displacement of TM1i (Fig. 4B). Notably, LY3154207 binding alone does not produce additional TM1i movement beyond the SKF81297-bound state, indicating that LY3154207 and BMS-A1 exert distinct allosteric effects on D1R. Furthermore, the binding of UNC compounds to the upper allosteric site potentially stabilizes the TM7 extracellular region in its active conformation (fig. S15). Together, these mechanisms may underlie the synergistic increase in PAM activity observed with different combinations of LY3154207, BMS-A1, and UNC compounds.

**Fig. 4.**
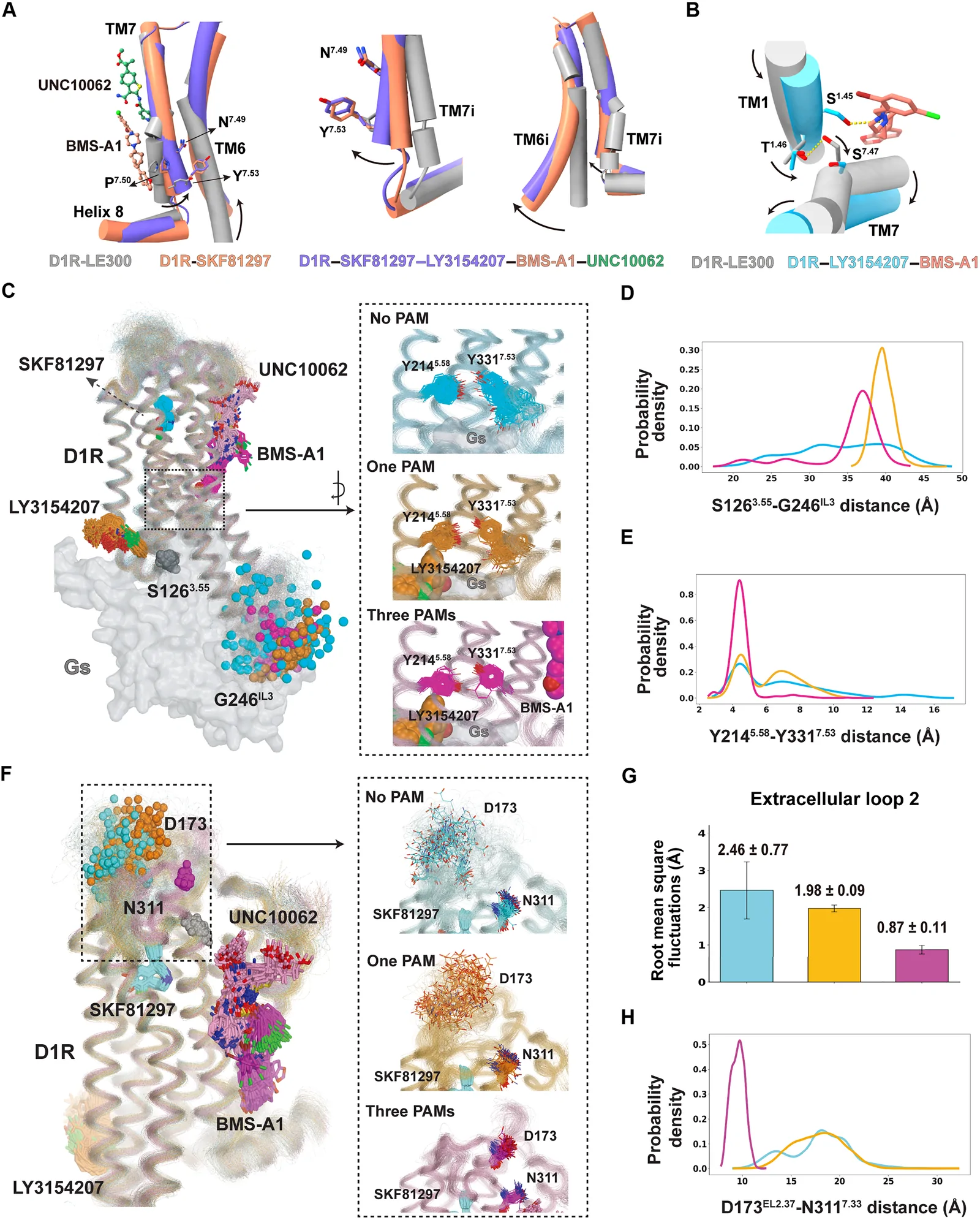
PAM-induced conformational stabilization in D1R. (**A**) Structural alignment of inactive, active (agonist-bound), and super-stabilized active (agonist-triple-PAM–bound) D1R states showing progressive inward movement of TM7i and outward displacement of TM6i with increasing receptor activation. (**B**) Extracellular top view showing disruption of the T^1.46^–S^7.47^ hydrogen bond upon BMS-A1 binding. (**C** and **F**) Representative MD snapshots of D1R under three conditions, shown without G protein: D1R-SKF81297 (no PAM, cyan), D1R-SKF81297-LY3154207 (one PAM, orange), and D1R–SKF81297–LY3154207–BMS-A1–UNC10062 (three PAMs, magenta). (C) ICL3, monitored by the Cα position of G246, is more flexible in the absence of PAMs and frequently samples the region occupied by G protein (gray surface), whereas Y331^7.53^ becomes increasingly confined in the triple-PAM–bound state, consistent with local stabilization near the lower allosteric pocket. (F) PAM binding progressively stabilizes the relative positions of D173^ECL2.37^ and N311^7.33^. (**D**, **E**, and **H**) Kernel density distributions of inter-residue distances for the three simulation conditions in (C) and (F). PAM binding progressively narrows the distance distributions for S126^3.55^ Cα–G246I^CL3^ Cα (D), the hydroxyl O─O distance between Y331^7.53^ and Y214^5.58^ (E), and D173^ECL2.37^-N311^7.33^ (H) indicating coordinated stabilization of extracellular and intracellular receptor elements. (**G**) Root mean square fluctuation analysis showing progressive reduction in ECL2 mobility with increasing PAM occupancy.

The allosteric effects of these small-molecule PAMs may be obscured by the dominant influence of G protein interaction on receptor conformation. To isolate and evaluate the specific effects of the BMS-A1 and UNC compounds from those of LY3154207 on D1R conformational dynamics in the absence of G protein, we performed MD simulations on three systems: D1R-SKF81297-only, D1R-SKF81297/LY3154207, and D1R-SKF81297/LY3154207/BMS-A1/UNC10062 (triple-PAM), after removing the G protein from the cryo-EM complex structure (table S10). In all simulations, the ligands remained stably bound to their respective sites and preserved their cryo-EM poses (Fig. 4, C and F), consistent with their behavior in G protein–coupled simulations.

In the absence of G protein, we observed notable differences in local flexibility between PAM-bound and unbound systems. Multisite PAM engagement produced stabilizing effects not achieved by LY3154207 alone. The ICL3 was substantially more dynamic without PAMs (Fig. 4, D and E). By tracking the Cα atom of G246^ICL3^, a representative ICL3 residue, we found that in the SKF81297-only condition, G246^ICL3^ sampled a broader space, frequently sampling conformations extending into the region that would be occupied by a bound G protein (Fig. 4, C and D). In contrast, the triple PAM-bound system showed a more compact distribution, suggesting that PAM binding stabilized ICL3 in a more open orientation even without the G protein. Moreover, our simulations indicated that the binding of BMS-A1 and UNC10062 stabilized the NPxxY motif in its active conformation (Fig. 4, C and E). Specifically, Y331^7.53^, a key residue within this conserved motif, remained highly flexible in the D1R-SKF81297-only and D1R-SKF81297/LY3154207 simulations but adopted a more stable conformation in the triple-PAM system, resembling that observed in the G protein–bound state (Fig. 4, C and E). These findings support that BMS-A1 and UNC10062 promote a more ordered, potentially super-stabilized active receptor state by stabilizing TM7i and its associated microswitch motifs and, more broadly, by conferring structural rigidity to the intracellular regions of D1R in the absence of the G protein, thereby biasing the receptor toward an active conformation.

Beyond these intracellular effects, multisite PAM engagement also stabilized the extracellular loops of D1R. Root mean square fluctuation analyses revealed a progressive reduction in ECL2 flexibility: from 2.46 ± 0.77 Å in the D1R-SKF81297-only system to 1.98 ± 0.09 Å in the D1R-SKF81297/LY3154207 system and 0.87 ± 0.11 Å in the triple PAM-bound system (Fig. 4, F and G). This extracellular loop stabilization facilitated tightening of the loop network at the TM7-ECL3 junction, where the D173^ECL2.37^-N311^7.33^ distance shifted from a broad distribution (centered around 18 to 20 Å) in the SKF81297-only and LY3154207-bound systems to a narrower, more compact distribution (∼10 Å) in the triple PAM-bound system (Fig. 4H). This stabilization of the extracellular vestibule may restrict dissociation of orthosteric ligands, thereby contributing to the observed enhancement of agonist potency.

## DISCUSSION

The allosteric modulation of GPCRs offers a promising avenue for developing safer and more effective treatments due to superior pharmacological profiles, such as enhanced receptor selectivity and finely tuned pharmacological effects, compared to orthosteric ligands (*6*). However, the translation of allosteric modulators into effective clinical therapeutics remains challenging, as evidenced by the D1R PAM, LY3154207, thus posing an urgent need to understand fundamental mechanisms governing allosteric regulation of GPCRs (*29*). Increasing evidence indicates that many GPCRs have multiple allosteric binding sites. While single-site allosteric mechanisms have been extensively studied (*6*, *34*, *39*, *43*, *51*), whether and how multiple allosteric modulators cooperatively regulate GPCR function remains largely unexplored. The D1R has been proposed to harbor three spatially distinct PAM binding sites that function cooperatively (*33*), providing an ideal system to investigate multisite allosteric modulation. Here, we provide the first comprehensive molecular elucidation of cooperative multisite allostery in GPCRs. Combining medicinal chemistry, functional assays, cryo-EM analysis, and MD simulations, we show that allosteric modulators can synergize to enhance D1R activation acting at three spatially distinct sites: the ICL2 site occupied by LY3154207, the lower TM1 to TM7 pocket occupied by BMS-A1, and the upper TM1 to TM7 pocket occupied by the UNC compounds.

The vertically stacked arrangement of BMS-A1 and UNC compounds between TM1 and TM7, to our knowledge, represents a previously unreported configuration in GPCRs, highlighting how multiple modulators can coordinate to amplify signaling efficacy. Notably, the observation that cholesterol occupies a site similar to the UNC compounds suggests that these synthetic modulators may mimic lipid-based regulatory mechanisms. This observation aligns with broader evidence that cholesterol allosterically modulates various GPCRs, including the adenosine A2A receptor, oxytocin receptor, CC motif chemokine receptor 3, and D4R, indicating a potentially universal role for cholesterol in GPCR regulation (*44*, *52*–*55*).

Mechanistically, our structural analyses reveal that these allosteric modulators enhance D1R activation through coordinated yet distinct mechanisms that drive a progressive transition across three receptor states—active, stabilized active, and super-stabilized active (Fig. 5, A and B). The binding of orthosteric agonists such as DA or SKF81297 stabilizes the receptor in an active state. The subsequent engagement of the intracellular PAM LY3154207 promotes a stabilized active state by reinforcing the helical conformation of ICL2, particularly residues R130^34.52^ and W123^3.52^, with additional stabilization of ECL2 and ICL3 (*39*). Further binding of BMS-A1 and UNC-series compounds drives the receptor into a super-stabilized active state, in which both intracellular and extracellular structural networks become more rigid. BMS-A1 promotes a more pronounced inward movement of TM7i, particularly the conserved NPxxY motif, while disrupting the T^1.46^-S^7.47^ interaction that stabilizes the inactive conformation, thereby facilitating the outward kink of TM6i required for efficient G protein coupling (*56*–*58*). Complementarily, UNC compounds stabilize the extracellular region of TM7 through binding at the upper allosteric site, producing marked stabilization of extracellular loops, especially ECL2. Consistent with these structural observations, our MD simulations reveal that multisite PAM engagement restricts ICL3 dynamics and progressively stabilizes the TM7-NPxxY activation motif, with residues Y^5.58^ and Y^7.53^ becoming increasingly constrained, effects that were not achieved by LY3154207 binding alone. This cooperative stabilization biases the receptor toward a G protein–bound-like conformation even in the absence of G protein. Multisite engagement also progressively stabilized extracellular loops, which may restrict orthosteric ligand dissociation and thereby enhance agonist potency.

**Fig. 5.**
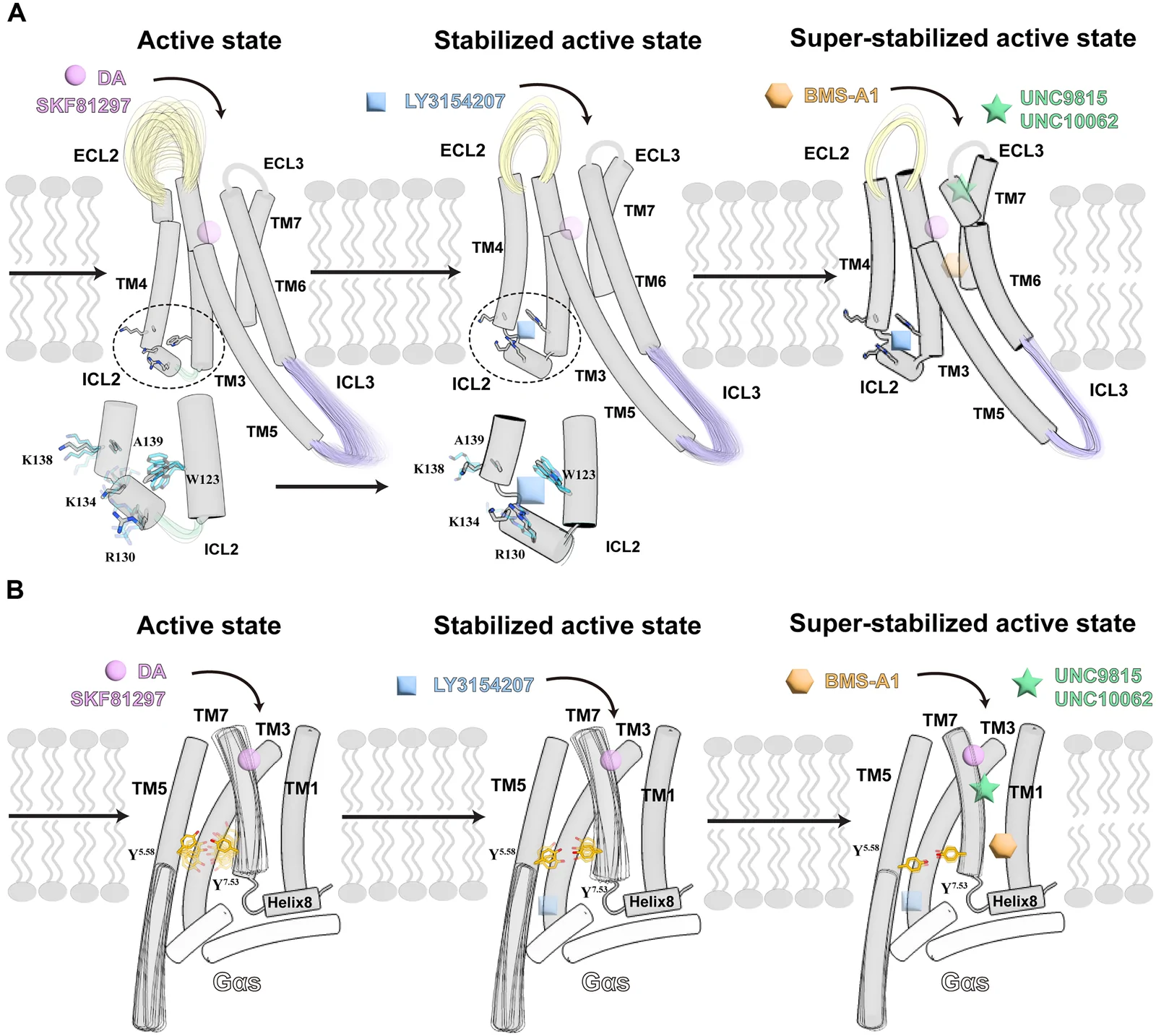
Proposed mechanism for progressive D1R activation by multisite PAM engagement. (**A**) Schematic model illustrating stepwise stabilization of D1R by orthosteric agonist and PAMs. Relative to the agonist-bound active state, binding of LY3154207 at the intracellular ICL2-facing allosteric site promotes a stabilized active state by stabilizing the helical conformation of ICL2 and the local interaction network involving W123^3.52^, R130^34.52^, K134^34.56^, K138^4.40^, and A139^4.41^. Subsequent binding of BMS-A1 in the lower TM1–TM7 pocket together with UNC9815/UNC10062 in the upper TM1 to TM7 pocket yields a super-stabilized active state, in which both extracellular (ECL2, yellow) and intracellular (ICL2, green; ICL3, purple) receptor elements are further stabilized. Dashed circles highlight progressive ordering of the ICL2 region. (**B**) Opposite view of the same model, rotated by ∼120°, highlighting PAM-dependent rearrangements in the TM5 to TM7 region. Multisite PAM engagement strengthens the Y^5.58^–Y^7.53^ (orange) interaction and stabilizes the TM7-centered NPxxY activation microswitch, consistent with cooperatively stabilized, G protein–favoring receptor conformation. Together, the model illustrates how spatially distinct PAMs act through complementary structural pathways to cooperatively amplify D1R activation.

Together, these findings define the structural basis by which coordinated engagement of distinct allosteric pockets produces effects unattainable by single-site modulators. In this model (Fig. 5), orthosteric agonists initiate receptor activation, while coordinated PAM binding progressively reinforces and amplifies this active state, forming a synergistic allosteric network that markedly amplifies receptor responsiveness. The concurrent engagement of all three allosteric sites produced pronounced functional cooperativity, increasing DA potency by >1000-fold, thereby demonstrating that coordinated multisite modulation can achieve substantial pharmacological amplification beyond that produced by individual PAMs.

An additional observation emerging from our pharmacological analyses is that the different PAM scaffolds exert distinct effects on agonist efficacy in addition to agonist potency. Whereas all three PAM classes consistently increased agonist potency, the UNC compounds produced substantially larger increases in maximal signaling than BMS-A1, particularly when the partial agonist SKF81297 was used. One possible explanation is that the UNC compounds stabilize receptor conformations that more effectively enhance agonist efficacy, whereas BMS-A1 predominantly increases agonist potency with comparatively modest effects on maximal signaling. The greater enhancement observed with SKF81297 may also reflect the increased capacity of positive allosteric modulation to augment the response of a partial agonist whose efficacy has not reached the maximal signaling capacity of the assay. However, the magnitude and direction of *E*_max_ modulation are influenced by multiple factors, including receptor reserve, signaling pathway, assay format, and agonist efficacy. Therefore, the present data do not allow us to distinguish whether these scaffold-dependent functional profiles primarily arise from differences in agonist binding cooperativity, receptor activation efficacy, transducer coupling, or combinations of these mechanisms. Addressing these questions will require future quantitative operational model analyses together with complementary kinetic, pharmacological, and structural studies.

Our analysis also elucidates the molecular basis of subtype selectivity of PAMs among DRs, a key consideration in drug development. S36^1.45^ facilitates BMS-A1 binding, whereas substitution by W53^1.45^ in D5R introduces steric hindrance and abolishes this interaction. By contrast, the conservation of the upper pocket explains why the UNC scaffold exhibits activity at D5R. In D2-like receptors, the greater divergence in both pockets underlies the selectivity of these compounds (*24*, *25*). These insights establish a foundation for rational design of subtype-selective modulators with enhanced specificity and reduced off-target effects.

Currently, only a limited number of GPCRs—including β_2_-adrenergic receptor (*59*–*61*), M_5_ muscarinic acetylcholine receptor (*62*, *63*), FFAR2 (*43*), and metabotropic glutamate (mGlu) receptors (*64*–*70*)—have been reported to possess multiple allosteric sites, although we anticipate that this will become a more widely spread phenomenon. The concept of simultaneously targeting multiple allosteric pockets within a single receptor as demonstrated in this study offers an opportunity to understand how receptor signaling can be fine-tuned through spatially distinct allosteric mechanisms. This mechanistic principle extends beyond the D1R and provides a conceptual basis for investigating cooperative multisite allostery of other GPCRs. Our findings further suggest that cooperative multisite allostery represents a mechanistically distinct receptor modulation mode that may offer opportunities for therapeutic development. However, whether this strategy can ultimately improve therapeutic efficacy, broaden therapeutic windows, or reduce adverse effects remains to be determined through future pharmacological and in vivo studies. In conclusion, our study reveals a mechanism of cooperative multisite allostery in D1R, in which distinct allosteric pockets act together to amplify receptor activation through coordinated structural stabilization. These findings expand the conceptual framework of GPCR regulation and provide a foundation for developing multisite allosteric strategies for future therapeutic discovery.

## MATERIALS AND METHODS

### Compounds

Compounds UNC9815 and UNC10062 were synthesized in-house (see Materials and Methods) at the UNC Eshelman School of Pharmacy (Chapel Hill, NC). BMS-A1 was initially provided as a gift from Eli Lilly (Indianapolis, IN); additional BMS-A1 used in this study was subsequently synthesized in-house at the UNC Eshelman School of Pharmacy by J.R., F.W., and K.J.F. (*71*). LY3154207 was purchased from MedChemExpress (Monmouth Junction, NJ). DA, SKF81297, and other fine chemicals were purchased from MilliporeSigma (St. Louis, MO) unless otherwise stated. DNA constructs for the BRET assays were gifts from J. A. Javitch and M. Bouvier. Receptor mutants were prepared at Bioinnovatise (Rockville, MD, USA), and all mutations were verified by DNA sequencing. Cell culture flasks, materials, and all assay plates were purchased from Thermo Fisher Scientific (Waltham, MA) and Greiner Bio-One (Monroe, NC). Tissue culture media and related supplies were obtained from Invitrogen (Carlsbad, CA, USA) and Corning (Glendale, AZ, USA) unless stated otherwise.

### Construct design

The full-length gene coding sequence of human D1R was synthesized with no additional mutations or loop deletions added into the gene sequence. The N-terminal of D1R sequence was induced with FLAG tag followed by a fragment of β_2_AR N-terminal tail region (BN, hereafter) as fusion protein while the C-terminal was attached with an 8 × His tag to facilitate the protein expression and purification (*72*). The whole tag-attached D1R was then cloned into the standard pFastBac (Thermo Fisher Scientific) vector. The prolactin precursor sequence was inserted into the N terminus before the FLAG tag as signaling peptide to assist cell membrane localization and increase expression of D1R. The NanoBiT tethering method was used to facilitate assembly of the D1R complexes, in which the C terminus of D1R was fused to the large part of NanoBiT (LgBiT), and the C terminus of G_β_ was fused to the small part of NanoBiT (SmBiT) (*73*). A miniGαs format of G_αs_ with dominant negative mutations, G226A and A366S, was engineered (miniG_αs__DN) according to the previous study (*36*). All the three G_s_ protein complex components—miniG_αs__DN, rat G_β1_, and bovine G_γ2_—were constructed into pFastbac vector separately.

### Expression, assembly, and purification of D1R-G_s_ complexes

Before expression, the recombinant baculoviruses containing D1R, miniG_αs__DN, G_β1_, and G_γ2_, respectively, were prepared using the Bac-to-Bac baculovirus expression system (Thermo Fisher Scientific). Cell cultures were grown to a density of 4 × 10^6^ cells/ml in ESF 921 serum-free medium (Expression Systems). For the expression of the D1R-G_s_ complexes, Sf9 cells (*Spodoptera frugiperda*; Expression Systems, catalog no. 94-001F; RRID:CVCL_0549) were coinfected with the four types of baculoviruses prepared at 1:1:1:1 ratio. Forty-eight hours after infection, the cultures were harvested by centrifugation at 1300*g* (Thermo Fisher Scientific, H12000) for 20 min and frozen at −80°C for further usage.

For the purification of D1R-G_s_ in complex with SKF81297/LY3154207/BMS-A1, cell pellets from 1-liter culture were thawed at room temperature and resuspended in buffer containing 20 mM Hepes (pH 7.2), 75 mM NaCl, 5 mM CaCl_2_, 5 mM MgCl_2_, 10% Glycerol, 0.3 mM TCEP, protease inhibitor cocktail (Bimake, 1-/100-ml suspension). The cell pellets were dounced to homogeneity and subsequently added with 10 μM SKF81297 (Tocris) to induce complexes formation on cell membrane. Half an hour later, the cell suspension was further supplied with 20 μM LY3154207, treated with apyrase (25 mU/ml; NEB) and incubated at room temperature for another 1 hour. Last, the cell membrane in suspension was solubilized directly by 0.5% (w/v) lauryl maltose neopentyl glycol (LMNG; Anatrace) and 0.1% (w/v) cholesteryl hemisuccinate tris salt (CHS; Anatrace) and supplemented with 50 μM BMS-A1 and Nb35 [10 μg/ml; expressed and purified in-house in *Escherichia coli* BL21(DE3) as previously described (*36*)]. The membrane was solubilized for 3 hours at 4°C before separation by ultracentrifugation. The sample was centrifuged at 100,000*g* (Ti45, Beckman) for 45 min. The isolated supernatant was then incubated for 2 hours at 4°C directly with FLAG resin (Smart-Lifesciences) pre-equilibrated with buffer containing 20 mM Hepes (pH 7.2) and 100 mM NaCl. After binding, FLAG resin was washed with 20 column volumes of 20 mM Hepes (pH 7.2), 100 mM NaCl, 0.3 mM TCEP, 10 μM SKF81297, 10 μM LY3154207, 25 μM BMS-A1, 0.01% (w/v) LMNG, and 0.002% (w/v) CHS. The complex was eluted with 10 column volumes of 20 mM Hepes (pH 7.2), 100 mM NaCl, 0.3 mM TCEP, 10 μM SKF81297, 10 μM LY3154207, 25 μM BMS-A1, 0.01% (w/v) LMNG, 0.002% (w/v) CHS, and FLAG peptide (200 μg/ml). The protein was then concentrated and loaded onto a Superose 6 Increase 10/300 column (GE Healthcare) pre-equilibrated with buffer containing 20 mM Hepes (pH 7.2), 100 mM NaCl, 0.3 mM TCEP, 0.00075% (w/v) LMNG, 0.00025% (w/v) glyco-diosgenin (GDN, Anatrace), 0.0002% (w/v) CHS, 10 μM SKF81297, 10 μM LY3154207, and 25 μM BMS-A1. The fractions for the monomeric complex were collected and concentrated for electron microscopy experiments.

For the purification of D1R-G_s_ in complex with SKF81297/LY3154207/BMS-A1/UNC10062, all the purification processes are the same as the above with the following exceptions. The UNC10062 compound was added at the concentration of 50 μM with 10 μM SKF81297 (Tocris). In the later purification procedures, the buffer for washing and elution were all supplemented with 10 μM SKF81297, 10 μM LY3154207, 25 μM BMS-A1, and 25 μM UNC10062. The protein was loaded onto a Superdex 200 Increase 10/300 column (GE Healthcare) after concentration.

For the purification of D1R-G_s_ in complex with DA/LY3154207/BMS-A1/UNC9815, all the purification processes are the same as the above with the following exceptions. The UNC9815 compound was added at the concentration of 50 μM with 100 μM DA (MedChemExpress). In the later purification procedures, the buffer for washing and elution were all supplemented with 50 μM DA, 10 μM LY3154207, 25 μM BMS-A1, and 25 μM UNC9815. The protein was loaded onto a Superdex 200 Increase 10/300 column (GE Healthcare) after concentration.

### Cryo-EM grid preparation and data collection

For the preparation of cryo-EM grids, 3 μl of the purified complexes at 10.9 mg/ml for the D1R-G_s_ complex with SKF81297/LY3154207/BMS-A1, 16.0 mg/ml for the D1R-G_s_ complex with SKF81297/LY3154207/BMS-A1/UNC10062, and 11.0 mg/ml for the D1R-G_s_ complex with DA/LY3154207/BMS-A1/UNC9815 were applied onto a glow-discharged holey carbon grid (Quantifoil R1.2/1.3). Grids were plunge-frozen in liquid ethane using Vitrobot Mark IV (Thermo Fisher Scientific). Frozen grids were transferred to liquid nitrogen and stored for data acquisition. Cryo-EM imaging was collected on a Titan Krios at 300 kV using Gatan K3 Summit detector and Falcon 4 direct electron detection device in the Cryo-Electron Microscopy Research Center, Shanghai Institute of Materia Medica, Chinese Academy of Sciences (Shanghai, China). A total of 6244 movies for the D1R-G_s_ complex with SKF81297/LY3154207/BMS-A1 were collected on a Titan Krios equipped with a Gatan K3 direct electron detector with a pixel size of 0.824 Å. Images were taken at a dose rate of about 15 e^−^/pixel/s with a defocus ranging from −1.0 to −3.0 μm using the EPU 3.8.1 software (FEI Eindhoven, Netherlands). The total exposure time was 2.35 s, and 36 frames were recorded per micrograph. A total of 7198 movies for the D1R-G_s_ complex with SKF81297/LY3154207/BMS-A1/UNC10062 and 8517 movies for the D1R-G_s_ complex with DA/LY3154207/BMS-A1/UNC9815 were collected on a Titan Krios equipped with a Falcon 4 direct electron detection device at 300 kV. Images were taken with a pixel size of 0.73 Å, a defocus ranging from −0.8 to −1.8 μm using the EPU software (FEI Eindhoven, Netherlands) with total dose of 50 e^−^/Å^2^ over 2.5 s exposure on each EER format movie. Each movie was divided into 936 frames during motion correction.

### Image processing and map construction

For the D1R-G_s_-DA-LY3154207-BMS-A1-UNC9815, all collected movie stacks were motion-corrected and dose-weighted in RELION 4.0 (*74*). The contrast transfer function (CTF) parameters were estimated using the CTFFIND4 program within cryoSPARC v4 (*75*). Initial particle picking was performed with the Blob Picker on micrographs that had CTF parameters determined. The extracted particles underwent multiple rounds of 2D classification, yielding a subset of 390,276 particles. These were further refined through several iterations of ab initio reconstruction and heterogeneous refinement, ultimately converging on a well-defined particle subset. Subsequent heterogeneous refinement, nonuniform refinement, and local refinement were conducted in cryoSPARC v4 (*76*), resulting in a final reconstruction at a global resolution of 2.44 Å, derived from 209,112 particles. The other maps were obtained following procedures similar to those described above.

### Model building and refinement

The structure of D1R-G_s_-SKF81297-LY3154207 [Protein Data Bank (PDB): 7LJC] was used as initial model for model rebuilding and refinement against the cryo-EM density maps of D1R-G_s_ complexes. The initial models were docked into the cryo-EM density maps using ChimeraX followed by iterative manual adjustment and rebuilding in COOT 0.9.8.95 (*77*). Real-space refinement and reciprocal space refinement were performed using Phenix 1.19.2_4158 programs (*78*). Structure figures were prepared in ChimeraX 1.8.

### β-Arrestin recruitment DiscoverX PathHunter assay

Agonist-mediated D1R β-arrestin recruitment was determined using the DiscoveRx PathHunter complementation assay, as previously described by our laboratory (*24*, *79*). Briefly, CHO-K1 cells (*Cricetulus griseus*; Eurofins DiscoverX; parental cell-line RRID:CVCL_0214) stably expressing the D1R were maintained in Ham’s F12 supplemented with 10% fetal bovine serum, penicillin (100 U/ml), streptomycin (100 μg/ml), G418 (800 μg/ml), and hygromycin (300 μg/ml), at 37°C, 5% CO_2_, and 90% humidity. Cells were seeded in Cell Plating Media 2 (Eurofins DiscoverX, Fremont, CA) at a density of 2625 cells per well in 384-well black, clear-bottom plates. Following an 18- to 24-hour incubation, cells were treated with the indicated concentrations of compounds in phosphate-buffered saline (PBS) containing 0.2 mM sodium metabisulfite and then incubated at 37°C for 90 min. The Tropix Gal-Screen buffer and substrate (Applied Biosystems, Bedford, MA) were added to cells according to the manufacturer’s recommendations and incubated for 30 min at room temperature. Luminescence signal was measured using a PHERAstar plate reader (BMG LABTECH, Cary, NC). Data were collected as RLUs and subsequently normalized to a percentage of the control luminescence seen with a maximum concentration of DA.

### Transient transfections

Human embryonic kidney (HEK) 293 cells [*Homo sapiens*; American Type Culture Collection (ATCC), catalog no. CRL-1573; RRID:CVCL_0045] were grown in Dulbecco’s modified Eagle’s medium (DMEM) supplemented with 10% fetal bovine serum and maintained at 37°C in a humidified incubator containing 5% CO_2_. The day before transfection, cells were plated in either 100-mm tissue culture dishes at 4 × 10^6^ cells per dish or six-well plates at 1 × 10^6^ cells per well in serum-free DMEM. The cells were transfected with the indicated DNA constructs using polyethylenimine (PEI) as the transfection reagent at a ratio of 3:1 (μL PEI:μg DNA). Twenty-four hours after transfection, the medium was changed to DMEM supplemented with 10% fetal bovine serum, and cells were used for experiments the next day.

### CAAX β-arrestin recruitment BRET assay

HEK293 cells were transiently transfected with the indicated D1R constructs, green fluorescent protein (GFP)–CAAX [which is anchored in the plasma membrane (*80*)] and β-arrestin2–Rluc2. Cells were harvested with calcium-free Earle’s balanced salt solution (EBSS−), plated in 96-well white plates at 20,000 cells per well in Dulbecco’s PBS (DPBS) and incubated at room temperature for 45 min. Cells were then incubated with 2 μM Prolume Purple (Nanolight Technology, Pinetop, AZ, USA) for 5 min and then stimulated with appropriate concentrations of test compounds for 5 min in <3% dimethyl sulfoxide (DMSO). The BRET signal was determined by quantifying and calculating the ratio of the light emitted by GFP (515 nm) over Rluc2 (410 nm) using a PHERAstar FSX Microplate Reader (BMG Labtech, Cary, NC, USA).

### Direct β-arrestin recruitment BRET assay

HEK293 cells were transiently transfected with the indicated D1R-Rluc8 constructs and β-arrestin2–mVenus as previously described (*81*). Cells were harvested with EBSS−, plated in 96-well white plates at 20,000 cells per well in DPBS, and incubated at room temperature for 45 min. Cells were incubated with 5 μM coelenterazine h (Nanolight Technology, Pinetop, AZ, USA) for 5 min and then stimulated with appropriate concentrations of test compounds for 5 min in <3% DMSO. The BRET signal was determined by quantifying and calculating the ratio of the light emitted by mVenus (525 nm) over that emitted by Rluc8 (485 nm) using a PHERAstar FSX Microplate Reader (BMG Labtech, Cary, NC, USA).

### cAMP CAMYEL BRET assay

HEK293 cells transiently expressing the indicated D1R constructs and the CAMYEL cAMP biosensor (yellow fluorescence protein-Epac-Rluc) (*82*) were harvested with EBSS−, plated in 96-well white plates at 20,000 cells per well in DPBS, and incubated at room temperature for 45 min. Cells were incubated with 5 μM coelenterazine h (Nanolight Technology, Pinetop, AZ, USA) for 5 min and then stimulated with appropriate concentrations of test compounds for 5 min in <3% DMSO. The BRET signal was determined by quantifying and calculating the ratio of the light emitted by mVenus (525 nm) over that emitted by Rluc8 (485 nm) using a PHERAstar FSX Microplate Reader (BMG Labtech, Cary, NC, USA).

### TRUPATH G_s_ protein dissociation BRET assay

The TRUPATH G_s_ protein dissociation BRET assay (*83*) was performed as follows. HEK293T cells (*H. sapiens*; ATCC, catalog no. CRL-3216; RRID:CVCL_0063) were seeded in 6-cm dishes at 1.3 × 10^6^ to 1.5 × 10^6^ cells per dish and transfected 16 to 20 hours later. For agonist-induced D1R Gs protein dissociation, receptor, G_sL_-RLuc8, G_β1_, and G_γ1_-GFP2 were transfected at a 1:1:1:1 ratio, with 250 ng of each plasmid per 6-cm dish. DNA complexes were formed with PEI at 2 μl per μg DNA, and DNA was diluted in Opti-MEM to a final concentration of 10 ng/μl.

One day after transfection, cells were harvested with PBS, resuspended, and seeded into poly-d-lysine–coated white 96-well plates with clear bottoms at a density of 3.0 × 10^4^ to 5.0 × 10^4^ cells per well. After overnight incubation, the growth medium was replaced with 80 μl of assay buffer (1× Hank’s balanced salt solution, pH 7.4). Cells were stimulated with 20 μl of ligands for 5 min, followed by addition of 20 μL of freshly prepared 50 μM coelenterazine 400a. After 5-min equilibration at room temperature, luminescence was measured on an EnVision plate reader using 410-nm and 515-nm emission filters. Three sequential readings were acquired from each well, and the most stable value was used for analysis. The BRET ratio was calculated as the emission intensity at 515 nm divided by that at 410 nm.

### Molecular modeling

Based on the cryo-EM structures reported in this study, the D1R-G_s_ complex models for the MD simulations were constructed using MODELLER (version 10.6) (*84*) to add key residues missing in the cryo-EM structures. Specifically, we added residue 20 of the N terminus, residues 169 to 184 of ECL2, residues 299 to 306 of ECL3, residues 241 to 263 of ICL3, and residues 346 to 348 of the Helix 8 (H8) using corresponding coordinates from a well equilibrated D1R-G_s_ model derived from our previous MD simulation study (*39*). In addition, we ensured the formation of two disulfide bonds: C96^3.25^-C186^ECL2.50^ and C298^6.61^-C307^ECL3^ with constraints.

A total of 50 initial models were generated and assessed using the discrete optimized protein energy (DOPE) score (*85*). The model with the lowest DOPE score, which also displayed loop orientations compatible with the membrane environment (i.e., not clashing with lipid molecules), was selected for the subsequent step.

### MD simulations

To build the MD simulation systems, the resulting D1R models generated with Modeller were processed using the Protein Preparation Wizard in Maestro (Schrödinger, version 2024.2). During this process, the hydrogen atoms were added, the protonation states of all titratable residues were assigned on the basis of on the p*K*_a_ values predicted by PROPKA (*86*), and Epik (*87*) was used to determine the protonation and tautomeric states of the bound ligands in their bound conformations. We modeled residues D70^2.50^ and D120^3.49^ in their protonated (neutral) states, consistent with the current understanding of aminergic GPCRs in the active conformation. The nitrogen atoms of the orthosteric ligands (SKF and DA) were predicted to be protonated, carrying a positive charge. All histidine residues were kept neutral and C347 in H8 was modeled as palmitoylated.

The simulation systems were then constructed using the Desmond System Builder (Schrödinger, version 2024.2). Each D1R complex was embedded in a POPC lipid bilayer and solvated with simple point charge water model. The overall charge of each system was neutralized by adding appropriate counter ions, and 0.15 M NaCl was included to reproduce physiological ionic strength during MD simulations. The resulting system consists of ∼165,000 atoms. The equilibrated system has approximate dimensions of ∼110 × 105 × 140 Å^3^.

MD simulations were performed with the Desmond MD engine (D. E. Shaw Research, New York, NY, USA) using OPLS4 force field. Ligand force field parameters for LY3154207 and UNC9815 were derived by quantum-mechanical optimization using the Schrödinger Force Field Builder (version 2024.2). Following our previously established GPCR simulation protocols (*39*), each system was first minimized and equilibrated while applying harmonic positional restraints to ligand heavy atoms and protein backbone atoms (*k* = 1.0 kcal/mol/Å^2^). Simulations were carried out in the NPγT ensemble, with constant temperature (310 K) maintained using Langevin dynamics. Pressure was maintained at 1 atm using the hybrid Nose-Hoover Langevin piston method applied to an anisotropic flexible periodic cell.

During equilibration, a 1-fs integration time step was used and later increased to 2 fs for the production phase. Nonbonded interactions were calculated with a 12-Å cutoff. Harmonic restraints were gradually released during equilibration: First from the D1R loops, then from the transmembrane helices and bound ligands. Before production runs at 310 K, all restraints on the D1R were removed, while most α-carbon restraints on the G protein were retained, except for Gα residues 303 to 351 (α4-αG loop and α4 helix) and 383 to 394 (α5 helix). Long timescale MD simulations were performed on V100x and A100 GPUs, with system coordinates saved every 600 ps. For each condition, two to six independent trajectories were generated with different random seeds. In total, 41 trajectories were obtained across all D1R systems (with and without PAMs), corresponding to an aggregated simulation time of ∼40 μs (table S10).

For the G_s_ protein-free D1R systems, we used the corresponding D1R-G_s_ complex models generated using Modeller, with the G_s_ protein removed, thereby ensuring the starting D1R conformation in the G protein–free system matched that in the G_s_ protein–bound system. This match allowed direct assessment of how the G_s_ protein binding affects receptor dynamics. The resulting G protein–free systems were then processed, minimized, equilibrated, and subjected to the production runs using the protocols described above for the D1R-G_s_ complexes.

### Protein interaction analyzer

PIA calculates pairwise distances between residues using either backbone Cα atoms or the centers of mass of side-chain heavy atoms and then determines distance differences between two conditions to assess conformational changes. The results are visualized as a heatmap, with the top-right and bottom-left halves representing Cα and side chain–based distance differences, respectively. A key advantage of this method is that it does not rely on structural superposition, enabling robust analysis of rearrangement trends between two conditions (*48*). In this study, pairwise distance differences of D1R binding-site residues were computed between inactive (*46*) and active cryo-EM structures, as well as between PAM-bound and unbound cryo-EM structures in the presence of orthosteric ligands (DA or SKF81297).

The binding-site analysis included following residues: 2.61, 2.64, 2.65, EL1.50, 3.28, 3.29, 3.32, 3.33, 3.36, 3.37, 3.40, 4.57, EL2.52, EL2.54, 5.38, 5.39, 5.42, 5.43, 5.46, 5.47, 5.50, 6.44, 6.48, 6.51, 6.52, 6.55, 6.56, 6.58, 6.59, 7.32, 7.35, 7.36, 7.39, 7.40, 7.42, and 7.43. Distance differences were plotted using a 1.0-Å cutoff. In the subsegment analysis, we defined the following subsegments in the transmembrane domain: TM1e (the extracellular section (e) of TM1, 1.30 to 1.36), TM1m (the middle section (m) of TM1, residues 1.37 to 1.45), TM1i (the intracellular section (i) of TM1, residues 1.46 to 1.59), TM2i (residues 2.38 to 2.51), TM2m (residues 2.52 to 2.60), TM2e (residues 2.61 to 2.66), TM3e (residues 3.22 to 3.35), TM3m (residues 3.36 to 3.40), TM3i (residues 3.41 to 3.55), TM4i (residues 4.39 to 4.49), TM4m (residues 4.50 to 4.55), TM4e (residues 4.56 to 4.62), TM5e (residues 5.36 to 5.45), TM5m (residues 5.46 to 5.50), TM5i (residues 5.51 to 5.63), TM6i (residues 6.30 to 6.43), TM6m (residues 6.44 to 6.48), TM6e (residues 6.49 to 6.60), TM7e (residues 7.32 to 7.43), and TM7i (residues 7.44 to 7.54).

### Pairwise RMSD calculations

Pairwise RMSD distributions were calculated for the PAMs (BMS-A1, UNC10062, and UNC9815) using VMD (*88*). For each trajectory, the first 100 frames were discarded. For each pair of frames, the frames were superimposed on the Cα atoms of the PAM binding-site residues (1.38, 1.39, 1.41, 1.42, 1.45, 1.48, 1.49, 7.36, 7.37, 7.40, 7.41, 7.44, 7.48, 7.51, 7.55, and 8.54), after which the RMSD was calculated using ligand heavy atoms. RMSD values computed between all frame pairs yielded pairwise RMSD distributions that quantify the stability and conformational dynamics of PAMs within their binding sites.

### Synthesis of UNC9815 and UNC10062

#### General information

All reagents were used as received from commercial sources (Sigma-Aldrich, TCI America, and Thermo Fisher Scientific). 4-(*tert*-Butyl) cyclohexanone **S1** and methyl 2-(4-hydroxyphenyl) acetate **S6** were purchased from Ambeed Scientific. The ^1^H and ^13^C nuclear magnetic resonance (NMR) spectra were recorded on a 400-MHz Bruker Avance spectrometer equipped with a broadband observe probe and a 500-MHz Bruker AVIII spectrometer equipped with a dual cryoprobe, respectively. Chemical shifts are reported in parts per million and were referenced to residual proton solvent signals. Microwave syntheses were conducted in a Biotage Initiator constant temperature microwave synthesizer. Flash column chromatography separations were performed by the use of the Teledyne Isco CombiFlash Rf using RediSep Rf silica gel columns eluting with hexanes/ethyl acetate (0 to 100%). Thin-layer chromatography (TLC) was performed on Analtech UNIPLATE silica gel GHLF plates (gypsum inorganic hard layer with fluorescence). TLC plates were developed using iodine vapor, potassium permanganate stain or ceric ammonium molybdate stain, as required. Compound purity was measured on the basis of peak integration (area under the curve) from ultraviolet/visible absorbance (at 214 nm) on a Waters Acquity ultra performance LC system with a photodiode array detector coupled to a Waters Acquity single quadrupole detector mass spectrometer with an ESI source. Compound identity was determined on the basis of HRMS analysis. HRMS samples were analyzed at the UNC Department of Chemistry Mass Spectrometry Core Laboratory using a Q Exactive HF-X mass spectrometer.

#### Synthetic procedures and characterization of UNC9815

2-(4-(*tert*-Butyl) cyclohexylidene)-2-cyanoacetamide (**S3**). To a solution of 4-(*tert*-butyl) cyclohexanone **S1** (500 mg, 3.24 mmol) and 2-cyanoacetamide **S2** (273 mg, 3.24 mmol, 1.0 equiv) in toluene (50 ml) was added ammonium acetate (250 mg, 3.24 mmol, 1.0 equiv) and acetic acid (185 μl, 3.24 mmol, 1.0 equiv) at room temperature. The reaction was heated to reflux and water removed azeotropically using a Dean-Stark trap. After complete evolution of water, the reaction was cooled to room temperature. Water (50 ml) and ethyl acetate (25 ml) were added and the layers separated. The organic layer was dried over sodium sulfate, and the solvent evaporated under vacuum. The residue was purified by flash chromatography (40% ethyl acetate in hexanes) to give **S3** (475 mg, 2.16 mmol, 67% yield) as a white solid. ^1^H-NMR (500 MHz, CDCl_3_): δ 0.87 (d, *J* = 1.3 Hz, 9H), 1.17 to 1.38 (m, 3H), 1.97 to 2.04 (m, 2H), 2.04 to 2.11 (m, 1H), 2.23 to 2.34 (m, 1H), 3.02 (ddd, *J* = 13.9, 3.7, 2.3 Hz, 1H), 3.94 to 4.03 (m, 1H), and 6.00 (d, *J* = 128.1 Hz, 2H).

2-Amino-6-(*tert*-butyl)-4,5,6,7-tetrahydrobenzo[b]thiophene-3-carboxamide (**S4**). To a solution of **S3** (400 mg, 2.16 mmol) in ethanol (10 ml) was added sulfur (76 mg, 2.37 mmol, 1.1 equiv) and morpholine (186 μl, 2.16 mmol, 1.0 equiv) at room temperature. The reaction was heated to 50°C and stirred for 5 hours. After completion, the solvent was evaporated under vacuum. The residue was purified by flash chromatography (40% ethyl acetate in hexanes) to give **S4** (489 mg, 1.94 mmol, 90% yield) as a white solid. ^1^H-NMR (500 MHz, DMSO-*d*_6_): δ 0.91 (s, 9H), 1.13 to 1.27 (m, 1H), 1.36 to 1.45 (m, 1H), 1.93 (d, *J* = 9.4 Hz, 1H), 2.21 to 2.30 (m, 1H), 2.47 (dd, *J* =15.7, 4.9 Hz, 1H), 2.60 (d, *J* = 13.2 Hz, 1H), 2.69 (dd, *J* = 15.7, 4.9 Hz, 1H), 6.49 (s, 2H), and 6.92 (s, 2H). These data match those previously reported (*24*).

2-Amino-6-(*tert*-butyl) benzo[b]thiophene-3-carboxamide (**S5**). To a solution of **S4** (489 mg, 1.94 mmol) in 1,4-dioxane (10 ml) was added chloranil (953 mg, 3.88 mmol, 2.0 equiv), and the reaction was heated to reflux for 17 hours while monitoring the progress of the reaction using ultra-performance liquid chromatography–mass spectrometry. The solvent was evaporated under vacuum and the residue partitioned between dichloromethane and water. The biphasic mixture was filtered through celite, and the layers were separated. The organic layer was dried over sodium sulfate and evaporated under vacuum. The residue was purified by flash chromatography (15% ethyl acetate in hexanes) to give **S5** (341 mg, 1.37 mmol, 71% yield) as a pale pink solid. ^1^H-NMR (400 MHz, CDCl_3_): δ 1.35 (s, 9H), 5.65 (s, 2H), 6.55 (s, 2H), 7.37 (dd, *J* = 8.5, 1.9 Hz, 1H), 7.51 (dd, *J* = 8.5, 0.6 Hz, 1H), 7.57 (dd, *J* = 1.9, 0.6 Hz, 1H); ^13^C-NMR (100 MHz, CDCl_3_): δ 31.6, 34.8, 101.5, 118.98, 119.02, 123.5, 129.9, 134.4, 145.6, 162.6, and 168.6.

*N*-(6-(*tert*-Butyl)-3-carbamoylbenzo[b]thiophen-2-yl) isonicotinamide (**UNC9815**). To a solution of isonicotinic acid (12.0 mg, 0.10 mmol) and catalytic amount (10 μl) of *N*,*N*′-dimethylformamide (DMF) in anhydrous dichloromethane (2 ml) was added oxalyl chloride (13 μl, 0.15 mmol, 1.5 equiv), and the reaction was stirred for 2 hours at room temperature. The solvent was evaporated under vacuum, the residue was dissolved in anhydrous dichloromethane (2 ml), **S5** (25.0 mg, 0.10 mmol, 1.0 equiv) and pyridine (16 μl, 0.20 mmol, 2.0 equiv) were added, and the reaction was stirred for 2 hours at room temperature. The progress of the reaction was monitored by TLC, and, upon completion, the solvent was removed under vacuum. The residue was extracted with ethyl acetate (25 ml) and filtered, and again the solvent was removed under vacuum. Last, the residue was purified by flash chromatography (5% methanol in dichloromethane) to afford *N*-(6-(*tert*-butyl)-3-carbamoylbenzo[b]thiophen-2-yl) isonicotinamide **UNC9815** (25.0 mg, 0.07 mmol, 69% yield) as an off-white solid. TLC (DCM:MeOH, 95:5, v/v): *Rf* = 0.5; ^1^H NMR (400 MHz, DMSO-*d*_6_) δ 1.37 (s, 9H), 7.55 (dd, *J* = 8.7, 1.9 Hz, 1H), 7.84 to 7.87 (m, 2H), 7.91 (s, 2H), 7.99 (d, *J* = 8.6 Hz, 1H), 8.01 (d, *J* = 1.8 Hz, 1H), 8.88 to 8.92 (m, 2H), 13.43 (s, 1H); ^13^C NMR (100 MHz, DMSO-*d*_6_) δ 31.3, 34.6, 111.2, 118.6, 120.9, 121.7, 123.4, 130.7, 133.9, 139.2, 146.1, 146.8, 151.0, 162.0, 167.6; UPLC purity = 99%; HRMS [mass/charge ratio (*m*/*z*)]: [M + H]^+^ calculated for C_19_H_20_N_3_O_2_S, 354.1271; found, 354.1268.

#### Synthetic procedures and characterization of UNC10062

Methyl 2-(4-(benzyloxy) phenyl) acetate (**S7**). According to the protocol of Qiang Liu and co-workers (*89*). A solution of methyl 2-(4-hydroxyphenyl) acetate **S6** (1.53 g, 9.19 mmol), benzyl bromide (1.57 g, 9.19 mmol, 1.0 equiv) and potassium carbonate (3.81 g, 27.58 mmol, 3.0 equiv) in DMF (20 ml) was stirred at room temperature for 15 hours. Water (50 ml) was added, and the reaction was extracted with EtOAc (3 × 50 ml). The combined organic layers were washed with brine, dried with Na_2_SO_4_, and concentrated under reduced pressure. The mixture was further purified by flash chromatography to give **S7** (2.01 g, 7.84 mmol, 85% yield). ^1^H-NMR (400 MHz, CDCl_3_): δ 3.57 (s, 2H), 3.69 (s, 3H), 5.05 (s, 2H), 6.91 to 6.96 (m, 2H), 7.18 to 7.23 (m, 2H), and 7.29 to 7.46 (m, 5H).

Methyl 2-(4-(benzyloxy) phenyl)-2-methylpropanoate (**S8**). According to the protocol of Qiang Liu and coworkers (*89*). A solution of **S7** (2.00 g, 7.80 mmol) in tetrahydrofuran (THF) (20 ml) was slowly added to a slurry of sodium hydride [935.7 mg, 60 weight % (wt %), 23.39 mmol, 3.0 equiv] in THF (20 ml) at 0°C and then stirred at room temperature for 1 hour. The reaction was cooled to 0°C, and iodomethane (3.32 g, 23.39 mmol, 3.0 equiv) was added. The mixture was stirred at room temperature for 15 hours. Water (50 ml) was added, and the reaction was extracted with EtOAc. The combined organic layers were dried with Na_2_SO_4_ and concentrated under reduced pressure. The mixture was further purified by flash chromatography to give **S8** (0.6489 g, 2.282 mmol, 29% yield). ^1^H-NMR (400 MHz, CDCl_3_): δ 1.55 (s, 6H), 3.63 (s, 3H), 5.03 (s, 2H), 6.87 to 6.96 (m, 2H), 7.20 to 7.27 (m, 2H), and 7.28 to 7.43 (m, 5H).

Methyl 2-methyl-2-(4-oxocyclohexyl) propanoate (**S9**). According to the protocol of Qiang Liu and co-workers (*89*). To a solution of methyl 2-(4-(benzyloxy) phenyl)-2-methylpropanoate **S8** (0.63 g, 2.22 mmol) in 1,2-dichloroethane (10 ml) in a Parr reactor 25-ml glass insert was added Pd/Al_2_O_3_ (0.14 g, 5 wt %). The 25-ml glass insert was placed in a 150-ml stainless steel Parr reactor under air. The Parr reactor was pressurized and depressurized with hydrogen gas three times before the indicated pressure (5 bar) was set. The reaction was stirred and heated at 60°C for 24 hours. The filtration of the catalyst through a pad of celite and concentration of the filtrate gave crude product which was further purified by flash chromatography to give **S9** (0.24 g, 1.19 mmol, 54% yield).^1^H-NMR (400 MHz, CDCl_3_) δ 1.17 (s, 6H), 1.49 (qd, *J* = 13.0, 5.1 Hz, 2H), 1.87 to 2.00 (m, 2H), 2.09 (ddd, *J* = 12.1, 9.2, 3.0 Hz, 1H), 2.29 to 2.47 (m, 4H), and 3.69 (s, 3H). These data are in agreement with those previously reported (*90*).

Methyl 2-(2-amino-3-carbamoyl-4,5,6,7-tetrahydrobenzo[*b*]thiophen-5-yl)-2-methylpropanoate (**S10**). A solution of **S9** (0.38 g, 1.90 mmol), 2-cyanoacetamide (239.3 mg, 2.85 mmol, 1.5 equiv), sulfur (60.82 mg, 1.90 mmol, 1.0 equiv), and morpholine (247.9 mg, 2.85 mmol, 1.5 equiv) in EtOH (3 ml) was heated under microwave irradiation at 120°C for 30 min. The reaction was concentrated under reduced pressure, and the residue was purified by flash chromatography to give **S10** (0.3394 g, 1.145 mmol, 60% yield). ^1^H-NMR (400 MHz, CD_3_CN): δ 1.14 (s, 3H), 1.18 (s, 3H), 1.34 (qd, *J* = 12.5, 5.2 Hz, 1H), 1.86 (ddd, *J* = 12.9, 5.4, 1.9 Hz, 1H), 1.96 to 2.05 (m, 1H), 2.31 to 2.48 (m, 2H), 2.51 to 2.62 (m, 1H), 2.76 (dd, *J* = 15.7, 5.3 Hz, 1H), 3.63 (s, 3H), 5.61 (s, 2H), 6.38 (s, 2H); ^13^C NMR (126 MHz, CD_3_CN): δ 22.3, 22.5, 25.6, 26.6, 28.2, 43.3, 45.9, 52.2, 108.1, 130.5, 161.6, 16.9, 178.5; UPLC purity = 99%; HRMS (*m/z*) [M + H]^+^: calculated for C_14_H_21_N_2_O_3_S, 297.1267; found, 297.1268.

Methyl 2-(3-carbamoyl-2-(isonicotinamido)-4,5,6,7-tetrahydrobenzo[*b*]thiophen-6-yl)-2-methylpropanoate (**UNC10062**). To a solution of **S10** (0.54 g, 1.81 mmol) and triethylamine (549.5 mg, 5.43 mmol, 3.0 equiv) in DCM (10 ml) at 0°C was slowly added isonicotinoyl chloride hydrochloride (526.8 mg, 2.72 mmol, 1.5 equiv). The reaction was stirred for 4 hours, quenched by the addition of saturated aqueous NaHCO_3_ (5 ml), and extracted with dichloromethane (3 × 20 ml). The combined layers were dried with Na_2_SO_4_ and purified by flash chromatography to give **UNC10062** (234.1 mg, 0.58 mmol, 32% yield). ^1^H-NMR (400 MHz, CDCl_3_): δ 1.23 (s, 3H), 1.24 (s, 3H), 1.49 (qd, *J* = 12.5, 5.2 Hz, 1H), 1.98 (dd, *J* = 12.9, 3.4 Hz, 1H), 2.07 to 2.17 (m, 1H), 2.50 to 2.63 (m, 1H), 2.69 (dd, *J* = 15.8, 4.8 Hz, 2H), 2.95 (dd, *J* = 14.6, 5.1 Hz, 1H), 3.71 (s, 3H), 5.94 (s, 2H), 7.82 to 7.88 (m, 2H), 8.83 (d, *J* = 5.8 Hz, 2H), 13.40 (s, 1H); ^13^C-NMR (101 MHz, CDCl_3_): δ 21.7, 22.5, 24.8, 25.8, 27.2, 41.9, 45.1, 51.9, 113.6, 121.1, 127.6, 128.5, 139.9, 146.7, 150.5, 161.4, 168.4, 177.7; UPLC purity = 97%; HRMS (*m/z*) [M + H]^+^: calculated for C_20_H_24_N_3_O_4_S, 402.1482; found, 402.1481.

## Supplementary Materials

### This PDF file includes:

Figs. S1 to S15 Tables S1 to S11 References
