# Supplementary material for: Multiple allosteric sites allow for synergistic enhancement of GPCR signaling
Source: Sci Adv. 2026 Oct 21;12(43):eaeh3401. doi: 10.1126/sciadv.aeh3401 (PMC13686013; doi:10.1126/sciadv.aeh3401)
Supplement: Supplementary file 1 [file sciadv.aeh3401_sm.pdf]

Science Advances
AAAS

Supplementary Materials for

Multiple allosteric sites allow for synergistic enhancement of GPCR signaling

Kai Wang *et al.*

Corresponding author: Kevin J. Frankowski, kevinf@email.unc.edu; Yue Wang, wangyue1@simm.ac.cn; Lei Shi, lei.shi2@nih.gov; David R. Sibley, sibleyd@ninds.nih.gov; Youwen Zhuang, youwen_zhuang@sjtu.edu.cn

Sci. Adv. **12**, eaeh3401 (2026)
DOI: 10.1126/sciadv.aeh3401

This PDF file includes:

Figs. S1 to S15
Tables S1 to S11
References

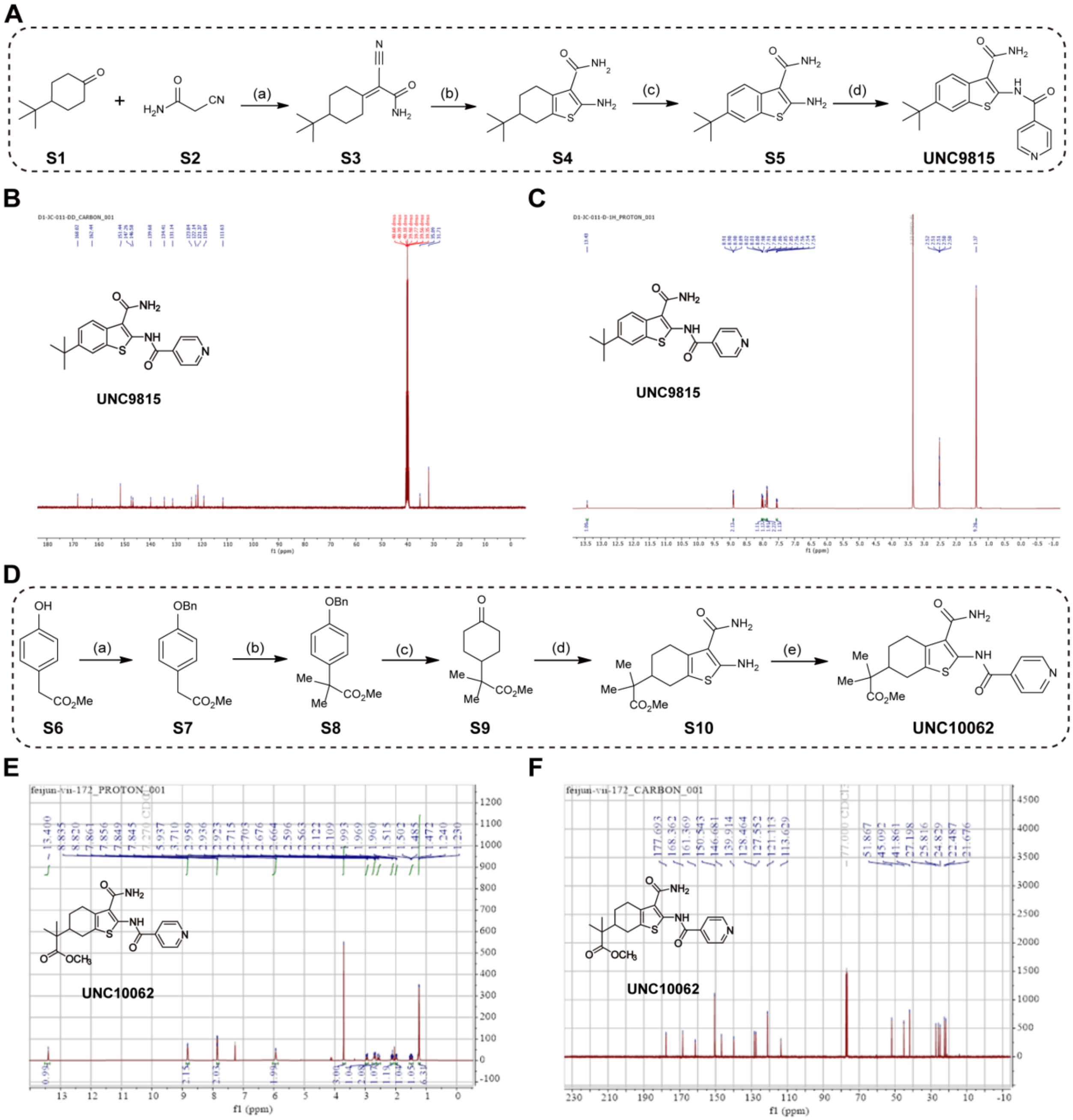

Fig. S1.

Synthesis and characterization of UNC compounds as novel D1R PAMs. (A, D) The procedures for synthesizing UNC compounds are described in **Materials and Methods. (A)** Synthetic route for UNC9815. Reagents and conditions: (a) Ammonium acetate (1.0 equiv), acetic acid (1.0 equiv), toluene, reflux, 67% yield; (b) sulfur (1.1 equiv), morpholine (1.0 equiv), ethanol, 50 °C, 5 h, 90% yield; (c) chloranil (2.0 equiv), 1,4-dioxane, reflux, 17 h, 71% yield; (d) isonicotinic acid (1.0 equiv), oxalyl chloride (1.5 equiv), dimethylformamide (catalytic), pyridine (2.0 equiv), dichloromethane, room temperature (RT), 2 h, 69% yield. **(B-C)** ^{1}H and ^{13}C NMR spectra of *N*-(6-(tert-butyl)-3-carbamoylbenzo[b]thiophen-2-yl) isonicotinamide (**UNC9815**). **(D)** Synthetic route for UNC10062. Reagents and conditions: (a) benzyl bromide (1.0 equiv), potassium carbonate (3.0 equiv), DMF, RT, 85% yield; (b) sodium hydride (3.0 equiv), methyl

iodide (3.0 equiv), THF, 0 °C to RT, 29% yield; (c) Pd/Al_2O_3 (5 wt%), hydrogen (5 bar), dichloroethane, 54% yield; (d) sulfur (1.0 equiv), 2-cyanoacetamide (1.5 equiv), morpholine (1.5 equiv), ethanol, 120 °C, microwave irradiation, 0.5 h, 60% yield; (e) isonicotinoyl chloride hydrochloride (1.5 equiv), triethylamine (3.0 equiv), dichloromethane, 0 °C, 4 h, 32% yield. **(E-F)** ^{1}H and ^{13}C NMR spectra of 2-(3-carbamoyl-2-(isonicotinamido)-4,5,6,7-tetrahydrobenzo[*b*]thiophen-6-yl)-2-methylpropanoate (**UNC10062**).

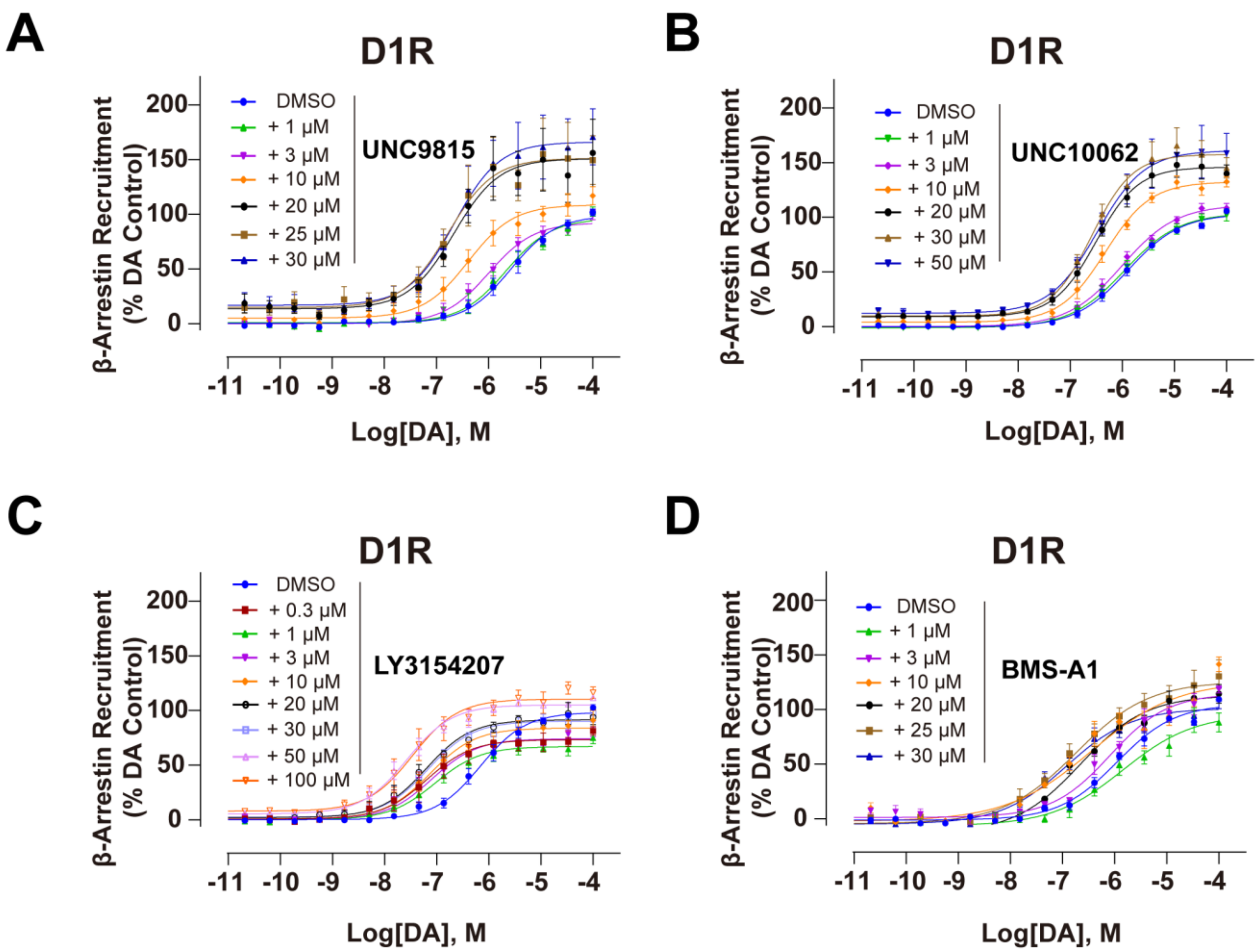

Fig. S2.

Dose-response curves for D1R PAM potentiation of DA-stimulated β-arrestin recruitment. **(A-D)** UNC9815, UNC10062, LY3154207, and BMS-A1 dose-dependently increase DA's potency in a saturable manner as measured in β-arrestin recruitment assays. Increasing concentrations of those PAMs were used to potentiate DA-stimulated β-arrestin recruitment using the DiscoverX assay as described in the Methods Section. Data are displayed as a percentage of the maximum DA control response in each experiment (mean ± SEM) of ≥ 3 experiments, each performed in triplicate. Curve parameters are shown in **Table S1.** **(A)**UNC9815 maximally increased DA potency at 30 µM, with an EC_{50} fold-shift of 17.1 ± 2.2**. **(B)**UNC10062 maximally increased DA potency at 50 µM, with an EC_{50} fold-shift of 3.9 ± 0.5**. **(C)**LY3154207 maximally increased DA potency at 50 µM, with an EC_{50} fold-shift of 27 ± 4.6* **(D)**BMS-A1 maximally increased DA potency at 20 µM, with an EC_{50} fold-shift of 6.3 ± 0.4**. The PAM EC_{50} fold-shift statistical comparisons were made to the control DA EC_{50} fold-shift values using a one-way ANOVA with Dunnett's post hoc comparison: *$p < 0.05$, **$p < 0.01$.

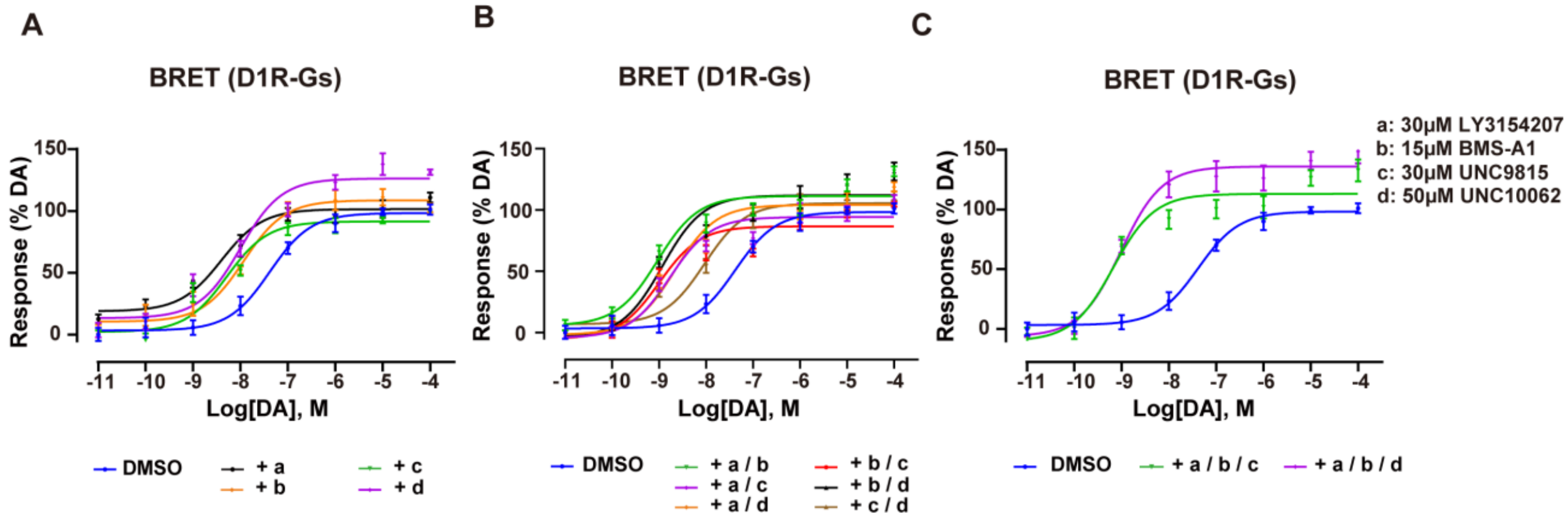

Figure S3.

Dose-response curves for D1R PAM potentiation of G protein dissociation stimulated by DA. Cooperative enhancement of D1R activation by LY3154207, BMS-A1, and UNC compounds (UNC9815 or UNC10062) in the presence of DA, measured by G protein dissociation using the BRET assay. **(A)** Concentration–response curves for individual PAMs (LY3154207, BMS-A1, UNC9815, and UNC10062) at fixed concentrations (30 µM, 15 µM, 30 µM, and 50 µM, respectively) in the presence of DA. **(B)** Effects of pairwise combinations of the four PAMs (at the same individual concentrations) on DA-induced dissociation. **(C)** Effects of triple combinations of the PAMs (using the same fixed concentrations). Responses are normalized to the maximal DA response in each experiment and shown as mean ± SEM from at least 3 independent experiments, each performed in triplicate. Curve-fitting parameters and statistical analyses are provided in **Table S3**.

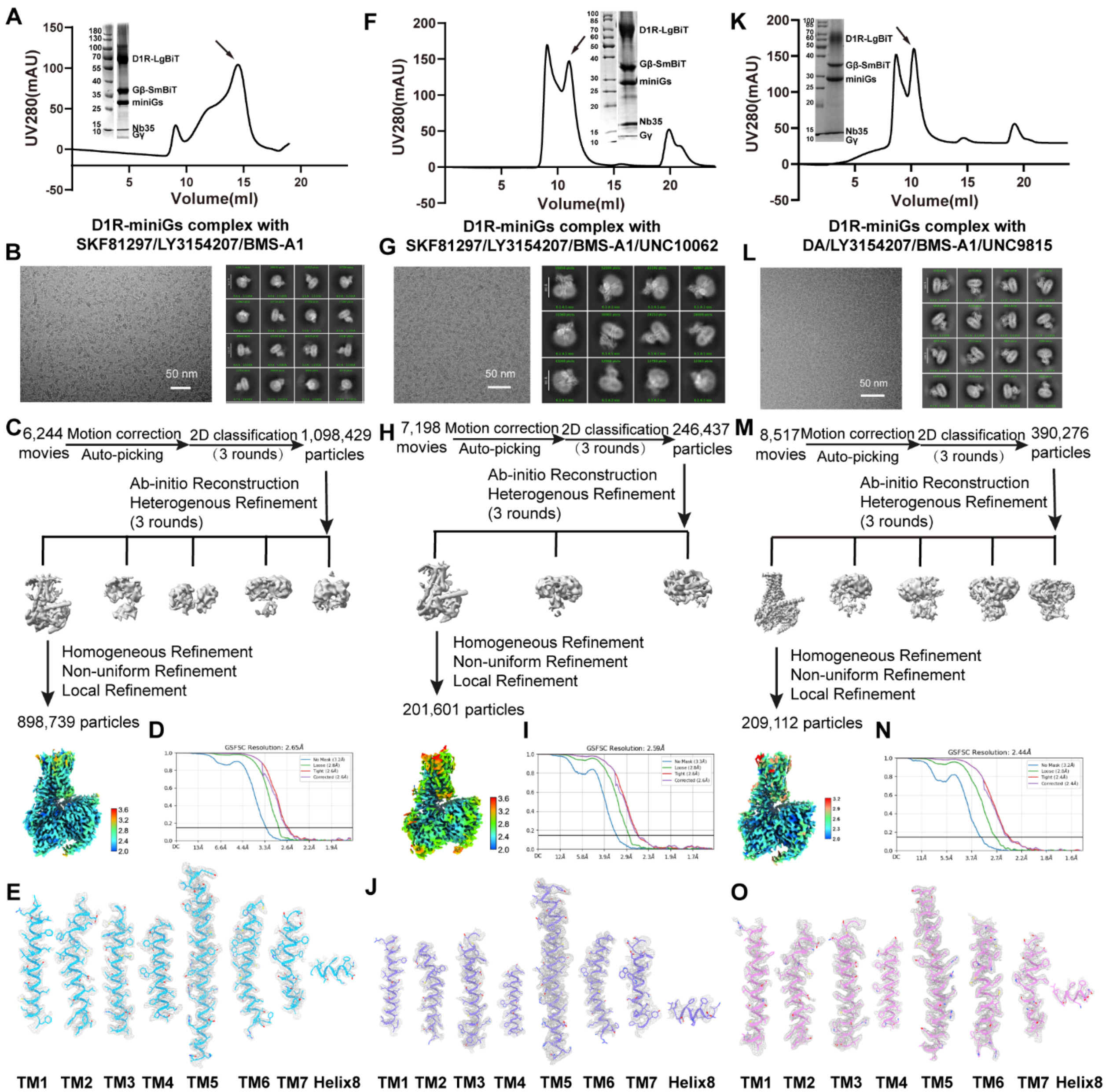

Fig. S4.

Cryo-EM data processing and structural determination of D1R-G_s complexes bound to multiple PAM combinations. (A, F, K) Size-exclusion chromatography profiles and SDS-PAGE analysis of purified D1R-G_s complex bound to SKF81297/LY3154207/BMS-A1, SKF81297/LY3154207/BMS-A1/UNC10062, and DA/LY3154207/BMS-A1/UNC9815, respectively. **(B, G, L)** Representative cryo-EM micrographs of the complexes and selected 2D class averages. **(C, H, M)** Cryo-EM data processing workflow. **(D, I, N)** Gold-standard Fourier shell correlation (FSC) curves with estimated global resolutions of 2.65 Å, 2.59 Å, and 2.44 Å at the FSC = 0.143 criterion. **(E, J, O)** High-resolution cryo-EM density maps of transmembrane helices (TM1-TM7) and Helix 8 from the three final reconstructions. The cryo-EM densities are shown at contour levels of 0.35 **(E)**, 0.32 **(J)**, and 0.35 **(O)**, respectively.

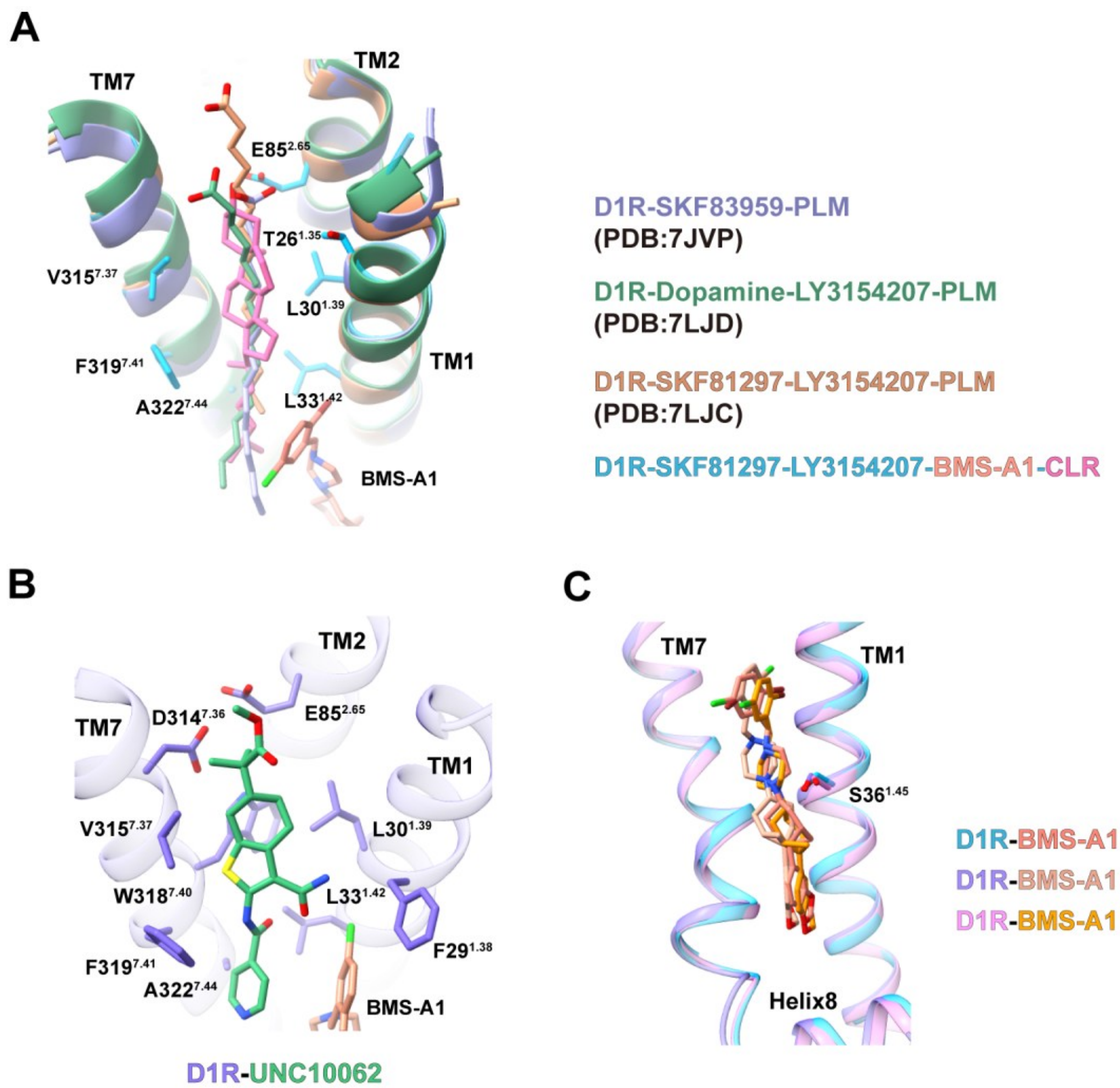

Fig. S5.

Additional ligands and pocket-lining residues in the D1R PAM pocket. (A) Structural comparison with available D1R cryo-EM structures shows that lipids are also present at a similar extracellular TM1–TM7 interface, including PLM modeled in D1R–SKF83959 (PDB: 7JVP), D1R–dopamine–LY3154207 (PDB: 7LJD), and D1R–SKF81297–LY3154207 (PDB: 7LJC), supporting that the upper TM1–TM7 pocket represents a pre-existing lipid-accessible cavity. Cholesterol molecule located above the BMS-A1 binding site in the D1R–G_s complex lacking UNC compounds. **(B)** Binding pose and key interactions of UNC10062 at PAM site 3 in the SKF81297-LY3154207-BMS-A1-UNC10062 bound D1R–G_s complex. **(C)** Structural comparison of BMS-A1 in the presence or absence of UNC compounds reveals conformational plasticity of the allosteric pocket.

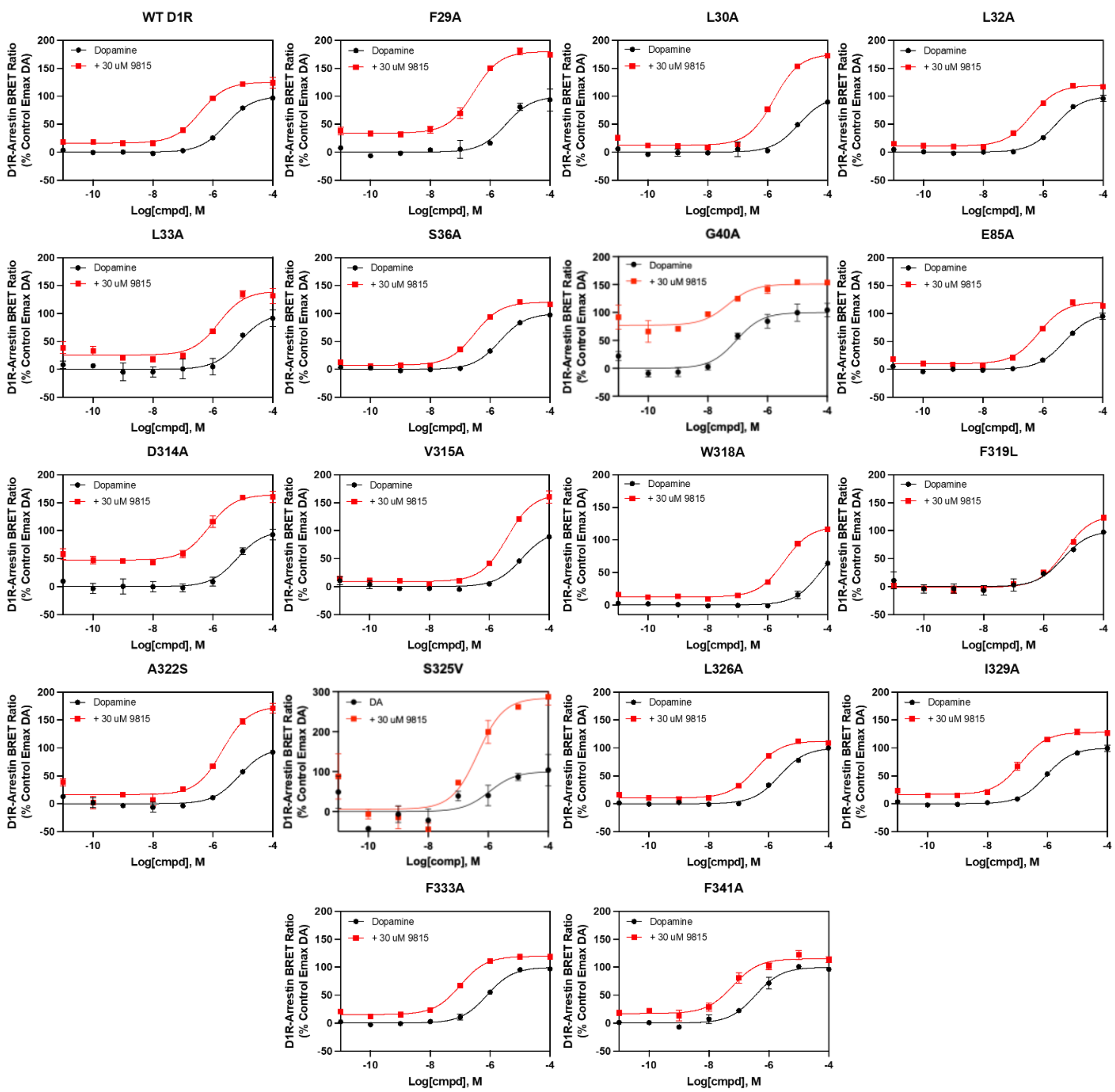

Fig. S6.
Effects of mutating D1R residues on UNC9815 PAM activity using β-arrestin recruitment assays. The BRET β-arrestin recruitment assay was conducted as described in the Methods Section. DA concentration-response curves in the presence or absence of 30 μM UNC9815 are shown for WT and each mutant D1R. The data are displayed as a percentage of the maximum DA control response in each experiment and are representative of ≥ 3 independent experiments each performed in duplicate. The EC_{50} fold-shift produced by UNC9815 is significantly reduced in mutations V315A, F319L and A322S. See **Table S5** for complete curve parameters and statistical analyses.

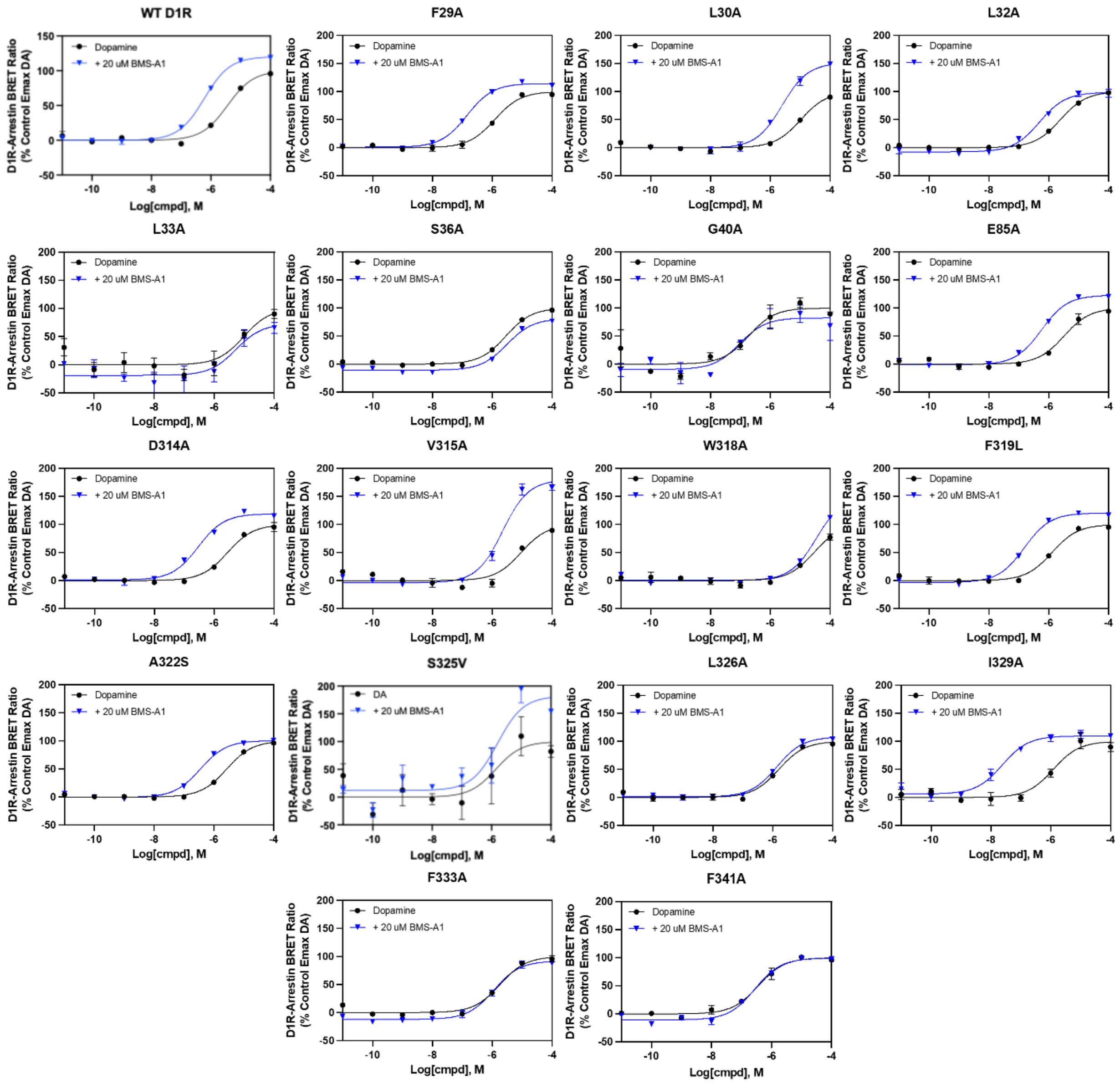

Fig. S7.

Effects of mutating D1R residues on BMS-A1 PAM activity using β-arrestin recruitment assays. The BRET β-arrestin recruitment assay was conducted as described in the Methods Section. DA concentration-response curves in the presence or absence of 20 μM BMS-A1 are shown for WT and each mutant D1R. The data are displayed as a percentage of the maximum DA control response in each experiment and are representative of ≥ 3 independent experiments each performed in duplicate. The EC_{50} fold-shift produced by BMS-A1 is significantly reduced in mutations L33A, S36A, G40A, L326A, F333A, and F341A. See **Table S6** for complete curve parameters and statistical analyses.

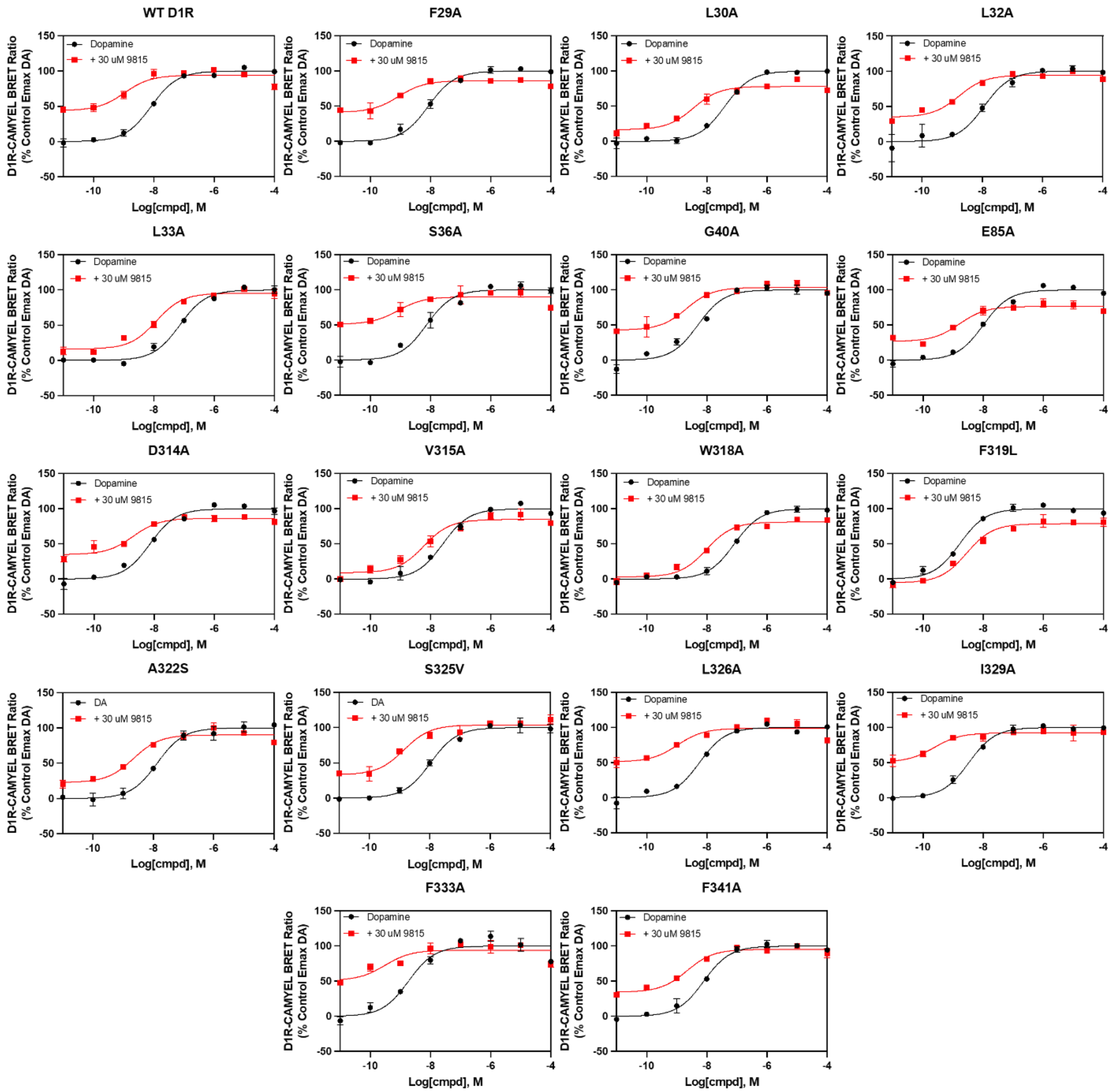

Fig. S8.

Effects of mutating D1R residues on UNC9815 PAM activity using CAMYEL BRET cAMP assays. The CAMYEL BRET cAMP assay was conducted as described in the Methods Section. DA concentration-response curves in the presence or absence of 30 µM UNC9815 are shown for WT and each mutant D1R. The data are displayed as a percentage of the maximum DA control response in each experiment and are representative of ≥ 3 independent experiments each performed in duplicate. The EC_{50} fold-shift produced by UNC9815 is significantly reduced in mutations V315A and F319L. See **Table S7** for complete curve parameters and statistical analyses.

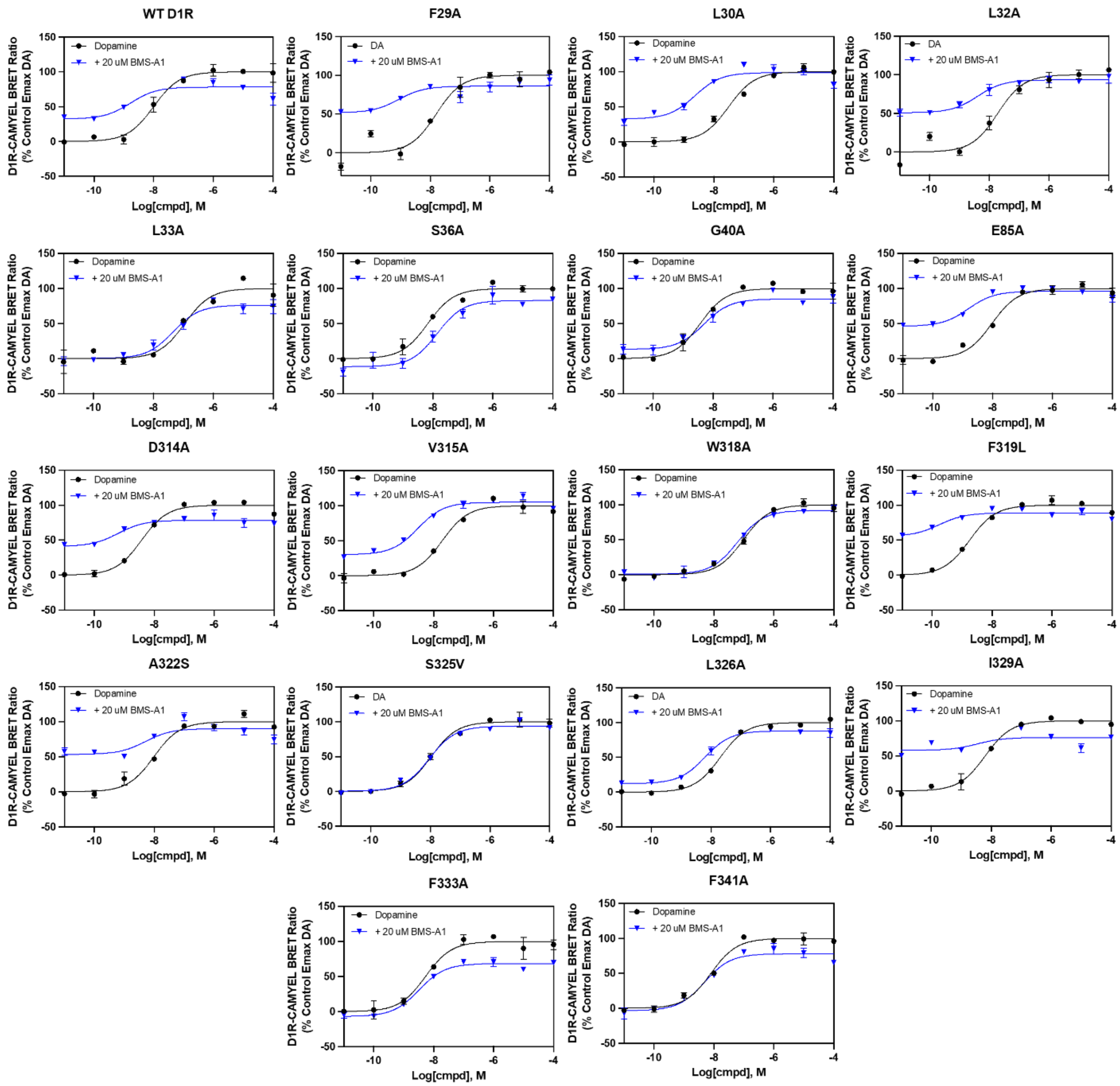

Fig. S9.

Effects of mutating D1R residues on BMS-A1 PAM activity using CAMYEL BRET cAMP assays. The CAMYEL BRET cAMP assay was conducted as described in the Methods Section. DA concentration-response curves in the presence or absence of 20 µM BMS-A1 are shown for WT and each mutant D1R. The data are displayed as a percentage of the maximum DA control response in each experiment and are representative of ≥ 3 independent experiments each performed in duplicate. The EC_{50} fold-shift produced by BMS-A1 is significantly reduced by mutations L33A, S36A, G40A, W318A, S325V, L326A, F333A, and F341A. See **Table S8** for complete curve parameters and statistical analyses.

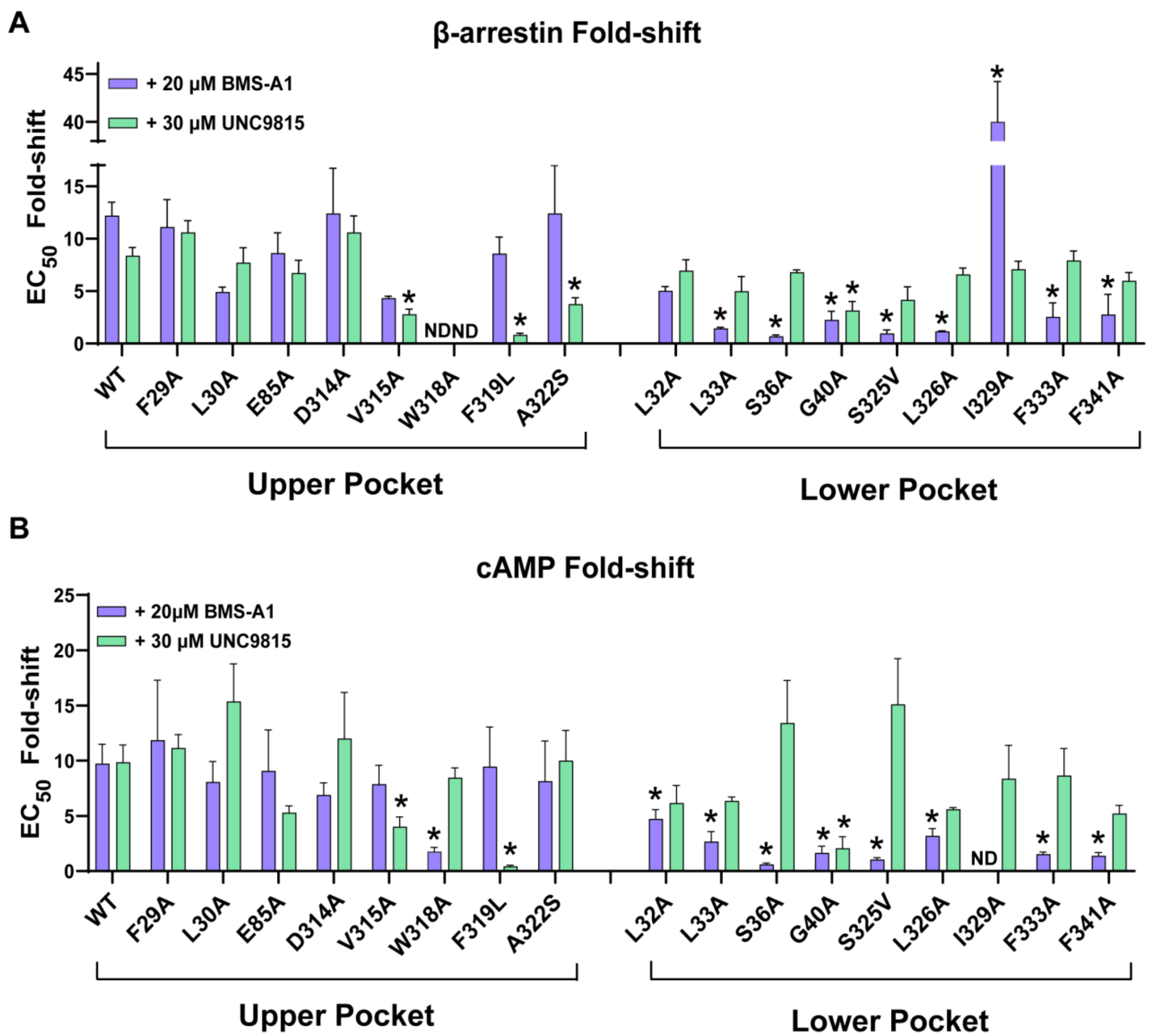

Fig. S10.
Effects of mutations of the allosteric binding pocket residues of BMS-A1 and UNC9815.
Related to **Fig. 2F.**
(A) EC50 fold-shifts induced by BMS-A1 and UNC9815 in the β-arrestin BRET assays shown in **figs. S6** and **S7** (CRCs), and **Tables S5** and **S6** (CRC parameters). Asterisks indicate EC50 fold-shift values observed with the mutant D1Rs that significantly differ from the WT D1R EC50 fold-shift values as shown in **Tables S5** and **S6**. ND = EC50 not determinable.
(B) EC50 fold-shifts induced by BMS-A1 and UNC9815 in the CAMYEL cAMP BRET assays shown in **figs. S8** and **S9** (CRCs), and **Tables S7** and **S8** (CRC parameters). Asterisks indicate EC50 fold-shift values observed with the mutant D1Rs that significantly differ from the WT D1R EC50 fold-shift values as shown in **Tables S7** and **S8**. ND = EC50 not determinable.

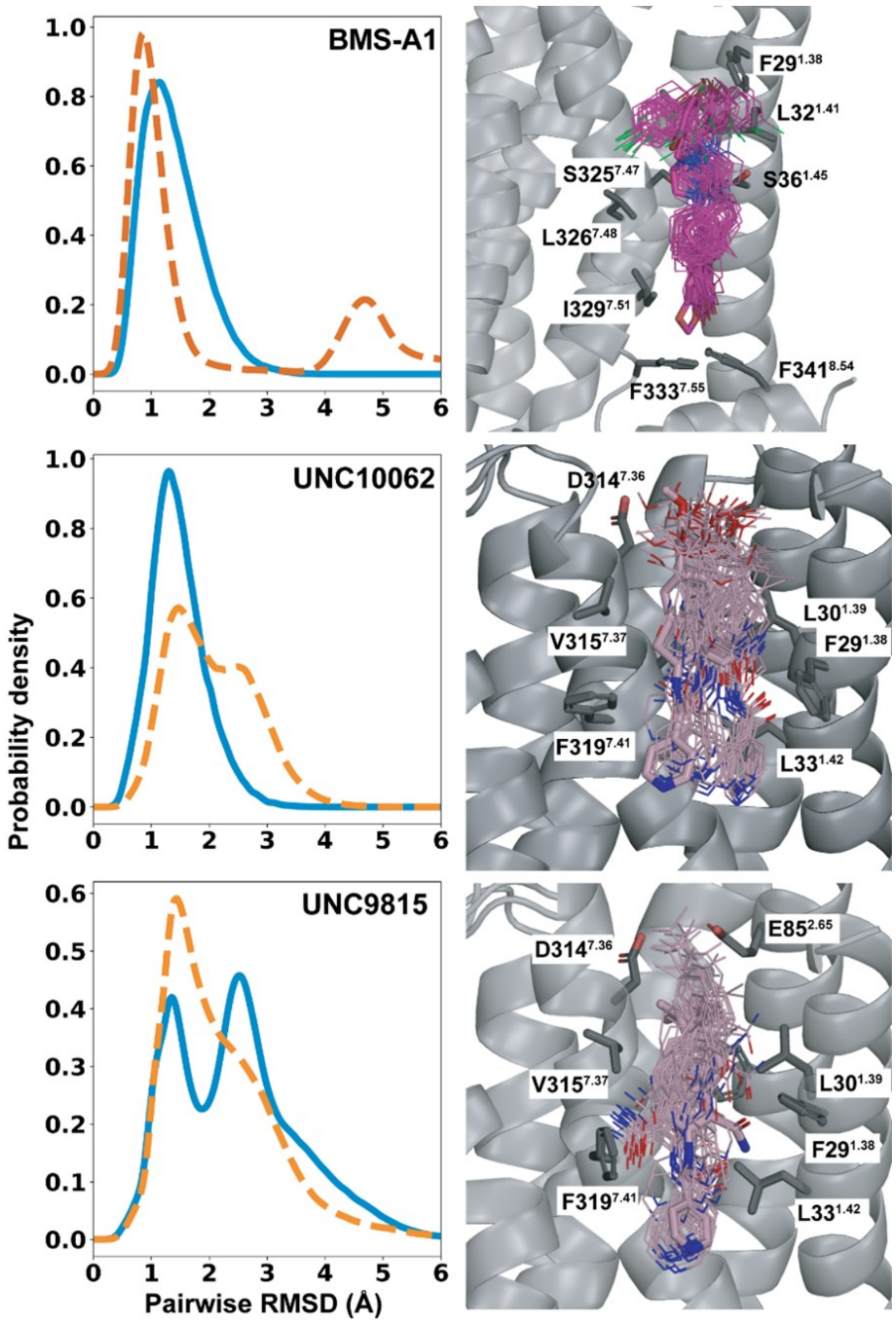

Fig. S11.
Conformational stability of PAMs in the lower and upper pockets in the presence of LY3154207. Left panels show ligand pairwise root-mean-square deviation (RMSD) distributions of BMS-A1, UNC10062, and UNC9815 under different binding conditions. Kernel density estimates of RMSD values were calculated from multiple MD trajectories to evaluate ligand conformational stability. Solid blue lines represent simulations where a PAM was bound together with another PAM at the complementary site (with LY3154207 also present) (e.g., BMS-A1 in the lower pocket with either UNC10062 or UNC9815 in the upper pocket), whereas dashed orange lines represent simulations in which each PAM was bound alone but with LY3154207 present (e.g., BMS-A1 in the lower pocket without any compound in the upper site). Right panels show representative ligand conformations from the co-bound simulations, with cryo-EM poses displayed as sticks and MD-sampled poses as lines (magenta for BMS-A1 and light pink for UNC compounds). Together, these results demonstrate that PAMs bound in the lower and upper pockets exhibit substantial conformational stability.

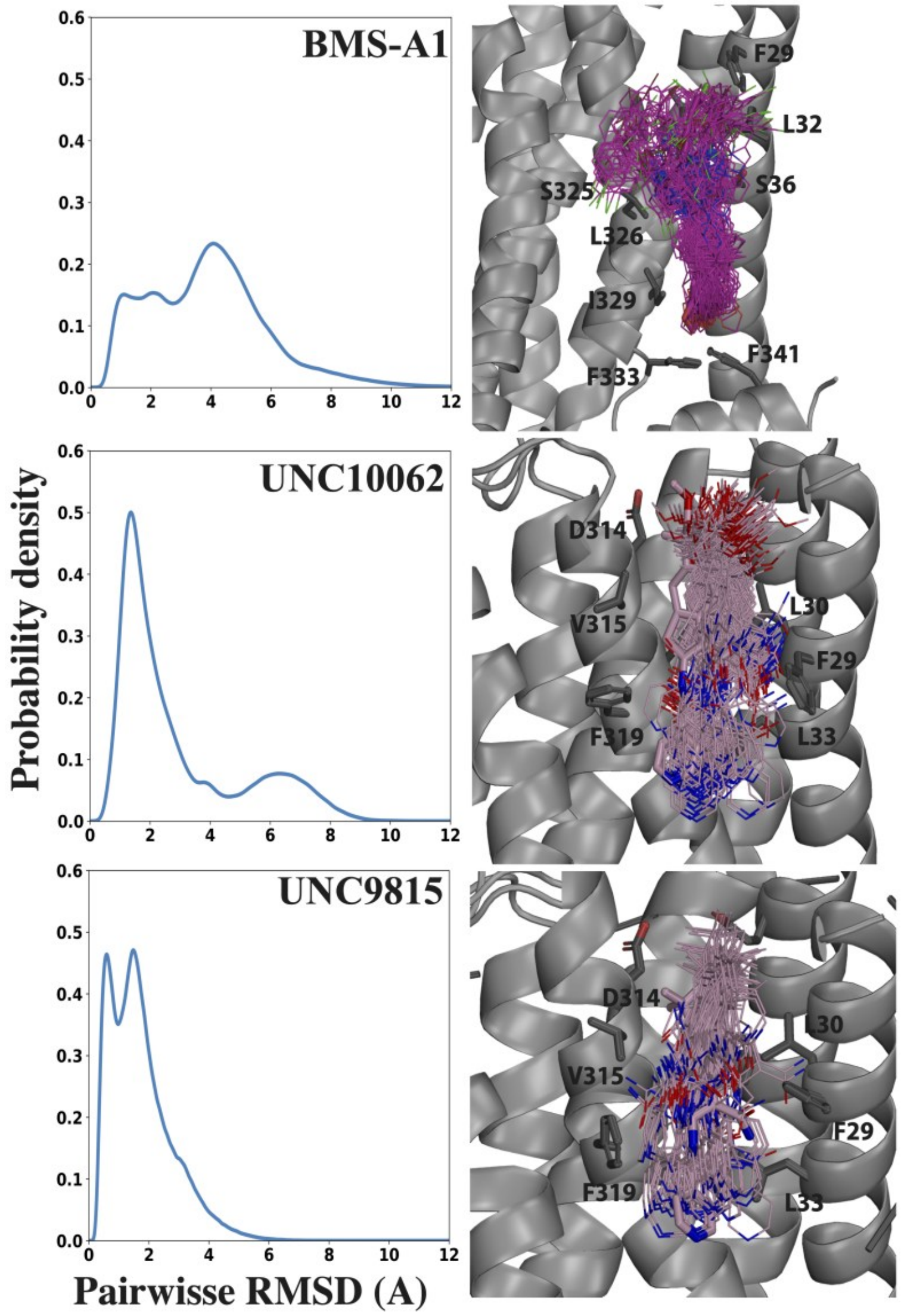

Figure S12.

Conformational stability of BMS-A1, UNC10062, and UNC9815 in the absence of LY3154207. Left panels show ligand pairwise root-mean-square deviation (RMSD) distributions for BMS-A1, UNC10062, and UNC9815 from MD simulations in which each PAM was simulated independently without LY3154207 or the complementary TM1–TM7 PAM. Kernel density estimates of pairwise RMSD values were calculated from MD trajectories to evaluate the conformational stability of each ligand within its assigned allosteric pocket. Right panels show representative ligand conformations sampled during the simulations, with the receptor shown in gray cartoon representation and key pocket-lining residues shown as sticks. BMS-A1 remains localized within the lower TM1–TM7 pocket, whereas UNC10062 and UNC9815 remain localized within the upper TM1–TM7 pocket. Although the ligands exhibit local conformational fluctuations in the absence of LY3154207, particularly within these hydrophobic TM1–TM7 pockets, no ligand dissociation was observed. These results indicate that LY3154207 is not required for stable occupancy of the BMS-A1 or UNC-compound binding pockets, although its presence may enhance overall conformational stability.

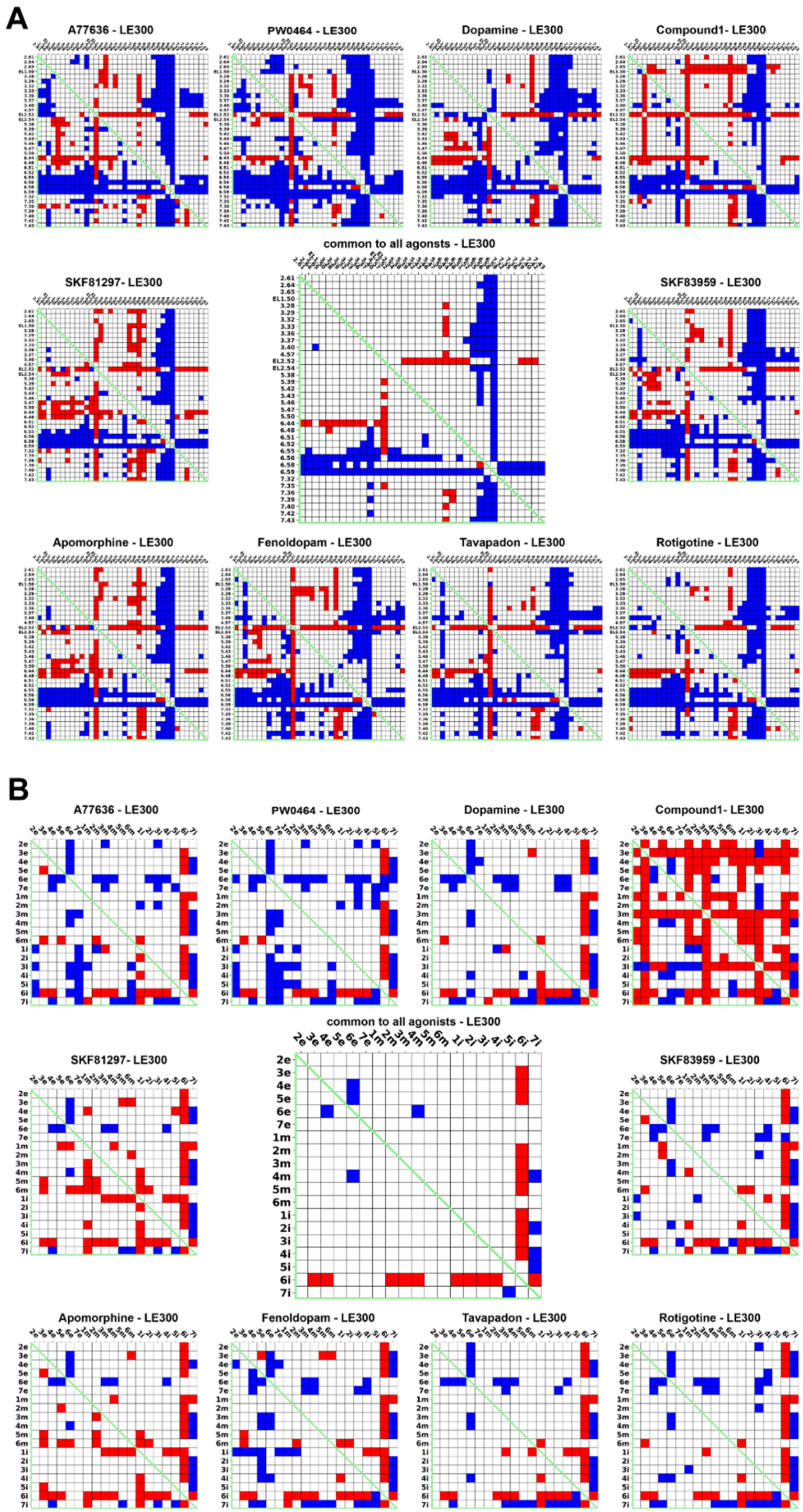
A
A77636 - LE300
PW0464 - LE300
Dopamine - LE300
Compound1- LE300
common to all agonsts - LE300
SKF81297- LE300
SKF83959 - LE300
Apomorphine - LE300
Fenoldopam - LE300
Tavapadon - LE300
Rotigotine - LE300
B
A77636 - LE300
PW0464 - LE300
Dopamine - LE300
Compound1- LE300
common to all agonists - LE300
SKF81297- LE300
SKF83959 - LE300
Apomorphine - LE300
Fenoldopam - LE300
Tavapadon - LE300
Rotigotine - LE300

Fig. S13.

Distance-difference maps comparing active and inactive conformations of D1R. (A) The 10 peripheral panels show pairwise distance-difference matrices between binding site residues of the D1R inactive state (LE300-bound) and the active states stabilized by 10 agonists: A77636 (PDB: 7CKX), PW0464 (7CKY), SKF81297 (7JV5), SKF83959 (7JVP), apomorphine (7JVQ), Compound 1 (7JOZ), dopamine (7F1O), fenoldopam (7X2C), rotigotine (8IRR), and tavapadon (7X2D). Residue pairs with distance changes greater than 1.0 Å are represented in blue and red, indicating that these residue pairs move closer together or further apart, respectively, in the active state. The top-right and bottom-left halves of each panel are based on the calculations of the Cα atoms and sidechain center of mass, respectively. The binding-site residues are labeled by their Ballesteros-Weinstein indices (*91*). The central shows the distance changes common to all 10 agonist-bound states relative to the inactive state. **(B)** Distance-difference matrices of D1R subsegments for the same 10 active-inactive state comparisons and the identification of the common changes, arranged in the same order as **(A)**. The cutoff for distance difference is 1.0 Å. See **Materials and Methods** for the definitions of the subsegments for each TM. Consistent with our previous studies across aminergic GPCRs (*48*), in the D1R active state, $F281^{6.44}$ moved away from the center of the OBP **(A)**, facilitating the outward displacement of TM6i and inward movement of TM7i **(B)**, a hallmark of GPCR activation triggered by the downward movement of $W^{6.48}$ upon agonist binding. Additionally, in the active state, the TM6e (residues 6.55-6.59) moved towards, while $S188^{EL2.52}$ in ECL2 shifted away from other binding-site residues.

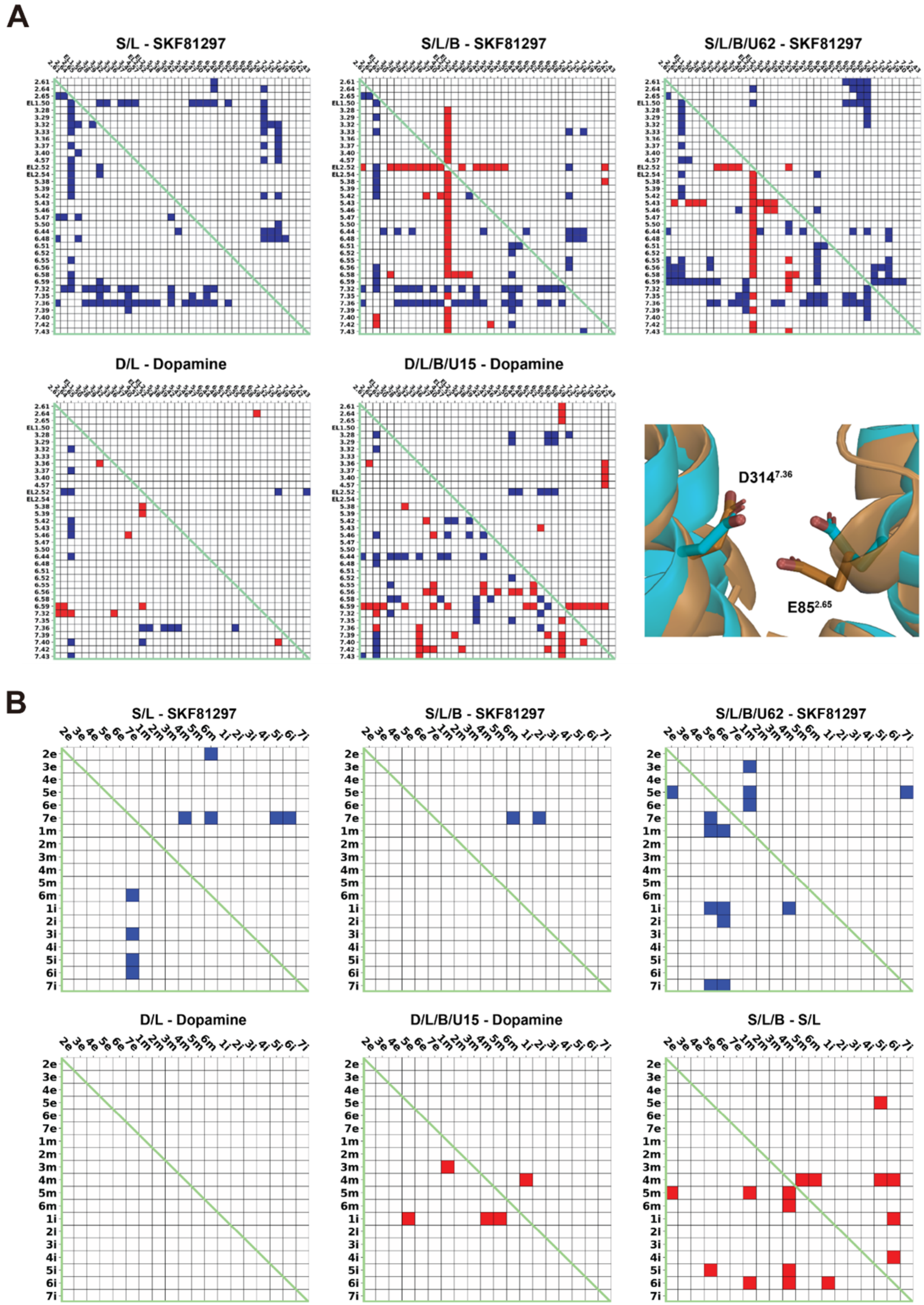
A
S/L - SKF81297
S/L/B - SKF81297
S/L/B/U62 - SKF81297
D/L - Dopamine
D/L/B/U15 - Dopamine
D314^7.36
E85^2.65
B
S/L - SKF81297
S/L/B - SKF81297
S/L/B/U62 - SKF81297
D/L - Dopamine
D/L/B/U15 - Dopamine
S/L/B - S/L

Fig. S14.

Distance difference maps comparing the PAM-bound and unbound active conformations of D1R.

The distance differences of **(A)** binding site residues and **(B)** subsegments are shown. Top-row panels compare the PAM-bound and unbound states in the presence of SKF81297, and bottom-row panels compare those with dopamine. For simplicity, we used the following codes for the ligands in the PAM-bound structures: S = SKF81297, L = LY3154207, U62 = UNC10062, U15 = UNC9815, B = BMS-A1, and D = dopamine. The color scheme and representations follow **fig. S13**. The bottom-right panel shows a representative model at 1200 ns from MD simulations of D1R-SKF81297 (cyan) and D1R-SKF81297/LY3154207 (orange) systems, illustrating E85$^{2.65}$ moving towards the OBP center in PAM-bound structures.

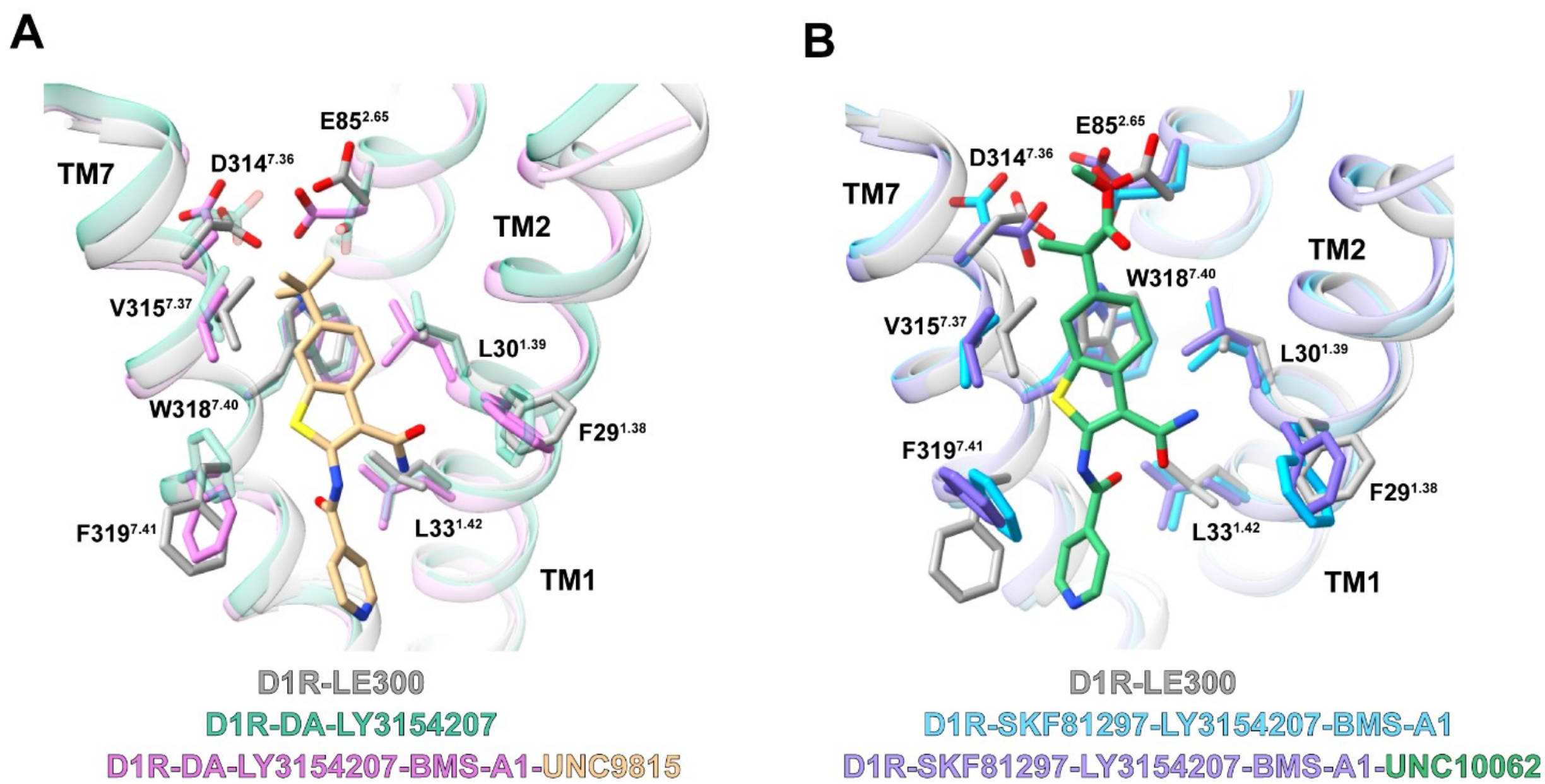

Fig. S15.

Structural comparison of the UNC compound binding pockets at D1R in different ligand-binding states. (A) Structural overlay of D1R highlights that UNC9815 stabilizes the active state of the extracellular end of TM7 through binding at the upper allosteric site. **(B)** UNC10062 binding to the upper allosteric site reinforces the active conformation of TM7's extracellular region.

Table S1.

Dose-response relationships for D1R PAM potentiation of DA-stimulated β-arrestin recruitment. The EC_{50} values were determined from dopamine concentration-response curves as described in **fig. S2**. Data represent mean ± SEM values with the number of experiments (*n*) provided in parentheses. The EC_{50} fold-shift values were derived by dividing the EC_{50} of the DA control curve by the EC_{50} of the DA curve in the presence of PAMs for each individual experiment. The calculated fold-shifts for each experiment were then averaged. Statistical comparisons were made between the control DA EC_{50} values versus the DA + PAM EC_{50} values using a one-way ANOVA with Dunnett's post hoc comparison: * $p < 0.05$, ** $p < 0.01$, *** $p < 0.001$, **** $p < 0.0001$. ND = not determined.

**Condition**	**+ UNC9815** EC_{50} (nM) (Fold-shift)	**+ UNC10062** EC_{50} (nM) (Fold-shift)	**+ BMS-A1** EC_{50} (nM) (Fold-shift)	**+ LY3154207** EC_{50} (nM) (Fold-shift)
**DA**	3,500 ± 1100 (*5*)	1,300 ± 270 (*6*)	1,000 ± 170 (*5*)	950 ± 290 (*6*)
**+ 0.3 µM**	ND	ND	ND	92 ± 53** (*3*) (13.4 ± 1.6)
**+ 1 µM**	3,000 ± 960 (*5*) (1.4 ± 0.3)	1,300 ± 380 (*6*) (1.2 ± 0.2)	1,100 ± 270 (*4*) (1.3 ± 0.5)	130 ± 49** (*4*) (10.5 ± 1.1)
**+ 3 µM**	1,400 ± 500** (*5*) (2.8 ± 0.3)	1,000 ± 240* (*6*) (1.3 ± 0.1)	810 ± 180 (*4*) (1.6 ± 0.3)	96 ± 34** (*4*) (13.4 ± 1.4)
**+ 10 µM**	640 ± 270** (*5*) (8.4 ± 2.4)	540 ± 130** (*6*) (2.7 ± 0.3)	190 ± 47** (*4*) (6.9 ± 1.3)	87 ± 37** (*4*) (15.8 ± 2.4)
**+ 20 µM**	230 ± 74*** (*5*) (17.6 ± 3.2)	340 ± 80*** (*5*) (4.0 ± 0.3)	180 ± 21** (*4*) (6.3 ± 0.4)	73 ± 28** (*4*) (19.3 ± 3.0)
**+ 25 µM**	190 ± 55**** (*5*) (18.6 ± 2.7)	ND	160 ± 42* (*4*) (8.6 ± 2.0)	ND
**+ 30 µM**	210 ± 64**** (*5*) (17.1 ± 2.2)	280 ± 53**** (*6*) (4.5 ± 0.4)	130 ± 41** (*5*) (11.4 ± 2.7)	84 ± 37** (*4*) (16.5 ± 2.0)
**+ 50 µM**	ND	340 ± 70*** (*6*) (3.9 ± 0.5)	ND	51 ± 29**** (*6*) (27.2 ± 4.6)
**+ 100 µM**	ND	ND	ND	46 ± 28** (*3*) (20.8 ± 2.6)

Table S2.
Dose-response relationships for D1R PAM potentiation of β-arrestin recruitment stimulated by DA and SKF81297. Curve parameters were determined as described in **Figs. 1B-C**. Data represent mean ± SEM values with the number of experiments (*n*) provided in parentheses. The EC_{50} fold-shift and Emax values are provided for informational purposes. The fold-shifts were derived by dividing the EC_{50} of the DA control curve by the EC_{50} of the DA curve in the presence of PAMs for each experiment. The % Emax values represent the percent of the control DA Emax values in each experiment. Differences between the EC_{50} values were evaluated for statistical significance using a one-way ANOVA with Šídák's multiple comparisons test: * $p < 0.05$ for single PAMs vs. DA or SKF81297 alone, † $p < 0.05$ for double PAM combinations vs. the relevant single PAMs, and ‡ $p < 0.05$ for triple PAM combinations vs. the relevant double PAM combinations with the following exceptions: $p > 0.05$ for DA or SKF81297 + LY3154207 + UNC10062 vs. DA or SKF81297 + LY3154207; $p > 0.05$ for DA or SKF81297 + UNC10062 + UNC9815 vs DA or SKF81297 + UNC10062; $p > 0.05$ for DA or SKF81297 + UNC9815 + UNC10062 vs. DA + UNC9815 or DA vs. UNC10062.

Condition	EC_{50} (nM)	EC_{50} Fold-Shift	Emax (% DA Max)
**DA**	1,450 ± 218 (*12*)	1 (*12*)	100 (*12*)
**+ 30 µM UNC9815**	46.6 ± 10.8* (*12*)	39.7 ± 6.77 (*12*)	114 ± 4.6 (*12*)
**+ 20 µM BMS-A1**	125 ± 32.5* (*12*)	18.6 ± 5.38 (*12*)	92 ± 2.5 (*12*)
**+ 50 µM UNC10062**	267 ± 62.8* (*9*)	6.59 ± 0.92 (*9*)	164 ± 12 (*9*)
**+ 50 µM LY3154207**	64.2 ± 14.9* (*12*)	26.7 ± 2.09 (*12*)	105 ± 3.4 (*12*)
**+ BMS-A1 + UNC9815**	6.76 ± 1.54† (*12*)	365 ± 118 (*12*)	119 ± 7.9 (*12*)
**+ BMS-A1 + UNC10062**	18.8 ± 5.40† (*9*)	123 ± 36.1 (*9*)	146 ± 12 (*9*)
**+ BMS-A1 + LY3154207**	10.9 ± 2.00† (*11*)	228 ± 79.0 (*11*)	123 ± 5.7 (*11*)
**+ LY3154207 + UNC10062**	40.3 ± 10.1 (*9*)	38.9 ± 4.58 (*9*)	146 ± 17 (*9*)
**+ LY3154207 + UNC9815**	12.4 ± 1.91† (*12*)	139 ± 26.1 (*12*)	153 ± 12 (*12*)
**+ UNC10062 + UNC9815**	249 ± 87.0 (*9*)	9.07 ± 1.56 (*9*)	110 ± 14 (*9*)
**+ LY3154207 + BMS-A1 + UNC10062**	4.99 ± 1.54‡ (*9*)	469 ± 113 (*9*)	119 ± 16 (*9*)
**+ LY3154207 + BMS-A1 + UNC9815**	2.42 ± 0.62‡ (*12*)	1,090 ± 261 (*12*)	133 ± 9.6 (*12*)
**SKF81297**	31.7 ± 9.67 (*8*)	1 (*8*)	53 ± 1.4 (*8*)
**+ 30 µM UNC9815**	4.48 ± 1.48* (*6*)	8.3 ± 1.1 (*6*)	117 ± 7.7 (*6*)
**+ 20 µM BMS-A1**	5.29 ± 1.74* (*8*)	6.6 ± 0.7 (*8*)	91 ± 4.4 (*8*)
**+ 50 µM UNC10062**	5.76 ± 1.68* (*8*)	5.8 ± 0.9 (*8*)	138 ± 12 (*8*)
**+ 50 µM LY3154207**	4.70 ± 1.95**** (*8*)	9.6 ± 1.7 (*8*)	104 ± 2.9 (*8*)
**+ BMS-A1 + UNC9815**	0.74 ± 0.33† (*6*)	65 ± 14 (*6*)	117 ± 4.8 (*6*)
**+ BMS-A1 + UNC10062**	0.97 ± 0.35† (*7*)	43 ± 8.0 (*7*)	146 ± 11 (*7*)

**+ BMS-A1 + LY3154207**	1.77 ± 0.93† (*8*)	53 ± 8.1 (*8*)	116 ± 3.1 (*8*)
**+ LY3154207 + UNC10062**	1.74 ± 0.55† (*8*)	20 ± 1.2 (*8*)	146 ± 14 (*8*)
**+ LY3154207 + UNC9815**	0.70 ± 0.23† (*8*)	53 ± 8.1 (*8*)	131 ± 10 (*8*)
**+ UNC10062 + UNC9815**	5.00 ± 1.36 (*8*)	7.4 ± 1.3 (*8*)	73 ± 10 (*8*)
**+ LY3154207 + BMS-A1 + UNC10062**	0.84 ± 0.36 (*7*)	53 ± 10 (*7*)	116 ± 12 (*7*)
**+ LY3154207 + BMS-A1 + UNC9815**	0.70 ± 0.32 (*6*)	68 ± 17 (*6*)	122 ± 9.5 (*6*)

Table S3.
Dose-response relationships for D1R PAM potentiation of G protein dissociation stimulated by DA.
The EC_{50} values were determined from dopamine concentration-response curves as described in **fig. S3**. Data represent mean ± SEM values from at least 3 independent experiments, each performed in triplicate. The EC_{50} fold-shift values were derived by dividing the EC_{50} of the DA control curve by the EC_{50} of the DA curve in the presence of PAMs for each individual experiment. The calculated fold-shifts for each experiment were then averaged. Statistical comparisons were made between the control DA EC_{50} values versus the DA + PAM EC_{50} values using a one-way ANOVA with Dunnett's post hoc comparison: * $p < 0.05$ for single PAMs vs. DA alone, †$p < 0.05$ for double PAM combinations vs. the relevant single PAMs.

Condition	EC_{50} (nM)	EC_{50} Fold-Shift	Emax (% DA Max)
**DA**	59.3 ± 9.70	1	100
**+ 30 µM UNC9815**	4.76 ± 0.85*	16.8 ± 1.83	98.6 ± 1.46
**+ 15 µM BMS-A1**	11.6 ± 1.40*	6.99 ± 0.83	102 ± 3.51
**+ 50 µM UNC10062**	5.28 ± 1.66*	15 ± 3.75	132 ± 2.13
**+ 30 µM LY3154207**	4.82 ± 1.47*	22 ± 5.01	111 ± 4.08
**+ BMS-A1 + UNC9815**	1.08 ± 0.54†	92.9 ± 20.3	104 ± 1.89
**+ BMS-A1 + UNC10062**	1.15 ± 0.29†	78 ± 14.4	131 ± 7.30
**+ BMS-A1 + LY3154207**	0.95 ± 0.09†	82.3 ± 9.14	132 ± 4.16
**+ LY3154207 + UNC10062**	2.30 ± 0.39†	38.9 ± 6.21	119 ± 3.86
**+ LY3154207 + UNC9815**	1.87 ± 0.36†	51.1 ± 8.25	109 ± 3.27
**+ UNC10062 + UNC9815**	9.21 ± 0.92	8.69 ± 0.88	111 ± 4.21
**+ LY3154207 + BMS-A1 + UNC10062**	0.96 ± 0.01	82 ± 0.46	150 ± 10.2
**+ LY3154207 + BMS-A1 + UNC9815**	0.68 ± 0.04	111 ± 12.2	133 ± 9.28

Table S4.
Cryo-EM data collection, structural model refinement and validation statistics of D1R bound to distinct PAMs. Related to **Figs. 1E-G, 2A-E** and **fig. S4**.

	**D1R-miniG$_s$-Nb35**		
	SKF81297/LY3154207/ BMS-A1	SKF81297/LY3154207/ BMS-A1/UNC10062	DA/LY3154207/ BMS-A1/UNC9815
**Data processing**			
Magnification	105K	165K	165K
Voltage (kV)	300	300	300
Electron exposure (e-/Å^2)	50	50	50
Defocus range (μm)	-1.0~-3.0	-0.8~-1.8	-0.8~-1.8
Pixel size (Å)	0.824	0.73	0.73
Symmetry imposed	C1	C1	C1
initial particle images(no.)	1,098,429	246,437	390,276
Final particle images(no.)	898,739	201,601	209,112
Map resolution (Å)	2.65	2.59	2.44
FSC threshold	0.143	0.143	0.143
**Refinement**			
Map resolution (Å)	2.6	2.6	2.4
FSC threshold	0.143	0.143	0.143
Model-Map CC (mask)	0.86	0.74	0.87
Model composition			
Non-hydrogen atoms	8399	8335	8342
Protein residues	1040	1038	1038
B factors (Å^2)	117.1	97.7	84.3
Protein	19.80/116.22/43.35	7.90/135.61/39.02	9.12/94.95/33.52
Ligand	27.84/66.94/50.48	15.42/20.42/18.51	20.00/47.90/29.65
Water	-/-/-	-/-/-	34.36/34.36/34.36
R.m.s. deviations			
Bond lengths (Å)	0.026	0.007	0.003
Bond angles (°)	1.004	0.716	0.946
Validation			
MolProbity score	1.48	1.58	1.40
Clash score	6.52	6.73	5.44
Rotamer outliers (%)	0.00	0.00	0.00
Ramachandran plot			
Favored (%)	97.36	96.67	97.46
Allowed (%)	2.64	3.33	2.54
Disallowed (%)	0	0	0

Table S5.

Effects of mutating D1R residues on UNC9815 activity using β-arrestin recruitment assays. Curve parameters were determined as described in **fig. S6**. Data represent mean ± SEM values with the number of experiments (*n*) provided in parentheses. Fold-shift values were derived by dividing the EC_{50} of the DA control curve by the EC_{50} of the DA curve in the presence of PAMs for each experiment and then averaged. The average Emax values represent the percent of the control DA Emax value and are shown for informational purposes. For each mutant D1R, the EC_{50} value in the presence of the PAM was compared to the EC_{50} value in the absence of the PAM using a *t*-test. Similarly, the EC_{50} fold-shift for each mutant was statistically compared to the EC_{50} fold-shift for the WT D1R using a *t*-test and reported in **Fig. 2F**.* $p < 0.05$, ** $p < 0.01$, *** $p < 0.001$, **** $p < 0.0001$. ND = EC_{50} not determinable.

		**DA**	**DA + 30 µM UNC9815**		
	**D1R Construct**	**EC_{50} (µM)**	**EC_{50} (µM)**	**EC_{50} Fold-Shift**	**Emax (% DA Emax)**
	**WT**	4.73 ± 0.95 (*26*)	0.57 ± 0.07**** (*26*)	8.38 ± 0.79 (*26*)	135 ± 4.11% (*26*)
**Upper Binding Site**	**F29A**	3.43 ± 0.60 (*4*)	0.32 ± 0.03*** (*4*)	10.6 ± 1.12 (4)	165 ± 13.3% (*4*)
	**L30A**	10.6 ± 0.46 (*3*)	1.44 ± 0.19** (*3*)	7.73 ± 1.41 (*3*)	187 ± 6.12% (*3*)
	**E85A**	7.62 ± 2.12 (*5*)	1.17 ± 0.31*** (*5*)	6.72 ± 1.22 (*5*)	133 ± 5.76% (*5*)
	**D314A**	7.45 ± 1.53 (*4*)	0.79 ± 0.24*** (*4*)	10.6 ± 1.58 (*4*)	150 ± 9.01% (*4*)
	**V315A**	11.5 ± 2.40 (*6*)	4.47 ± 1.10** (*6*)	2.80 ± 0.48* (*6*)	180 ± 15.5% (*6*)
	**W318A**	ND (*3*)	4.90 ± 0.72 (*3*)	ND (*3*)	163 ± 39.0% (*3*)
	**F319L**	2.76 ± 0.80 (*4*)	3.19 ± 0.79 (*4*)	0.83 ± 0.15** (*4*)	124 ± 3.14% (4)
	**A322S**	6.32 ± 1.28 (*6*)	1.83 ± 0.41** (*6*)	3.76 ± 0.61* (*6*)	143 ± 11.5% (*6*)
**Lower Binding Site**	**L32A**	2.52 ± 0.08 (*4*)	0.39 ± 0.05*** (*4*)	6.96 ± 1.04 (*4*)	121 ± 0.57% (*4*)
	**L33A**	10.7 ± 1.39 (*4*)	2.63 ± 0.70* (*4*)	4.98 ± 1.40 (*4*)	144 ± 10.0% (*4*)
	**S36A**	2.59 ± 0.27 (*4*)	0.38 ± 0.04**** (*4*)	6.80 ± 0.22 (*4*)	119 ± 1.16% (*4*)

	**G40A**	0.13 ± 0.02 (*4*)	0.05 ± 0.02* (*4*)	3.16 ± 0.86**** (*4*)	131 ± 11.0% (*4*)
	**S325V**	2.55 ± 0.87 (*6*)	0.60 ± 0.04* (*6*)	4.16 ± 1.25 (*6*)	311 ± 34.3% (*6*)
	**L326A**	2.27 ± 0.11 (*3*)	0.35 ± 0.02** (*3*)	6.59 ± 0.62 (*3*)	113 ± 0.65% (*3*)
	**I329A**	1.27 ± 0.15 (*4*)	0.18 ± 0.02*** (*4*)	7.08 ± 0.78 (*4*)	125 ± 2.13% (*4*)
	**F333A**	1.27 ± 0.22 (*4*)	0.17 ± 0.04*** (*4*)	7.93 ± 0.89 (*4*)	113 ± 3.64% (*4*)
	**F341A**	0.32 ± 0.07 (*4*)	0.05 ± 0.009** (*4*)	5.97 ± 0.81 (*4*)	118 ± 3.61% (*4*)

Table S6.
Effects of mutating D1R residues on BMS-A1 activity using β-arrestin recruitment assays. Curve parameters were determined as described in **fig. S7**. Data represent mean ± SEM values with the number of experiments (*n*) provided in parentheses. Fold-shift values were derived by dividing the EC_{50} of the DA control curve by the EC_{50} of the DA curve in the presence of PAMs for each experiment and then averaged. The average Emax values represent the percent of the control DA Emax value and are shown for informational purposes. For each mutant D1R, the EC_{50} value in the presence of the PAM was compared to the EC_{50} value in the absence of the PAM using a *t*-test. Similarly, the EC_{50} fold-shift for each mutant was statistically compared to the EC_{50} fold-shift for the WT D1R using a *t*-test and reported in **Fig. 2F**.* $p < 0.05$, ** $p < 0.01$, *** $p < 0.001$, **** $p < 0.0001$. ND = EC_{50} not determinable.

		**DA**	**DA + 20 µM BMS-A1**		
	**D1R Construct**	**EC50 (µM)**	**EC50 (µM)**	**EC50 Fold-Shift**	**Emax (%, DA Emax)**
	**WT**	3.03 ± 0.32 (*17*)	0.29 ± 0.04**** (*17*)	12.2 ± 1.28 (*17*)	145 ± 10.3% (*17*)
**Upper Binding Site**	**F29A**	0.91 ± 0.17 (*4*)	0.10 ± 0.03** (*4*)	11.1 ± 2.64 (*4*)	111 ± 4.09% (*4*)
	**L30A**	9.40 ± 0.61 (*3*)	1.97 ± 0.29** (*3*)	4.91 ± 0.48 (*3*)	194 ± 40.8% (*3*)
	**E85A**	2.97 ± 0.53 (*4*)	0.38 ± 0.07** (*4*)	8.63 ± 1.95 (*4*)	108 ± 6.35% (*4*)
	**D314A**	2.27 ± 0.35 (*3*)	0.22 ± 0.05* (*3*)	12.4 ± 4.32 (*3*)	111 ± 6.79% (*3*)
	**V315A**	7.23 ± 1.24 (*3*)	1.67 ± 0.30*** (*3*)	4.34 ± 0.16 (*3*)	159 ± 11.2% (*3*)
	**W318A**	ND (*3*)	ND (*3*)	ND (*3*)	121 ± 22.0% (*3*)
	**F319L**	1.09 ± 0.11 (*3*)	0.13 ± 0.02** (*3*)	8.57 ± 1.59 (*3*)	114 ± 5.95% (*3*)
	**A322S**	3.05 ± 0.36 (*5*)	0.38 ± 0.13** (*5*)	12.4 ± 4.58 (*5*)	111 ± 4.87% (*5*)
**Lower Binding Site**	**L32A**	2.25 ± 0.34 (*4*)	0.47 ± 0.09*** (*4*)	5.03 ± 0.41 (*4*)	110 ± 12.5% (*4*)
	**L33A**	6.77 ± 1.80 (*5*)	4.79 ± 1.37** (*5*)	1.44 ± 0.11* (*5*)	122 ± 26.1% (*5*)
	**S36A**	2.18 ± 0.42 (*5*)	3.50 ± 0.78 (*5*)	0.68 ± 0.14* (*5*)	107 ± 13.3% (*5*)
	**G40A**	0.24 ± 0.10 (*6*)	0.09 ± 0.02 (*6*)	2.25 ± 0.84* (*6*)	82.4 ± 11.6% (*6*)
	**S325V**	2.41 ± 0.76 (*3*)	2.73 ± 0.74 (*3*)	0.97 ± 0.32* (*3*)	180 ± 24.9% (*3*)
	**L326A**	1.27 ± 0.17 (*3*)	1.09 ± 0.14 (*3*)	1.16 ± 0.04* (*3*)	106 ± 3.76% (*3*)
	**I329A**	1.13 ± 0.22 (*4*)	0.03 ± 0.005**** (*4*)	40.0 ± 4.21* (*4*)	109 ± 13.9% (*4*)
	**F333A**	1.42 ± 0.15 (*4*)	0.99 ± 0.33 (*4*)	2.55 ± 1.33* (*4*)	99.8 ± 16.3% (*4*)
	**F341A**	0.51 ± 0.15 (*4*)	0.37 ± 0.11 (*4*)	2.78 ± 1.92* (*4*)	96.3 ± 7.97% (*4*)

Table S7.
Effects of mutating D1R residues on UNC9815 activity using CAMYEL BRET cAMP assays. Curve parameters were determined as described in **fig. S8**. Data represent mean ± SEM values with the number of experiments (*n*) provided in parentheses. Fold-shift values were derived by dividing the EC_{50} of the DA control curve by the EC_{50} of the DA curve in the presence of PAMs for each experiment and then averaged. The average Emax values represent the percent of the control DA Emax value and are shown for informational purposes. For each mutant D1R, the EC_{50} value in the presence of the PAM was compared to the EC_{50} value in the absence of the PAM using a *t*-test. Similarly, the EC_{50} fold-shift for each mutant was statistically compared to the EC_{50} fold-shift for the WT D1R using a *t*-test and reported in **Fig. 2F**.* $p < 0.05$, ** $p < 0.01$, *** $p < 0.001$, **** $p < 0.0001$. ND = EC_{50} not determinable.

		DA	DA + 30 µM UNC9815		
	**D1R Construct**	**EC_{50} (nM)**	**EC_{50} (nM)**	**EC_{50} Fold Shift**	**Baseline Agonism (% DA Emax)**
	**WT D1R**	11.5 ± 1.97 (*12*)	1.34 ± 0.24**** (*12*)	9.85 ± 1.57 (*12*)	40.5 ± 4.03% (*12*)
**Upper Binding Site**	**F29A**	6.73 ± 1.22 (*3*)	0.61 ± 0.13** (*3*)	11.2 ± 1.19 (*3*)	58.1 ± 7.84% (*3*)
	**L30A**	51.7 ± 14.9 (*4*)	3.30 ± 0.45** (*4*)	15.4 ± 3.42 (*4*)	14.8 ± 3.63% (*4*)
	**E85A**	8.89 ± 0.86 (*3*)	1.73 ± 0.30** (*3*)	5.30 ± 0.62 (*3*)	34.0 ± 4.74% (*3*)
	**D314A**	11.6 ± 1.90 (*5*)	1.51 ± 0.44** (*5*)	12.0 ± 4.19 (*5*)	31.9 ± 4.81% (*5*)
	**V315A**	20.7 ± 2.26 (*5*)	6.04 ± 1.07** (*5*)	4.02 ± 0.89* (*5*)	13.2 ± 4.78% (*5*)
	**W318A**	70.9 ± 6.28 (*3*)	8.62 ± 1.26** (*3*)	8.45 ± 0.89 (*3*)	14.2 ± 10.8% (*3*)
	**F319L**	1.82 ± 0.09 (*3*)	4.75 ± 1.35 (*3*)	0.44 ± 0.10* (*3*)	12.7 ± 15.4% (*3*)
	**A322S**	14.9 ± 2.48 (*4*)	1.70 ± 0.29** (*4*)	10.0 ± 2.75 (*4*)	16.9 ± 8.76% (*4*)
**Lower Binding Site**	**L32A**	9.20 ± 1.84 (*4*)	1.75 ± 0.37* (*4*)	6.17 ± 1.60 (*4*)	46.3 ± 4.53% (*4*)
	**L33A**	91.4 ± 30.6 (*3*)	14.8 ± 5.41*** (*3*)	6.36 ± 0.36 (*3*)	8.89 ± 3.90% (*3*)
	**S36A**	9.36 ± 5.27 (*4*)	0.73 ± 0.28** (*4*)	13.4 ± 3.86 (*4*)	45.2 ± 10.7% (*4*)
	**G40A**	3.80 ± 1.89 (*3*)	1.48 ± 0.42 (*3*)	2.07 ± 1.04* (*3*)	46.4 ± 6.25% (*3*)
	**S325V**	30.3 ± 14.1 (*3*)	2.03 ± 0.81* (*3*)	15.1 ± 4.14 (*3*)	21.9 ± 5.66% (*3*)
	**L326A**	4.53 ± 0.70 (*3*)	0.82 ± 0.14*** (*3*)	5.60 ± 0.17 (*3*)	57.1 ± 9.42% (*3*)
	**I329A**	2.98 ± 0.68 (*3*)	0.45 ± 0.20* (*3*)	8.38 ± 3.00 (*3*)	41.7 ± 12.3% (*3*)
	**F333A**	5.12 ± 2.26 (*4*)	0.59 ± 0.15** (*4*)	8.64 ± 2.47 (*4*)	43.6 ± 3.44% (*4*)
	**F341A**	10.4 ± 4.60 (*4*)	1.95 ± 0.65** (*4*)	5.20 ± 0.75 (*4*)	35.8 ± 10.9% (*4*)

Table S8.

Effects of mutating D1R residues on BMS-A1 activity using CAMYEL BRET cAMP assays. Curve parameters were determined as described in **fig. S9**. Data represent mean ± SEM values with the number of experiments (*n*) provided in parentheses. Fold-shift values were derived by dividing the EC_{50} of the DA control curve by the EC_{50} of the DA curve in the presence of PAMs for each experiment. The calculated fold-shifts for each experiment were then averaged. The average Emax values represent the percent of the control DA Emax value and are shown for informational purposes. For each mutant D1R, the EC_{50} value in the presence of the PAM was compared to the EC_{50} value in the absence of the PAM using a *t*-test. Similarly, the EC_{50} fold-shift for each mutant was statistically compared to the EC_{50} fold-shift for the WT D1R using a *t*-test and reported in **Fig. 2F**.* $p < 0.05$, ** $p < 0.01$, *** $p < 0.001$, **** $p < 0.0001$. ND = EC_{50} not determinable.

		**DA**	**DA + 20 µM BMS-A1**		
	**D1R Construct**	**EC_{50} (nM)**	**EC_{50} (nM)**	**EC_{50} Fold Shift**	**Baseline Agonism (% DA Emax)**
	**WT**	14.2 ± 2.19 (*18*)	1.87 ± 0.30**** (*18*)	9.73 ± 1.76 (*18*)	39.2 ± 2.66% (*18*)
**Upper Binding Site**	**F29A**	9.65 ± 3.08 (*5*)	1.27 ± 0.34* (*5*)	11.9 ± 5.42 (*5*)	47.3 ± 4.38% (*5*)
	**L30A**	35.0 ± 10.7 (*5*)	4.34 ± 0.98** (*5*)	8.08 ± 1.84 (*5*)	20.2 ± 5.30% (*5*)
	**E85A**	18.2 ± 5.70 (*6*)	2.66 ± 0.74** (*6*)	9.07 ± 3.71 (*6*)	35.6 ± 4.80% (*6*)
	**D314A**	18.2 ± 7.03 (*6*)	2.70 ± 0.87*** (*6*)	6.89 ± 1.09 (*6*)	47.1 ± 3.64% (*6*)
	**V315A**	26.8 ± 8.22 (*5*)	3.28 ± 0.58** (*5*)	7.87 ± 1.71 (*5*)	38.3 ± 14.4% (*5*)
	**W318A**	92.9 ± 20.8 (*4*)	51.6 ± 7.39 (*4*)	1.78 ± 0.37* (*4*)	-3.93 ± 2.50% (*4*)
	**F319L**	9.34 ± 3.54 (*6*)	3.17 ± 2.18* (*6*)	9.46 ± 3.59 (*6*)	54.6 ± 6.09% (*6*)
	**A322S**	19.9 ± 7.75 (*5*)	4.44 ± 2.23* (*5*)	8.13 ± 3.66 (*5*)	39.9 ± 15.8% (*5*)
**Lower Binding Site**	**L32A**	13.9 ± 3.97 (*6*)	3.48 ± 1.39*** (*6*)	4.72 ± 0.84* (*6*)	26.2 ± 7.06% (*6*)
	**L33A**	108 ± 26.0 (*4*)	47.8 ± 9.56 (*4*)	2.65 ± 0.93* (*4*)	-0.24 ± 2.83% (*4*)
	**S36A**	7.79 ± 2.01 (*3*)	12.7 ± 1.01 (*3*)	0.60 ± 0.13* (*3*)	-0.95 ± 2.74% (*3*)
	**G40A**	5.81 ± 0.78 (*4*)	5.33 ± 2.00 (*4*)	1.65 ± 0.60* (*4*)	13.3 ± 4.33% (*4*)
	**S325V**	3.55 ± 7.16 (*7*)	40.0 ± 9.08 (*7*)	1.04 ± 0.19* (*7*)	0.10 ± 1.70% (*7*)
	**L326A**	18.0 ± 7.85 (*5*)	5.73 ± 1.57 (*5*)	3.19 ± 0.67* (*5*)	1.69 ± 5.92% (*5*)
	**I329A**	9.49 ± 3.44 (*3*)	ND (*3*)	ND (*3*)	63.1 ± 4.96% (*3*)
	**F333A**	4.04 ± 0.65 (*3*)	2.69 ± 0.46 (*3*)	1.54 ± 0.20* (*3*)	-8.94 ± 8.46% (*3*)
	**F341A**	8.50 ± 0.81 (*3*)	6.67 ± 1.35 (*3*)	1.39 ± 0.29* (*3*)	-11.2 ± 3.42% (*3*)

Table S9.
Cooperativity of the D1R PAMs with selective mutations of the upper and/or lower TM1-TM7 allosteric sites. Curve parameters were determined as described in **Fig. 2G**. Data represent mean ± SEM values with the number of experiments (*n*) provided in parentheses. The EC_{50} fold-shift values are provided for informational purposes and were derived by dividing the EC_{50} of the DA control curve by the EC_{50} of the DA curve in the presence of PAMs for each experiment, which were then averaged. Differences between the EC_{50} values were evaluated for statistical significance using a one-way ANOVA with Šídák's multiple comparisons post hoc test. Where indicated, * $p < 0.05$ for single PAMs versus DA alone, † $p < 0.05$ for double PAM combinations versus the relevant single PAMs, and ‡ $p < 0.05$ for triple PAM combinations vs. the relevant double PAM combinations.

	**D1R Constructs**			
**Condition**	**WT D1R**<br>EC_{50} (nM)<br>(Fold-Shift)	**Upper Cluster**<br>EC_{50} (nM)<br>(Fold-Shift)	**Lower Cluster**<br>EC_{50} (nM)<br>(Fold-Shift)	**Upper + Lower**<br>EC_{50} (nM)<br>(Fold-Shift)
**DA**	12,200 ± 1,290 (*20*)	7,750 ± 1,110 (*6*)	3,660 ± 550 (*11*)	1,150 ± 199 (*8*)
**+ 30 µM UNC9815**	1,340 ± 180*<br>(10.0 ± 1.13) (*20*)	15,100 ± 1,790*<br>(0.53 ± 0.07) (*6*)	186 ± 23.3*<br>(23.6 ± 4.89) (*11*)	1,770 ± 379*<br>(0.64 ± 0.06) (*7*)
**+ 50 µM LY3154207**	1,520 ± 313*<br>(10.6 ± 1.93) (*20*)	1,360 ± 159*<br>(6.00 ± 0.87) (*6*)	307 ± 70.3*<br>(17.0 ± 4.01) (*11*)	85.1 ± 15.2*<br>(15.3 ± 3.16) (*8*)
**+ 20 µM BMS-A1**	847 ± 80.8*<br>(15.4 ± 1.64) (*19*)	869 ± 109*<br>(9.12 ± 0.96) (*6*)	3,050 ± 429<br>(1.31 ± 0.17) (*11*)	1,150 ± 136<br>(1.10 ± 0.26) (*8*)
**UNC9815 + BMS-A1**	67.3 ± 8.39†<br>(215 ± 24.6) (*19*)	2,700 ± 419†<br>(3.04 ± 0.40) (*6*)	167 ± 19.5<br>(24.5 ± 4.51) (*11*)	1,820 ± 190<br>(0.63 ± 0.10) (*8*)
**LY3154207 + BMS-A1**	72.9 ± 6.23†<br>(182 ± 22.6) (*20*)	97.0 ± 19.7†<br>(86.4 ± 11.9) (*6*)	347 ± 79.9<br>(17.8 ± 6.62) (*11*)	124 ± 17.2<br>(9.25 ± 1.16) (*7*)
**UNC9815 + LY3154207**	132 ± 15.3†<br>(115 ± 21.9) (*19*)	2,420 ± 398†<br>(3.47 ± 0.53) (*6*)	20.3 ± 3.11†<br>(198 ± 43.3) (*10*)	125 ± 30.5<br>(10.6 ± 3.42) (*6*)
**UNC9815 + LY3154207 + BMS-A1**	12.0 ± 2.19‡<br>(1,500 ± 265) (*20*)	355 ± 76.3‡<br>(23.4 ± 2.56) (*6*)	40.9± 11.2‡<br>(118± 25.7) (*7*)	153 ± 32.9<br>(8.39 ± 1.03) (*8*)

Table S10.
Summary of the MD simulations using various combinations of orthosteric ligands (SKF81297 and DA) and allosteric ligands (LY3154207, UNC9815, UNC10062 and BMS-A1), categorized by G-protein coupling state. LY3: LY3154207. Related to **fig. S11 and S12, Figs. 4C and F**.

			**Between TM1 and TM7**			
**G-protein Bound**	**Orthosteric Ligand**	**Above IL2**	**Upper Pocket**	**Lower Pocket**	**# Trajectories**	**Time (µs)**
Yes	SKF	LY3	-	BMS (n)	4	3.85
				BMS (+)	2	0.93
Yes	SKF	LY3	UNC62	-	2	4.11
Yes	SKF	LY3	UNC62	BMS (n)	5	3.62
Yes	SKF	-	UNC62	-	4	3.33
Yes	SKF	-	-	BMS (n)	3	3.60
Yes	LDP	LY3	*UNC15*	-	2	2.04
Yes	LDP	LY3	*UNC15*	BMS (n)	6	4.03
Yes	LDP	-	*UNC15*	-	4	4.02
No	SKF	-	-	-	3	3.60
No	SKF	LY3	-	-	3	3.60
No	SKF	LY3	*UNC62*	BMS (n)	3	3.36
**Total**					**41**	**40.09**

Table S11.

Effects of swapping D1R and D5R residues S36$^{1.45}$ and W53$^{1.45}$ on BMS-A1 PAM activity using β-arrestin recruitment assays. Curve parameters are from **Fig. 3C-D**. Data represent mean ± SEM values from four independent experiments, each performed in duplicate. Fold-shift values were derived by dividing the EC_{50} of the DA control curve by the EC_{50} of the DA curve in the presence of BMS-A1 for each experiment. The calculated fold-shifts for each experiment were then averaged. The Emax values represent the percent of the control DA Emax values and are shown for informational purposes. With each receptor construct, the EC_{50} value in the presence of BMS-A1 was compared to the EC_{50} value in the absence of BMS-A1 using a *t*-test. * $p < 0.05$, ** $p < 0.01$, *** $p < 0.001$. The EC_{50} fold-shifts between corresponding WT and mutant receptors were compared using a *t*-test. Significance is represented with † $p < 0.05$.

	**DA**	**DA + 20 μM BMS-A1**		
**DAR Construct**	EC_{50} (μM)	EC_{50} (μM)	EC_{50} Fold-Shift	Emax (% DA Emax)
**D1R-WT**	2.19 ± 0.17	0.24 ± 0.13**	15.8 ± 4.16	153 ± 13.2%
**D1R-S36W**	1.14 ± 0.24	3.36 ± 1.43*	0.41 ± 0.07†	126 ± 4.76%
**D5R-WT**	0.13 ± 0.04	0.22 ± 0.06	0.77 ± 0.37	96.3 ± 10.4%
**D5R-W53S**	0.12 ± 0.03	0.006 ± 0.0005***	17.6 ± 3.21†	117 ± 3.35%

REFERENCES

1. M. Zhang, T. Chen, X. Lu, X. Lan, Z. Chen, S. Lu, G protein-coupled receptors (GPCRs): Advances in structures, mechanisms, and drug discovery. *Signal Transduct. Target. Ther.* **9**, 88 (2024).

2. J. S. Lorente, A. V. Sokolov, G. Ferguson, H. B. Schiöth, A. S. Hauser, D. E. Gloriam, GPCR drug discovery: New agents, targets and indications. *Nat. Rev. Drug Discov.* **24**, 458–479 (2025).

3. J.-M. Beaulieu, S. Espinoza, R. R. Gainetdinov, Dopamine receptors—IUPHAR review 13. *Br. J. Pharmacol.* **172**, 1–23 (2015).

4. J.-M. Beaulieu, R. R. Gainetdinov, The physiology, signaling, and pharmacology of dopamine receptors. *Pharmacol. Rev.* **63**, 182–217 (2011).

5. S. A. Castner, P. S. Goldman-Rakic, Enhancement of working memory in aged monkeys by a sensitizing regimen of dopamine D1 receptor stimulation. *J. Neurosci.* **24**, 1446–1450 (2004).

6. A. Hall, L. Provins, A. Valade, Novel strategies to activate the dopamine D1 receptor: Recent advances in orthosteric agonism and positive allosteric modulation. *J. Med. Chem.* **62**, 128–140 (2019).

7. P. S. Goldman-Rakic, S. A. Castner, T. H. Svensson, L. J. Siever, G. V. Williams, Targeting the dopamine D1 receptor in schizophrenia: Insights for cognitive dysfunction. *Psychopharmacology (Berl)* **174**, 3–16 (2004).

8. D. E. Felsing, M. K. Jain, J. A. Allen, Advances in dopamine D1 receptor ligands for neurotherapeutics. *Curr. Top. Med. Chem.* **19**, 1365–1380 (2019).

9. S. D. Iversen, L. L. Iversen, Dopamine: 50 years in perspective. *Trends Neurosci.* **30**, 188–193 (2007).

10. C. Zeng, M. Zhang, L. D. Asico, G. M. Eisner, P. A. Jose, The dopaminergic system in hypertension. *Clin. Sci.* **112**, 583–597 (2007).

11. P. Jayachandran, F. Battaglin, C. Strelez, A. Lenz, S. Algaze, S. Soni, J. H. Lo, Y. Yang, J. Millstein, W. Zhang, J. C. Shih, J. Lu, S. M. Mumenthaler, D. Spicer, J. Neman, E. T. Roussos Torres, H.-J. Lenz, Breast cancer and neurotransmitters: Emerging insights on mechanisms and therapeutic directions. *Oncogene* **42**, 627–637 (2023).

12. H. Sanada, L. D. Asico, S. Shigetomi, K. Tanaka, S. Niimura, H. Watanabe, D. S. Goldstein, R. A. Felder, The effect of docarpamine, a dopamine pro-drug, on blood pressure and catecholamine levels in spontaneously hypertensive rats. *Clin. Exp. Hypertens.* **22**, 419–429 (2000).

13. B. J. Melancon, C. R. Hopkins, M. R. Wood, K. A. Emmitte, C. M. Niswender, A. Christopoulos, P. J. Conn, C. W. Lindsley, Allosteric modulation of seven transmembrane spanning receptors: Theory, practice, and opportunities for central nervous system drug discovery. *J. Med. Chem.* **55**, 1445–1464 (2012).

14. P. J. Conn, A. Christopoulos, C. W. Lindsley, Allosteric modulators of GPCRs: A novel approach for the treatment of CNS disorders. *Nat. Rev. Drug Discov.* **8**, 41–54 (2009).

15. H. H. Nickols, P. J. Conn, Development of allosteric modulators of GPCRs for treatment of CNS disorders. *Neurobiol. Dis.* **61**, 55–71 (2014).

16. P. J. Conn, C. W. Lindsley, J. Meiler, C. M. Niswender, Opportunities and challenges in the discovery of allosteric modulators of GPCRs for treating CNS disorders. *Nat. Rev. Drug Discov.* **13**, 692–708 (2014).

17. F. Girmaw, Review on allosteric modulators of dopamine receptors so far. *Health Sci. Rep.* **7**, e1984 (2024).

18. K. A. Svensson, B. A. Heinz, J. M. Schaus, J. P. Beck, J. Hao, J. H. Krushinski, M. R. Reinhard, M. P. Cohen, S. L. Hellman, B. G. Getman, X. Wang, M. M. Menezes, D. L. Maren, J. F. Falcone, W. H. Anderson, R. A. Wright, S. M. Morin, K. L. Knopp, B. L. Adams, B. Rogovoy, I. Okun, T. M. Suter, M. A. Statnick, D. R. Gehlert, D. L. Nelson, V. L. Lucaites, R. Emkey, N. W. DeLapp, T. R. Wiernicki, J. W. Cramer, C. R. Yang, R. F. Bruns, An allosteric potentiator of the

dopamine d1 receptor increases locomotor activity in human D1 knock-in mice without causing stereotypy or tachyphylaxis. *J. Pharmacol. Exp. Ther.* **360**, 117–128 (2017).

19. D. R. Sibley, K. D. Luderman, R. B. Free, L. Shi, Novel Cryo-EM structures of the D1 dopamine receptor unlock its therapeutic potential. *Signal Transduct. Target. Ther.* **6**, 205 (2021).

20. A. F. T. Arnsten, R. R. Girgis, D. L. Gray, R. B. Mailman, Novel dopamine therapeutics for cognitive deficits in schizophrenia. *Biol. Psychiatry* **81**, 67–77 (2017).

21. S. Vijayraghavan, M. Wang, S. G. Birnbaum, G. V. Williams, A. F. Arnsten, Inverted-U dopamine D1 receptor actions on prefrontal neurons engaged in working memory. *Nat. Neurosci.* **10**, 376–384 (2007).

22. K. A. Svensson, J. Hao, R. F. Bruns, Positive allosteric modulators of the dopamine D1 receptor: A new mechanism for the treatment of neuropsychiatric disorders. *Adv. Pharmacol.* **86**, 273–305 (2019).

23. X. Wang, B. A. Heinz, Y.-W. Qian, J. H. Carter, R. A. Gadski, L. S. Beavers, S. P. Little, C. R. Yang, J. P. Beck, J. Hao, J. M. Schaus, K. A. Svensson, R. F. Bruns, Intracellular binding site for a positive allosteric modulator of the dopamine D1 receptor. *Mol. Pharmacol.* **94**, 1232–1245 (2018).

24. K. D. Luderman, J. L. Conroy, R. B. Free, N. Southall, M. Ferrer, M. Sanchez-Soto, A. E. Moritz, B. K. A. Willette, T. J. Fyfe, P. Jain, S. Titus, L. A. Hazelwood, J. Aubé, J. R. Lane, K. J. Frankowski, D. R. Sibley, Identification of positive allosteric modulators of the D1 dopamine receptor that act at diverse binding sites. *Mol. Pharmacol.* **94**, 1197–1209 (2018).

25. M. A. Lewis, L. Hunihan, J. Watson, R. G. Gentles, S. Hu, Y. Huang, J. Bronson, J. E. Macor, B. R. Beno, M. Ferrante, A. Hendricson, R. J. Knox, T. F. Molski, Y. Kong, M. E. Cvijic, K. L. Rockwell, M. R. Weed, A. M. Cacace, R. S. Westphal, A. Alt, J. M. Brown, Discovery of D1 dopamine receptor positive allosteric modulators: Characterization of pharmacology and

identification of residues that regulate species selectivity. *J. Pharmacol. Exp. Ther.* **354**, 340–349 (2015).

26. A. Desai, L. Benner, R. Wu, L. Gertsik, T. Uz, G. J. Marek, T. Zhu, Pharmacokinetics of ASP4345 from single ascending-dose and multiple ascending-dose phase I studies. *Clin. Pharmacokinet.* **60**, 79–88 (2021).

27. A. Desai, L. Benner, R. Wu, L. Gertsik, P. Maruff, G. A. Light, T. Uz, G. J. Marek, T. Zhu, Phase 1 randomized study on the safety, tolerability, and pharmacodynamic cognitive and electrophysiological effects of a dopamine D1 receptor positive allosteric modulator in patients with schizophrenia. *Neuropsychopharmacology* **46**, 1145–1151 (2021).

28. A. P. McCarthy, K. A. Svensson, E. Shanks, C. Brittain, B. J. Eastwood, W. Kielbasa, K. M. Biglan, K. A. Wafford, The dopamine D1 receptor positive allosteric modulator mevidalen (LY3154207) enhances wakefulness in the humanized D1 mouse and in sleep-deprived healthy male volunteers. *J. Pharmacol. Exp. Ther.* **380**, 143–152 (2022).

29. K. Biglan, L. Munsie, K. A. Svensson, P. Ardayfio, M. Pugh, J. Sims, M. Brys, Safety and efficacy of mevidalen in lewy body dementia: A phase 2, randomized, placebo-controlled Trial. *Mov. Disord.* **37**, 513–524 (2022).

30. J. Hao, J. P. Beck, J. M. Schaus, J. H. Krushinski, Q. Chen, C. D. Beadle, P. Vidal, M. R. Reinhard, B. A. Dressman, S. M. Massey, S. L. Boulet, M. P. Cohen, B. M. Watson, D. Tupper, K. M. Gardinier, J. Myers, A. M. Johansson, J. Richardson, D. S. Richards, E. J. Hembre, D. M. Remick, D. A. Coates, R. M. Bhardwaj, B. A. Diseroad, D. Bender, G. Stephenson, C. D. Wolfangel, N. Diaz, B. G. Getman, X.-S. Wang, B. A. Heinz, J. W. Cramer, X. Zhou, D. L. Maren, J. F. Falcone, R. A. Wright, S. N. Mitchell, G. Carter, C. R. Yang, R. F. Bruns, K. A. Svensson, Synthesis and pharmacological characterization of 2-(2,6-dichlorophenyl)-1-((1S,3R)-5-(3-hydroxy-3-methylbutyl)-3-(hydroxymethyl)-1-methyl-3,4-dihydroisoquinolin-2(1H)-yl)ethan-1-one (LY3154207), a potent, subtype-selective, and orally available positive allosteric modulator of the human dopamine D1 receptor. *J. Med. Chem.* **62**, 8711–8732 (2019).

31. R. F. Bruns, S. N. Mitchell, K. A. Wafford, A. J. Harper, E. A. Shanks, G. Carter, M. J. O'Neill, T. K. Murray, B. J. Eastwood, J. M. Schaus, J. P. Beck, J. Hao, J. M. Witkin, X. Li, E. Chernet, J. S. Katner, H. Wang, J. W. Ryder, M. E. Masquelin, L. K. Thompson, P. L. Love, D. L. Maren, J. F. Falcone, M. M. Menezes, L. Zhang, C. R. Yang, K. A. Svensson, Preclinical profile of a dopamine D1 potentiator suggests therapeutic utility in neurological and psychiatric disorders. *Neuropharmacology* **128**, 351–365 (2018).

32. K. Luderman, R. B. Free, F. Koenigsberg, J. Aubé, K. Frankowski, K. Neve, D. Sibley, Characterization of a D1 dopamine receptor positive allosteric modulator with a novel binding site. *FASEB J.* **35**, 02361 (2021).

33. X. Wang, E. J. Hembre, P. J. Goldsmith, J. P. Beck, K. A. Svensson, F. S. Willard, R. F. Bruns, Mutual cooperativity of three allosteric sites on the dopamine D1 receptor. *Mol. Pharmacol.* **103**, 176–187 (2023).

34. Y. Zhuang, B. Krumm, H. Zhang, X. E. Zhou, Y. Wang, X.-P. Huang, Y. Liu, X. Cheng, Y. Jiang, H. Jiang, C. Zhang, W. Yi, B. L. Roth, Y. Zhang, H. E. Xu, Mechanism of dopamine binding and allosteric modulation of the human D1 dopamine receptor. *Cell Res.* **31**, 593–596 (2021).

35. J. N. Hanson, A. N. Nilson, E. Cohen, F. Wang, J. Rayadurgam, K. D. Luderman, R. B. Free, L. Shi, K. J. Frankowski, D. R. Sibley, Characterization of the binding pocket for a novel positive allosteric modulator of the D1 dopamine receptor. *J. Pharmacol. Exp. Ther.* **389**, 344 (2024).

36. Y. Zhuang, P. Xu, C. Mao, L. Wang, B. Krumm, X. E. Zhou, S. Huang, H. Liu, X. Cheng, X.-P. Huang, D.-D. Shen, T. Xu, Y.-F. Liu, Y. Wang, J. Guo, Y. Jiang, H. Jiang, K. Melcher, B. L. Roth, Y. Zhang, C. Zhang, H. E. Xu, Structural insights into the human D1 and D2 dopamine receptor signaling complexes. *Cell* **184**, 931–942.e18 (2021).

37. P. Xiao, W. Yan, L. Gou, Y.-N. Zhong, L. Kong, C. Wu, X. Wen, Y. Yuan, S. Cao, C. Qu, X. Yang, C.-C. Yang, A. Xia, Z. Hu, Q. Zhang, Y.-H. He, D.-L. Zhang, C. Zhang, G.-H. Hou, H. Liu, L. Zhu, P. Fu, S. Yang, D. M. Rosenbaum, J.-P. Sun, Y. Du, L. Zhang, X. Yu, Z. Shao,

Ligand recognition and allosteric regulation of DRD1-Gs signaling complexes. *Cell* **184**, 943–956.e18 (2021).

38. X. Teng, S. Chen, Y. Nie, P. Xiao, X. Yu, Z. Shao, S. Zheng, Ligand recognition and biased agonism of the D1 dopamine receptor. *Nat. Commun.* **13**, 3186 (2022).

39. A. Goldberg, B. Xie, L. Shi, The molecular mechanism of positive allosteric modulation at the dopamine D1 receptor. *Int. J. Mol. Sci.* **24**, 12848 (2023).

40. H. D. Lim, D. Bartuzi, A. C. Keen, C. Rauffenbart, J. Glenn, S. J. Charlton, S. Lovera, Z. A. Sands, A. Ates, M. Wood, M. Canals, J. A. Javitch, J. Carlsson, J. R. Lane, Identification of a lipid-exposed extrahelical binding site for positive allosteric modulators of the dopamine D_2 receptor. *ACS Chem. Nerosci.* **16**, 2295–2311 (2025).

41. A. T. N. Nguyen, N. Panel, D. D. Vo, B. S. Thai, L. Y. Chia, C. S. Lu, S. D. Hellyer, M. Langiu, M. Jörg, K. J. Gregory, J. Kihlberg, P. J. White, P. J. Scammells, A. Christopoulos, J. Carlsson, L. T. May, Structure-based discovery of positive allosteric modulators of the A_1 adenosine receptor. *Proc. Natl. Acad. Sci. U.S.A.* **122**, e2421687122 (2025).

42. C. L. Fisher, M. Pavan, V. Salmaso, R. F. Keyes, T. C. Wan, B. Pradhan, Z.-G. Gao, B. C. Smith, K. A. Jacobson, J. A. Auchampach, Extrahelical binding site for a 1H-Imidazo[4,5-c]quinolin-4-amine A_3 adenosine receptor positive allosteric modulator on helix 8 and distal portions of transmembrane domains 1 and 7. *Mol. Pharmacol.* **105**, 213–223 (2024).

43. X. Zhang, A.-A. Guseinov, L. Jenkins, A. Valentini, S. Marsango, K. Schultz-Knudsen, T. Ulven, E. R. Ulven, I. G. Tikhonova, G. Milligan, C. Zhang, Allosteric modulation and biased signalling at free fatty acid receptor 2. *Nature* **643**, 1428–1438 (2025).

44. P. Xu, S. Huang, B. E. Krumm, Y. Zhuang, C. Mao, Y. Zhang, Y. Wang, X.-P. Huang, Y.-F. Liu, X. He, H. Li, W. Yin, Y. Jiang, Y. Zhang, B. L. Roth, H. E. Xu, Structural genomics of the human dopamine receptor system. *Cell Res.* **33**, 604–616 (2023).

45. L. Shi, M. M. Simpson, J. A. Ballesteros, J. A. Javitch, The first transmembrane segment of the dopamine D2 receptor: Accessibility in the binding-site crevice and position in the transmembrane bundle. *Biochemistry* **40**, 12339–12348 (2001).

46. X. Zhang, Y. Zheng, H. Liu, J. Hou, L. Fan, X. He, J. Sun, T. Liu, J. Zhou, R. Lei, M. Li, W. Hu, X. Cheng, S. Wang, X. Xie, H. E. Xu, S. Guo, Y. Zhuang, Molecular principles for graded activation of dopamine D1 receptor. bioRxiv 741251 [Preprint] (2026); https://doi.org/10.64898/2026.07.28.741251.

47. M. Michino, C. A. Boateng, P. Donthamsetti, H. Yano, O. M. Bakare, A. Bonifazi, M. P. Ellenberger, T. M. Keck, V. Kumar, C. Zhu, R. Verma, J. R. Deschamps, J. A. Javitch, A. H. Newman, L. Shi, Toward understanding the structural basis of partial agonism at the dopamine D_3 receptor. *J. Med. Chem.* **60**, 580–593 (2017).

48. K. H. Lee, J. J. Manning, J. Javitch, L. Shi, A Novel “Activation switch” motif common to all aminergic receptors. *J. Chem. Inf. Model.* **63**, 5001–5017 (2023).

49. C.-M. Suomivuori, N. R. Latorraca, L. M. Wingler, S. Eismann, M. C. King, A. L. W. Kleinhenz, M. A. Skiba, D. P. Staus, A. C. Kruse, R. J. Lefkowitz, R. O. Dror, Molecular mechanism of biased signaling in a prototypical G protein-coupled receptor. *Science* **367**, 881–887 (2020).

50. A. Zhang, J. L. Neumeyer, R. J. Baldessarini, Recent progress in development of dopamine receptor subtype-selective agents: potential therapeutics for neurological and psychiatric disorders. *Chem. Rev.* **107**, 274–302 (2007).

51. S. Shen, C. Zhao, C. Wu, S. Sun, Z. Li, W. Yan, Z. Shao, Allosteric modulation of G protein-coupled receptor signaling. *Front. Endocrinol.* **14**, 1137604 (2023).

52. S. K. Huang, O. Almurad, R. J. Pejana, Z. A. Morrison, A. Pandey, L.-P. Picard, M. Nitz, A. Sljoka, R. S. Prosser, Allosteric modulation of the adenosine A_{2A}, receptor by cholesterol. *eLife* **11**, e73901 (2022).

53. E. van Aalst, B. J. Wylie, Cholesterol is a dose-dependent positive allosteric modulator of CCR3 ligand affinity and g protein coupling. *Front. Mol. Biosci.* **8**, 724603 (2021).

54. J. Jakubik, E. E. El-Fakahany, Allosteric modulation of GPCRs of class A by cholesterol. *Int. J. Mol. Sci.* **22**, 1953 (2021).

55. B. Marlow, A. Vogel, G. Kuenze, M. Pankonin, F. Reinhardt, P. F. Stadler, P. W. Hildebrand, J. Meiler, Cholesterol allosteric modulation of the oxytocin receptor. *Biophys. J.* **124**, 1930–1943 (2025).

56. S. Filipek, Molecular switches in GPCRs. *Curr. Opin. Struct. Biol.* **55**, 114–120 (2019).

57. D. Hilger, M. Masureel, B. K. Kobilka, Structure and dynamics of GPCR signaling complexes. *Nat. Struct. Mol. Biol.* **25**, 4–12 (2018).

58. N. R. Latorraca, A. J. Venkatakrishnan, R. O. Dror, GPCR dynamics: Structures in motion. *Chem. Rev.* **117**, 139–155 (2017).

59. X. Liu, A. Masoudi, A. W. Kahsai, L.-Y. Huang, B. Pani, D. P. Staus, P. J. Shim, K. Hirata, R. K. Simhal, A. M. Schwalb, P. K. Rambarat, S. Ahn, R. J. Lefkowitz, B. K. Kobilka, Mechanism of beta2AR regulation by an intracellular positive allosteric modulator. *Science* **364**, 1283–1287 (2019).

60. X. Liu, J. Kaindl, M. Korczynska, A. Stößel, D. Dengler, M. Stanek, H. Hübner, M. J. Clark, J. Mahoney, R. A. Matt, X. Xu, K. Hirata, B. K. Shoichet, R. K. Sunahara, B. K. Kobilka, P. Gmeiner, An allosteric modulator binds to a conformational hub in the beta(2) adrenergic receptor. *Nat. Chem. Biol.* **16**, 749–755 (2020).

61. X. Liu, S. Ahn, A. W. Kahsai, K.-C. Meng, N. R. Latorraca, B. Pani, A. J. Venkatakrishnan, A. Masoudi, W. I. Weis, R. O. Dror, X. Chen, R. J. Lefkowitz, B. K. Kobilka, Mechanism of intracellular allosteric β_2AR antagonist revealed by x-ray crystal structure. *Nature* **548**, 480–484 (2017).

62. W. A. C. Burger, J. I. Mobbs, B. Rana, J. Wang, K. Joshi, P. R. Gentry, M. Yeasmin, H. Venugopal, A. M. Bender, C. W. Lindsley, Y. Miao, A. Christopoulos, C. Valant, D. M. Thal, Cryo-EM reveals an extrahelical allosteric binding site at the M_5 mAChR. *Nat. Commun.* **16**, 7046 (2025).

63. W. A. C. Burger, P. R. Gentry, A. E. Berizzi, Z. Vuckovic, E. T. van der Westhuizen, G. Thompson, M. Yeasmin, C. W. Lindsley, P. M. Sexton, C. J. Langmead, A. B. Tobin, A. Christopoulos, C. Valant, D. M. Thal, Identification of a novel allosteric site at the M_5 muscarinic acetylcholine receptor. *ACS Chem. Nerosci.* **12**, 3112–3123 (2021).

64. W. Huang, N. Jin, J. Guo, C. Shen, C. Xu, K. Xi, L. Bonhomme, R. B. Quast, D.-D. Shen, J. Qin, Y.-R. Liu, Y. Song, Y. Gao, E. Margeat, P. Rondard, J.-P. Pin, Y. Zhang, J. Liu, Structural basis of orientated asymmetry in a mGlu heterodimer. *Nat. Commun.* **15**, 10345 (2024).

65. X. Wang, M. Wang, T. Xu, Y. Feng, Q. Shao, S. Han, X. Chu, Y. Xu, S. Lin, Q. Zhao, B. Wu, Structural insights into dimerization and activation of the mGlu2-mGlu3 and mGlu2-mGlu4 heterodimers. *Cell Res.* **33**, 762–774 (2023).

66. S. Lin, S. Han, X. Cai, Q. Tan, K. Zhou, D. Wang, X. Wang, J. Du, C. Yi, X. Chu, A. Dai, Y. Zhou, Y. Chen, Y. Zhou, H. Liu, J. Liu, D. Yang, M.-W. Wang, Q. Zhao, B. Wu, Structures of Gi-bound metabotropic glutamate receptors mGlu2 and mGlu4. *Nature* **594**, 583–588 (2021).

67. J. Du, D. Wang, H. Fan, C. Xu, L. Tai, S. Lin, S. Han, Q. Tan, X. Wang, T. Xu, H. Zhang, X. Chu, C. Yi, P. Liu, X. Wang, Y. Zhou, J.-P. Pin, P. Rondard, H. Liu, J. Liu, F. Sun, B. Wu, Q. Zhao, Structures of human mGlu2 and mGlu7 homo- and heterodimers. *Nature* **594**, 589–593 (2021).

68. G. Cannone, L. Berto, F. Malhaire, G. Ferguson, A. Fouillen, S. Balor, J. Font-Ingles, A. Llebaria, C. Goudet, A. Kotecha, K. R. Vinothkumar, G. Lebon, Conformational diversity in class C GPCR positive allosteric modulation. *Nat. Commun.* **16**, 619 (2025).

69. A. Strauss, A. J. Gonzalez-Hernandez, J. Lee, N. Abreu, P. Selvakumar, L. Salas-Estrada, M. Kristt, A. Arefin, K. Huynh, D. C. Marx, K. Gilliland, B. J. Melancon, M. Filizola, J. Meyerson,

J. Levitz, Structural basis of positive allosteric modulation of metabotropic glutamate receptor activation and internalization. *Nat. Commun.* **15**, 6498 (2024).

70. J. Zhao, Y. Deng, Z. Xu, C. Xu, C. Zhao, Z. Li, H. Sun, X. Tian, Y. Song, M. Cimadevila, H. Wang, Y. Liu, X. Zhang, Y. Chen, S. Sun, X. Yong, L. Su, Y. He, Y. Zhong, H. Yang, J.-P. Pin, W. Yan, Z. Shao, J. Liu, Structural characterization of five functional states of metabotropic glutamate receptor 8. *Mol. Cell* **85**, 3460–3473.e6 (2025).

71. F. Wang, J. N. Hanson, S. T. Dimova, J. Rayadurgam, C. J. Fulton, A. E. Moritz, A. N. Nilson, W. A. Hearne, C.-H. Chen, R. H. Gumpper, D. R. Sibley, K. J. Frankowski, Synthesis and systematic evaluation of four stereoisomers of BMS compound A enabled by a diastereoselective cyclopropanation on a chiral ketal intermediate. *RSC Adv.* **16**, 39744–39756 (2026).

72. C. Xing, Y. Zhuang, T.-H. Xu, Z. Feng, X. E. Zhou, M. Chen, L. Wang, X. Meng, Y. Xue, J. Wang, H. Liu, T. F. McGuire, G. Zhao, K. Melcher, C. Zhang, H. E. Xu, X.-Q. Xie, Cryo-EM structure of the human cannabinoid receptor CB2-Gi signaling complex. *Cell* **180**, 645–654.e13 (2020).

73. J. Duan, D.-D. Shen, X. E. Zhou, P. Bi, Q.-F. Liu, Y.-X. Tan, Y.-W. Zhuang, H.-B. Zhang, P.-Y. Xu, S.-J. Huang, S.-S. Ma, X.-H. He, K. Melcher, Y. Zhang, H. E. Xu, Y. Jiang, Cryo-EM structure of an activated VIP1 receptor-G protein complex revealed by a NanoBiT tethering strategy. *Nat. Commun.* **11**, 4121 (2020).

74. D. Kimanius, L. Dong, G. Sharov, T. Nakane, S. H. W. Scheres, New tools for automated cryo-EM single-particle analysis in RELION-4.0. *Biochem. J.* **478**, 4169–4185 (2021).

75. A. Punjani, J. L. Rubinstein, D. J. Fleet, M. A. Brubaker, cryoSPARC: Algorithms for rapid unsupervised cryo-EM structure determination. *Nat. Methods* **14**, 290–296 (2017).

76. A. Punjani, H. Zhang, D. J. Fleet, Non-uniform refinement: Adaptive regularization improves single-particle cryo-EM reconstruction. *Nat. Methods* **17**, 1214–1221 (2020).

77. P. Emsley, K. Cowtan, Coot: Model-building tools for molecular graphics. *Acta Crystallogr. D Biol. Crystallogr.* **60**, 2126–2132 (2004).

78. P. D. Adams, P. V. Afonine, G. Bunkóczi, V. B. Chen, I. W. Davis, N. Echols, J. J. Headd, L.-W. Hung, G. J. Kapral, R. W. Grosse-Kunstleve, A. J. McCoy, N. W. Moriarty, R. Oeffner, R. J. Read, D. C. Richardson, J. S. Richardson, T. C. Terwilliger, P. H. Zwart, PHENIX: A comprehensive Python-based system for macromolecular structure solution. *Acta Crystallogr. D Biol. Crystallogr.* **66**, 213–221 (2010).

79. A. E. Moritz, R. B. Free, W. S. Weiner, E. O. Akano, D. Gandhi, A. Abramyan, T. M. Keck, M. Ferrer, X. Hu, N. Southall, J. Steiner, J. Aubé, L. Shi, K. J. Frankowski, D. R. Sibley, Discovery, optimization, and characterization of ML417: A novel and highly selective D_3 dopamine receptor agonist. *J. Med. Chem.* **63**, 5526–5567 (2020).

80. Y. Namkung, C. Le Gouill, V. Lukashova, H. Kobayashi, M. Hogue, E. Khoury, M. Song, M. Bouvier, S. A. Laporte, Monitoring G protein-coupled receptor and β-arrestin trafficking in live cells using enhanced bystander BRET. *Nat. Commun.* **7**, 12178 (2016).

81. A. E. Moritz, F. Wang, N. S. Madaras, A. M. Kelley, K. K. Snyder, D. Gandhi, L. R. Inbody, E. O. Akano, L. Shi, J. R. Lane, R. B. Free, K. J. Frankowski, D. R. Sibley, Discovery, characterization, and optimization of a novel positive allosteric modulator-antagonist of the D3 dopamine receptor. *J. Med. Chem.* **68**, 16691–16749 (2025).

82. L. I. Jiang, J. Collins, R. Davis, K.-M. Lin, D. DeCamp, T. Roach, R. Hsueh, R. A. Rebres, E. M. Ross, R. Taussig, I. Fraser, P. C. Sternweis, Use of a cAMP BRET sensor to characterize a novel regulation of cAMP by the sphingosine 1-phosphate/G13 pathway. *J. Biol. Chem.* **282**, 10576–10584 (2007).

83. R. H. J. Olsen, J. F. DiBerto, J. G. English, A. M. Glaudin, B. E. Krumm, S. T. Slocum, T. Che, A. C. Gavin, J. D. McCorvy, B. L. Roth, R. T. Strachan, TRUPATH, an open-source biosensor platform for interrogating the GPCR transducerome. *Nat. Chem. Biol.* **16**, 841–849 (2020).

84. B. Webb, A. Sali, Comparative protein structure modeling using MODELLER. *Curr. Protoc. Bioinformatics* **54**, 5.6.1–5.6.37 (2016).

85. M. Y. Shen, A. Sali, Statistical potential for assessment and prediction of protein structures. *Protein Sci.* **15**, 2507–2524 (2006).

86. M. H. Olsson, Protein electrostatics and pKa blind predictions; contribution from empirical predictions of internal ionizable residues. *Proteins* **79**, 3333–3345 (2011).

87. R. C. Johnston, K. Yao, Z. Kaplan, M. Chelliah, K. Leswing, S. Seekins, S. Watts, D. Calkins, J. Chief Elk, S. V. Jerome, M. P. Repasky, J. C. Shelley, Epik: pKa and protonation state prediction through machine learning. *J. Chem. Theory Comput.* **19**, 2380–2388 (2023).

88. W. Humphrey, A. Dalke, K. Schulten, VMD: Visual molecular dynamics. *J. Mol. Graph.* **14**, 33–38 (1996).

89. X. Liu, X. Rong, S. Liu, Y. Lan, Q. Liu, Cobalt-catalyzed desymmetric isomerization of exocyclic olefins. *J. Am. Chem. Soc.* **143**, 20633–20639 (2021).

90. N. B. Zuckerman, A. S. Myers, T. K. Quan, W. M. Bray, R. S. Lokey, G. A. Hartzog, J. P. Konopelski, Structural determination of NSC 670224, synthesis of analogues and biological evaluation. *ChemMedChem* **7**, 761–765 (2012).

91. J. A. Ballesteros, H. Weinstein, "Integrated methods for the construction of three-dimensional models and computational probing of structure-function relations in g protein-coupled receptors," in *Methods in Neurosciences*, S. C. Sealfon, Ed. (Academic Press, 1995), vol. **25**, pp. 366–428.
